# Ecosystem biomonitoring with eDNA: metabarcoding across the tree of life in a tropical marine environment

**DOI:** 10.1038/s41598-017-12501-5

**Published:** 2017-09-25

**Authors:** Michael Stat, Megan J. Huggett, Rachele Bernasconi, Joseph D. DiBattista, Tina E. Berry, Stephen J. Newman, Euan S. Harvey, Michael Bunce

**Affiliations:** 1 Trace and Environmental DNA (TrEnD) Laboratory, Department of Environment and Agriculture, Curtin University, Perth, WA 6102 Australia; 2 Centre for Marine Ecosystems Research, School of Science, Edith Cowan University, Perth, WA 6027 Australia; 3 Western Australian Fisheries and Marine Research Laboratories, Department of Primary Industries and Regional Development, Fisheries Division, Government of Western Australia, PO Box 20, North Beach, WA 6920 Australia

## Abstract

Effective marine management requires comprehensive data on the status of marine biodiversity. However, efficient methods that can document biodiversity in our oceans are currently lacking. Environmental DNA (eDNA) sourced from seawater offers a new avenue for investigating the biota in marine ecosystems. Here, we investigated the potential of eDNA to inform on the breadth of biodiversity present in a tropical marine environment. Directly sequencing eDNA from seawater using a shotgun approach resulted in only 0.34% of 22.3 million reads assigning to eukaryotes, highlighting the inefficiency of this method for assessing eukaryotic diversity. In contrast, using ‘tree of life’ (ToL) metabarcoding and 20-fold fewer sequencing reads, we could detect 287 families across the major divisions of eukaryotes. Our data also show that the best performing ‘universal’ PCR assay recovered only 44% of the eukaryotes identified across all assays, highlighting the need for multiple metabarcoding assays to catalogue biodiversity. Lastly, focusing on the fish genus *Lethrinus*, we recovered intra- and inter-specific haplotypes from seawater samples, illustrating that eDNA can be used to explore diversity beyond taxon identifications. Given the sensitivity and low cost of eDNA metabarcoding we advocate this approach be rapidly integrated into biomonitoring programs.

## Introduction

Marine ecosystems are under increasing pressure from a variety of anthropogenic stressors, including climate change and fishing activities^1–4^. Habitat degradation, overexploited fisheries, altered food web dynamics and shifts in community composition highlights the need for effective management of the marine biome in order to preserve and manage ocean resources sustainably into the future^4,5^. A fundamental tool for providing data to support management of the marine environment is biomonitoring. By targeting indicator species or repeating surveys of specific sites, regular monitoring of the biota allows impacts affecting the abundance and/or alpha and beta diversity to be measured^6^. However, current approaches to surveying conspicuous faunal elements (e.g. baited remote underwater video, transects, or diver tows) are dependent on suitable field conditions, a particular set of skills, and are limited to the narrow portion of biodiversity recorded. Indeed, community assemblage surveys are often restricted to a single taxonomic group such as fishes or corals, or even a subset of those groups^7,8^. Considering the growing evidence that total biodiversity promotes healthy ecosystem functions and that sustaining biodiversity represents a practical framework for ecosystem-based management (EBM), there is a compelling need for more comprehensive approaches to monitoring marine biota^9–13^.

Recent advances in genomic technologies afford new opportunities across a broad range of applications in environmental science^14–18^. One such application lies in the discovery that organisms leave traces of their DNA in the environment, including seawater, and when extracted, is collectively referred to as eDNA^19–21^. Here, we refer to eDNA as all genetic material that is obtained directly from the environment as in Taberlet *et al*.^21^, which depending on sampling methodology, constitutes DNA from whole organisms (i.e. prokaryotes and microscopic eukaryotes), cellular material (i.e. blood, mucous, tissue, faeces, etc.), and that which is released from the cytoplasm as free nucleic acids. The potential utility of eDNA to advance the scientific process is broad, ranging from questions related to species detection, biodiversity assessments, population genetics, reconstruction of past flora and fauna and the detection of invasive marine species^22–26^. To date however, the capacity of eDNA to inform on ecosystem-wide patterns of biodiversity (i.e. from prokaryotes to higher-order eukaryotes), in a marine environment, remains largely unexplored.

A number of methodologies can be employed in the analysis of eDNA. One approach is environmental shotgun sequencing (ESS), which randomly sequences fragmented DNA directly from an environmental sample^27^. As ESS does not enrich target DNA, the cost associated with sequencing the entire DNA complement present in a sample is prohibitive^28^. Furthermore, ESS of genetic material recovered from seawater has mostly been applied to the study of prokaryotes^29,30^ and picoeukaryotes^31^, and so its utility for characterising eDNA originating from eukaryotes, particularly macroeukaryotes, needs further investigation.

To overcome the cost and quantity of DNA that needs to be analysed when using an ESS approach, PCR amplification of target genes (and taxa) on bulk DNA extracts from the environment can be combined with next-generation sequencing (NGS) to provide high-throughput information on the species present, a technique commonly referred to as DNA metabarcoding^32^. While this approach has proven useful in detecting a high diversity of species from a variety of environmental samples (e.g. soil and water), the influence of PCR-bias on taxonomic recovery and limited correlations to biomass of target species have led researchers to explore the utility of other PCR-free methods^33–37^. For example, gene-enrichment approaches employ synthetic probes that bind and purify target DNA of interest followed by NGS^38^. While gene-enrichment is a powerful method in detecting taxa of interest from bulk samples (e.g. synthetic mixtures of macroinvertebrates^39^), its application across the remaining taxa is currently prohibitive due to the high cost of probes that would capture target genes from all other organisms present in the environment.

The capacity of eDNA to inform on eukaryotic diversity from aquatic environments was described in 2008^20^, and yet there is no single study that we are aware of that has explored the utility of eDNA methodologies to assess marine biota at a holistic ecosystem level (i.e. across the tree of life). Accordingly, before temporal and spatial surveys using eDNA can be implemented, the scope and resolution at which metabarcoding can inform on biodiversity in the ocean needs to be validated^17^. While it has been shown previously that eDNA methodologies are superior to conventional surveying methods in detecting species within aquatic environments through rigorous *in silico* and *in vitro* (PCR) analysis of primer sets designed for specific taxa (e.g. amphibians and fish^40^), the capacity for the analysis of eDNA from seawater to broadly describe total biodiversity in any given sample requires further investigation. Therefore, in this study, we focused on a single tropical coral reef site to explore the ability of eDNA to audit marine biota across the entire tree of life. We chose to sample seawater from Coral Bay in west Australia as it resides within the World Heritage site of Ningaloo Reef, which is renowned for its rich marine biodiversity and enigmatic megafauna, is one of the world’s longest fringing coral reefs, and is therefore of high conservation importance^41^. Rather than perform rigorous tests on the detection of specific groups of taxa using eDNA^34,40,42^, we instead focused on assessing the broad potential of eDNA for auditing marine taxa. To appraise the efficacy of different methodologies for the study of eDNA in the ocean, we analysed over 23 million sequences originating from 9 L of filtered seawater and compared the diversity of taxa detected at Coral Bay using ESS and metabarcoding. We further investigated the potential of eDNA to inform on intra-species diversity using mitochondrial haplotype data, with the intention of developing approaches for the use of eDNA in measuring population diversity for a commercially targeted fish genus in Northwestern Australia^43^.

## Results and Discussion

### Shotgun sequencing of eDNA from seawater

**Figure 1:**
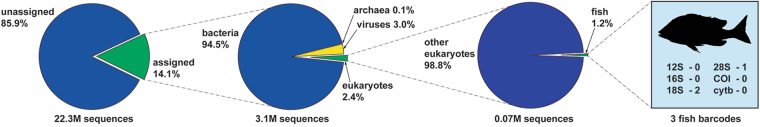
Assignment of sequences recovered from the shotgun library of eDNA collected from Coral Bay in west Australia. Pie chart segments represent the percentage of sequences that were assigned to taxa using the software MEGAN 5.11.3. Sequences that were assigned to fish were further mined for commonly used DNA barcodes (12S, 16S, 18S, 28S, COI, and cyt*b*); the number of fish barcodes identified in the dataset is displayed in the box insert.

We built a Nextera XT library from the DNA extracted from 9 L of seawater collected at Coral Bay, generating 22.3 million reads (single end 151 bp fragments) on an Ilumina NextSeq platform using an ESS approach. Of the 22.3 million DNA sequences in the shotgun library that passed quality filtering, only 14.1% could be assigned to taxa using Blastn (Fig. 1), with the highest proportion of hits assigning to bacteria (94.5%), followed by viruses (3.0%), eukaryotes (2.4%) and archaea (0.1%). Of the sequences assigning to eukaryotes, ~5000 reads matched with commonly used DNA barcodes (12S, 16S, 18S, 28S, COI, cyt*b*). The overall low level of taxonomic assignment and the proportionally high number of hits to prokaryotes compared to eukaryotes using the ESS data is not surprising when considering the bias of the existing reference databases and relative abundance of these taxonomic groups in seawater. There are only 3339 eukaryotic, 72311 prokaryotic and 5646 viral genomes currently on GenBank (accessed 10 August 2016), many of which are sourced from terrestrial organisms. Further, while there is genetic information on more taxa through a growing database of commonly used barcode genes, these regions only represent a small fraction of an organism’s genome. Therefore, considering that the library preparation of eDNA for ESS includes all of the genetic material (i.e. entire genomes) from potentially all of the taxa that were present at Coral Bay, the likelihood of recovering DNA fragments mapping to barcode regions that have previously been sequenced and are publically available is low. The frequency of eukaryotic hits in the ESS data also reflects the relatively low abundance of micro-eukaryotes in our oceans, as there are estimated to be approximately 10000 viruses, 1000 bacteria, and 20 micro-eukaryotes in 1 mL of seawater^44^. Therefore, due to the higher number of bacteria that are sampled compared to micro-eukaryotes, and the higher number of prokaryotic versus eukaryotic genomes sequenced (72311 to 3339, respectively), the chance of mapping ESS data to bacterial genes is far greater. Finally, the recovery of eDNA from macroscopic eukaryotes (e.g. metazoans) is restricted to DNA contained in biological secretions, larvae or decaying cells, which is in much lower concentration than eDNA derived from prokaryotes and micro-eukaryotes^45^.

To critically evaluate the capacity of the ESS data to inform on the non-microbial fraction of the eDNA library, we focused on recovering fish DNA fragments rather than characterise the total diversity present in the library given their well-described taxonomy relative to other groups. The initial Blastn search using NCBI assigned 1.2% of the eukaryotic sequences to fish (class Actinopterygii, Chondrichthyes and Cyclostomata; ~875 reads of the original 22.3 M reads − 0.00004%), which is a poor relative representation. The majority of these fish reads mapped to genome assemblies, mRNA or phylogenetic informative genes. With regards to assigning taxonomic identity, the most valuable reads are instead those that map on to commonly used reference barcodes^46^. Searching the 22.3 million reads, only three sequences that are commonly used as DNA barcodes mapped to fish (Fig. 1); two 18S rDNA sequences, which could only resolve to the class Actinopterygii (matching six fishes with equal similarity; Bit Score = 248.348), and one 28S rDNA fragment with similar low-resolving power (Bit Score = 161.786). The low number of reads and lack of resolving power for fish in the ESS dataset showcases the limited ability of ESS data to inform on eukaryotic diversity.

Therefore, while shotgun sequencing using NGS platforms may represent the most unbiased way to explore eDNA from seawater, and has proven useful for the study of bacteria^47^, we demonstrate that the application of ESS for eukaryotes is currently not feasible and lacks resolution compared to metabarcoding (see below). Even for abundant and well-characterised marine taxa such as fish, NGS and ESS was unable to ‘cut through’ the microbial biomass that overwhelms the data that we recovered. While enrichment of target species through capture-probes is possible^39^, it is currently cost-prohibitive for use in routine monitoring of marine ecosystems due to the unwieldy number of libraries and probes that would be required for coverage across all taxa.

### Metabarcoding of eDNA from seawater

**Figure 2:**
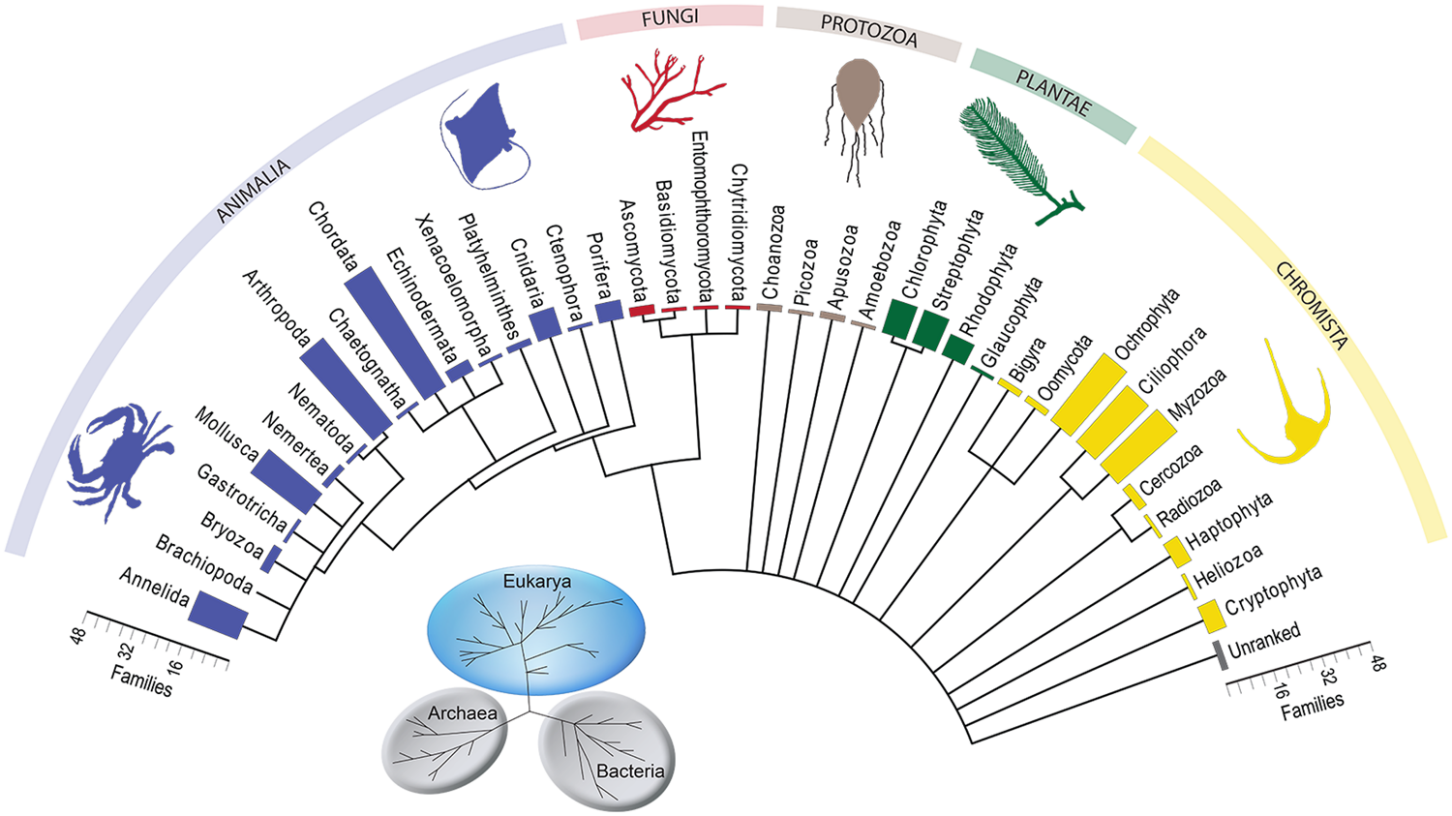
Taxonomic phylogram of eukaryotic diversity at Coral Bay in west Australia derived from ToL-metabarcoding. Bar graphs indicate the number of families in each phyla characterised at Coral Bay, and are coloured according to kingdom.

**Figure 3:**
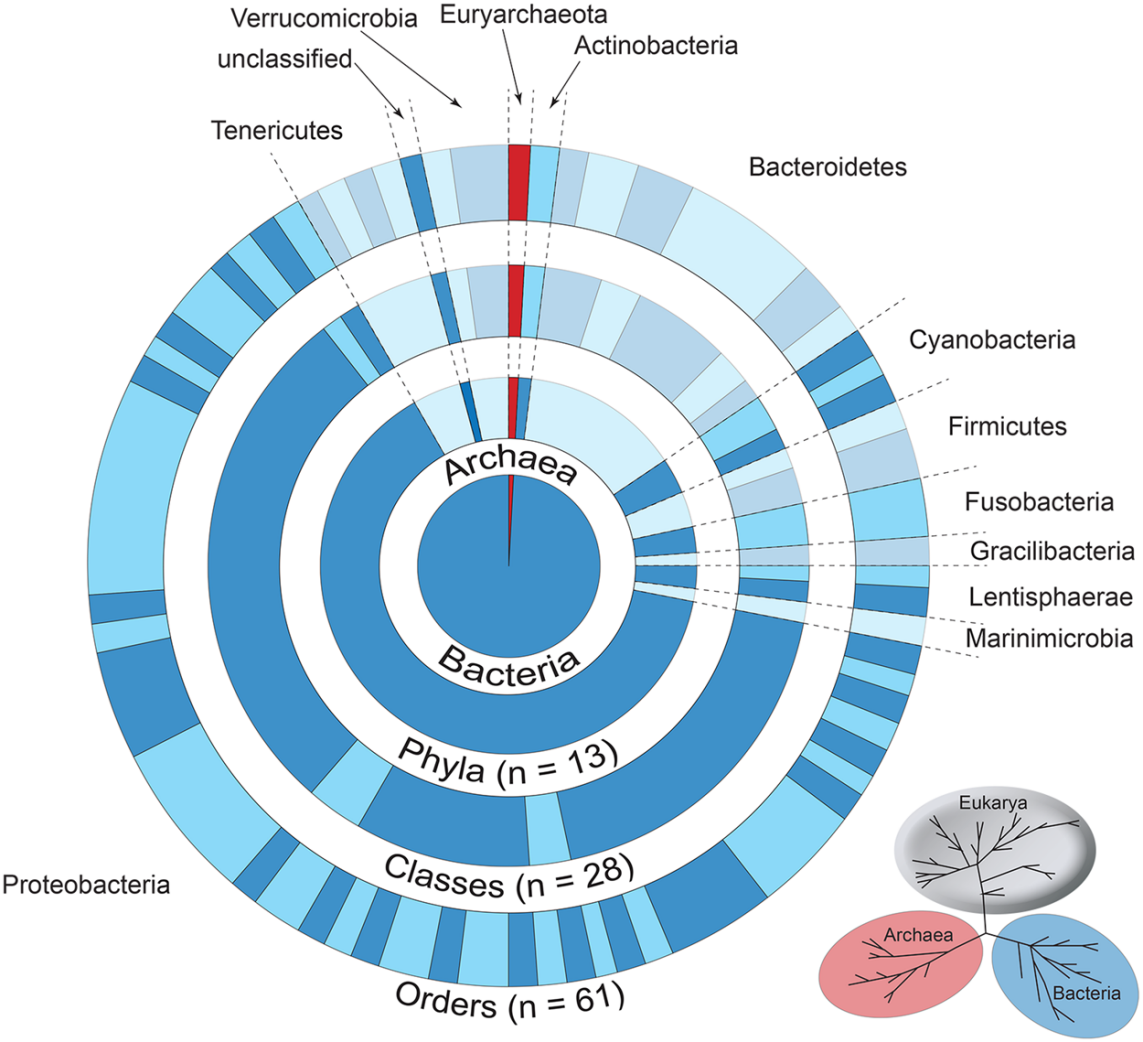
Hierarchical pie chart of prokaryotic diversity at Coral Bay in west Australia derived from ToL-metabarcoding. The inner pie chart represents the relative proportion of families identified in bacteria (blue) and archaea (red). Each segmented circle illustrates the number of taxa (phyla, classes and orders) characterised for both bacteria and archaea, and is scaled according to the number of families within each rank. Dotted lines partition the number of taxa within each phylum, which are named around the circumference of the chart.

The lack of non-microbial taxa detected from the shotgun sequencing data led us to explore the potential of ToL-metabarcoding, which we define as the use of multiple metabarcoding assays to survey a wide array of biotic diversity. Using ten different metabarcoding assays, a total of 1.2 million amplicon reads (that passed quality filtering) were used to generate a multi-taxon eDNA snapshot of the marine biodiversity at Coral Bay, 20-fold less sequencing effort than that used for ESS. Compared to the 14.1% of the ESS data that could be assigned to taxa, 79.7% of the metabarcoding sequence data could be assigned to taxa at Coral Bay (Supplementary Datas 1 and 2). The metabarcoding data was assigned to 434 eukaryotic taxa: 38 phyla, 88 classes, 186 orders and 287 families (Fig. 2; Table 1; Supplementary Data 3). Likewise for prokaryotes, 445 Operational Taxonomic Units (OTUs) from 14 phyla, 28 classes, 61 orders and 96 families were detected (Fig. 3; Supplementary Data 3). By using a suite of metabarcoding assays that target different organisms, all the major taxonomic lineages including the Animalia, Fungi, Protozoa, Plantae, Chromista, Bacteria, and Archaea were detected. For example, three classes of vertebrates were recovered from metabarcoding; Actinopterygii (ray-finned fishes), Chondrichthyes (cartilaginous fishes) and Mammalia. The majority of vertebrate diversity was within the class Actinopterygii (41 families), whereas whiptail stingrays (Dasyatidae) and eagle rays (Myliobatidae), as well as dolphins (Delphinidae), made up the families detected in the class Chondrichthyes and Mammalia, respectively. Thirty classes of invertebrates from 16 phyla, including arthropods and benthic organisms such as cnidarians (which includes corals), sponges and bivalves, were also detected from seawater samples. Interestingly, ten classes (31 families) of plants from four phyla were detected, including some of terrestrial origin, which were likely encountered via dispersal of pollen by wind and freshwater^48^. Micro-eukaryotes, such as fungi and phytoplankton, as well as prokaryotes, including dominant bacterioplankton such as SAR11, SAR 86, SAR116 and OM43^49^, were also present in the samples. Rather than critically evaluate the efficiency of each PCR assay used, what this study showcases is the capacity to audit the entire spectrum of taxonomic diversity present in a tropical marine environment using DNA samples extracted from seawater. A similar approach using six PCR assays on eDNA extracted from soil samples detected terrestrial prokaryotes and eukaroytes^50^, and recent studies on eDNA extracted from seawater and settlement plates detected a wide range of eukaryotes^51–54^. Collectively, these studies indicate that eDNA methodologies can be used on substrates from a variety of environments to assess a broad range of taxa. With further methodological development, ToL-metabarcoding provides compelling evidence for its inclusion into a biomonitoring ‘toolkit’ for marine environments.

**Table 1:** The number of eukaryotic taxa identified within each taxonomic rank at Coral Bay in west Australia via ToL-metabarcoding.

Kingdom	Phylum	Class	Order	Family	Genus	Species
Animalia	Annelida	2	7	17	16	
	Arthropoda	3	12	35	38	8
	Brachiopoda	1				
	Bryozoa	1	1	3		
	Chaetognatha		1	1	1	
	Chordata	5	17	45	73	36
	Cnidaria	2	5	9	7	
	Ctenophore	1	1	1	1	
	Echinodermata	3	2	3	4	2
	Gastrotricha		1	1		
	Mollusca	4	15	22	19	5
	Nematoda	1	1	1		
	Nemertea	2	1	2	2	
	Platyhelminthes	4	3	2		
	Porifera	3	6	6	6	
	Xenacoelomorpha	1		1		
Chromista	Bigyra	1	2	2	3	
	Cercozoa	4	4	3	4	
	Ciliophora	7	15	24		
	Cryptophyta	3	3	6	6	
	Haptophyta	4	5	5	4	1
	Heliozoa	1	1	1	1	
	Myzozoa	3	10	25	36	2
	Ochrophyta	9	31	27		
	Oomycota	1	3	2		
	Radiozoa	2	2	1	1	
Fungi	Ascomycota	3	4	3		
	Basidiomycota	2	2	1	1	
	Chytridiomycota	1	1	1	1	
	Entomophthoromycota	1	1	1	1	
Plantae	Chlorophyta	7	10	11	17	
	Glaucophyta	1		1	1	
	Rhodophyta	1	5	7	3	
	Streptophyta	1	11	11	2	
Protozoa	Amoebozoa		1	1	1	
	Apusozoa			2	3	
	Choanozoa	2	1	2	3	
	Picozoa	1	1	1		
**Total**	**38**	**88**	**186**	**287**	**255**	**54**

When comparing ESS to ToL-metabarcoding, it is clear that the latter method is superior in detecting fish taxa using eDNA. While only three barcode sequences could be assigned to the well-characterised Actinopterygii using ESS (and could not resolve to lower taxonomic levels), 69 Actinopterygii taxa were resolved using ToL-metabarcoding, including 33 to the species level. Further, the application of specific primers for the detection of fishes on eDNA collected from water indicates advantages over traditional survey methods. Analysis of eDNA recovered higher numbers of fish taxa in both marine and freshwater systems compared to traditional surveying methods^55,56^, and when comparing eDNA surveys to known fish diversity present in aquaria (e.g. Okinawa Churaumi Aquarium, 180 fish species), Miya *et al*.^34^ were able to identify >90% of taxa from their 12S rRNA sequences. In our data set, we detected small “bait fishes” from the genera *Atherinomorus*, *Engraulis*, *Hypoatherina*, *Hyporhamphus*, *Sardinella*, *Spratelloides* and *Strongylura*. While these genera are sometimes seen and recorded by other techniques such as Underwater Visual Census (UVC) by SCUBA divers or Baited Remote Underwater Video (BRUV) systems, it is extremely challenging to identify fish to species-level even if the family can be determined visually^7^. We also detected a number of cryptobenthic fishes with eDNA (e.g. Families Blenniidae, Gobiidae and Pseudochromidae) and fishes that are mostly active at night (e.g. families Apogonidae, Gerreidae and Holocentridae), which are usually not sampled adequately by other techniques^7,57,58^. Several species from families targeted by recreational and commercial fishers (i.e. Carangidae, Lethrinidae, Lutjanidae, Mugilidae, Epinephelidae) were also recovered from the metabarcoding data. As eDNA methodologies are non-invasive and show high sensitivity and detection capabilities, the application of metabarcoding eDNA for surveying fish in combination with assays that target other taxa are likely to be adopted in future monitoring programs.

### The importance and utility of multiple metabarcoding assays

**Figure 4:**
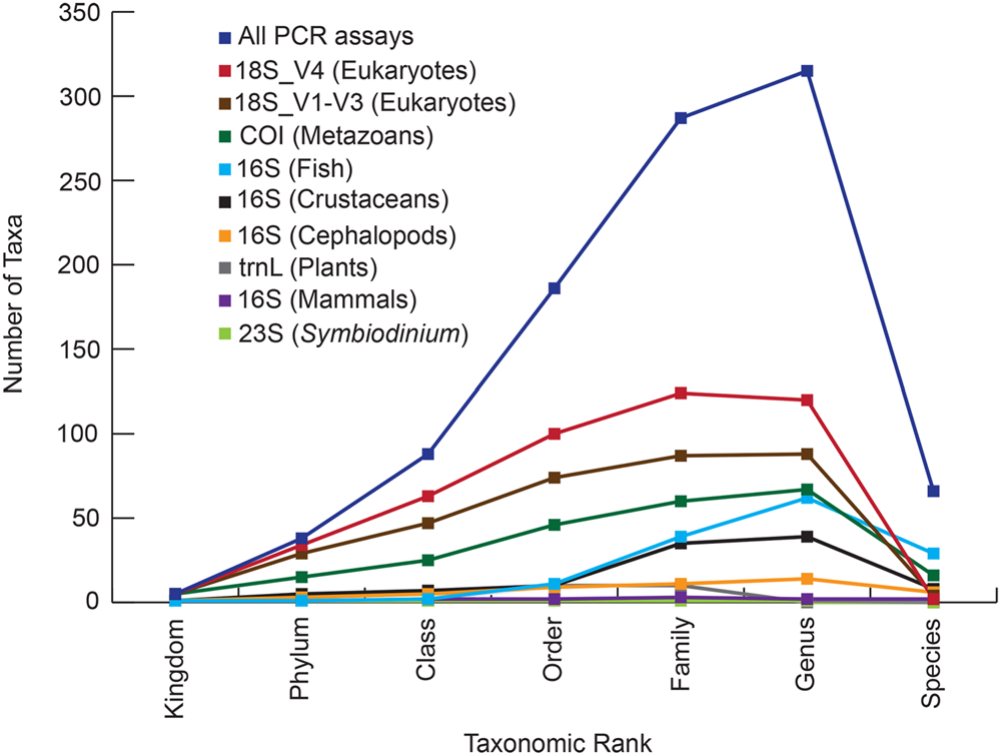
Line graph representing the number of eukaryotic taxa recorded at Coral Bay in west Australia using eDNA. Coloured lines indicate the number of taxa identified for each taxonomic rank for the nine PCR assays that target eukaryotes.

Our data advocates strongly for the use of ToL-metabarcoding as opposed to relying on a single ‘universal’ PCR assay to audit biota in the marine environment. The universal PCR assay based on the (commonly employed) 18S rDNA V4 region detected 191 taxa, which represents only 44% of the total number of taxa characterised from all PCR assays combined (*N* = 434; Fig. 4). Further, when normalising for sequencing depth, the trend in the number of taxa resolved for each assay at each taxonomic rank is consistent to that depicted in Fig. 4 (Supplementary Data 4), and there is a significant difference in the number of taxa identified between assays at each taxonomic rank (Kruskal-Wallis rank sum test, p < 0.05). Our study indicated that the 18S universal assays detected the greatest number of taxa followed by the COI assay, which is consistent with that reported by Kelly *et al*.^51^. While *in silico* analysis and testing of primer sets on synthetic blends is preferred when assessing the efficiency of PCR assays to detect taxa of choice^35^, it is unrealistic when the goal is to characterise all organisms present in an ecosystem. This challenge is particularly relevant within regions of unknown biodiversity with poor reference barcodes. However, it is nevertheless clear, that using a suite of universal PCR assays in combination with specific assays that achieve taxonomic saturation (Supplementary Data 5), a greater genetic diversity of taxa is uncovered.

There are a number of challenges that arise with interpreting data from single assays including primer bias, gene copy number, PCR or sequencing artefacts and/or contamination^35,59,60^. The application of numerous assays can overcome many of these issues through multiple detection hits for specific taxa and/or detecting a greater range of species. To showcase the importance of gene and primer choice, Table 2 shows the number of taxa within class Actinopterygii that were identified across all PCR assays employed in the study. Firstly, it can be seen that if a specific group of taxa is important for assessing diversity, in this case the Actinopterygii, taxon-specific assays (versus universal assays) reveal more taxa and much higher levels of diversity. For example, for all levels of taxonomy, the fish 16S assay detected more fish (61 genera) than any other primer set, as well as more genetic diversity as measured by the number of OTUs (Table 2). This highlights the fact that ‘universal’ assays, while excellent for providing a snapshot of biodiversity, are not able to adequately capture the depth of diversity within specific groups of taxa. This outcome is also influenced by the fact that nuclear rRNA genes (i.e. 18S) typically used as universal assays provide lower taxonomic resolution than mitochondrial rRNA genes (i.e. 16S)^61^. Further, while it would seem ideal to design a fish-specific assay based on the COI gene, which is the barcode of choice for vouchered fish specimens, the primer binding regions are more variable and therefore do not allow for the same specificity as the 16S and 12S regions of the mitochondrial genome^34,61,62^. Secondly, the use of multiple PCR assays can provide higher levels of confidence in the organisms detected when multiple hits to the same group are achieved (e.g. 14 shared fish genera across PCR assays; Table 2). If interested in specific taxa, like fish, then the application of multiple fish specific primers, like the 16S assay employed here in combination with the 12S assay used in Miya *et al*.^34^, would be preferred. A second example from our dataset is the recovery and characterisation of diversity within a single genus. Dinoflagellates belonging to the genus *Symbiodinium* are important photosynthetic symbionts that associate with a wide range of marine invertebrates, including corals, and represent the most abundant phytoplankton in tropical waters^63^. Indeed, a high diversity of taxa within this single genus is important for understanding host associations between plants and animals in our oceans^64^. While our universal assay (18S V4) recovered *Symbiodinium*, the application of a genus-specific assay (cp23S) further resolved seven of the nine major sub-generic phylogenetic lineages (clades) within the genus^65^ (Supplementary Data 3), which represents the cornerstone of investigating the potential for corals to adapt to climate change^63,66^. Taken together, these data demonstrate that multiple PCR assays with different target spectrums will collectively provide better recovery of taxa. As each barcode has advantages and disadvantages related to its resolution, taxonomic specificity and availability of reference sequences, the assay design needs to be study specific^61^.

**Table 2:** The number of fish taxa and OTUs (class Actinopterygii) identified at Coral Bay in west Australia across PCR assays used in the study.

Primer Set	Order	Family	Genus	Species	OTUs
18S_V4F	4	2			16
18S_VR					
18S_1 F	1				2
18S_400 R					
m1COIintF	6	12	15	9	16
jgHCO2198					
16Smam1	1	2	2	2	2
16Smam2					
16SF/D	10	38	61	29	143
16S2R-degenerate					
Multiple Hits	7	12	14	6	

The row ‘Multiple Hits’ refers to the number of taxa identified with more than one PCR assay. OTUs were generated using a 98% similarity cut-off.

### Exploring haplotype diversity with eDNA

**Figure 5:**
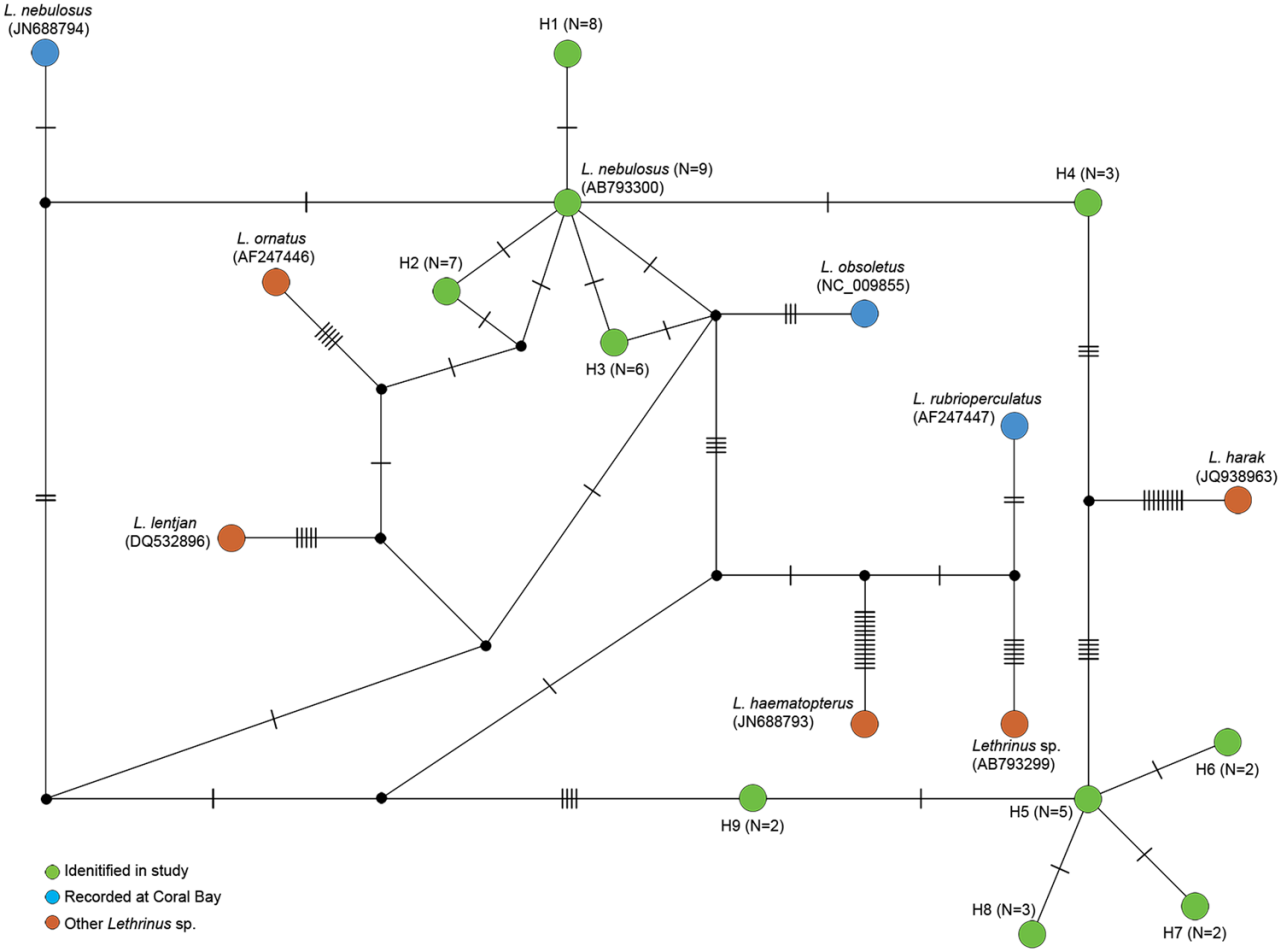
Network of *Lethrinus* 16S rDNA haplotypes. Green circles represent haplotypes identified at Coral Bay in west Australia in this study using eDNA, blue circles represent *Lethrinus* haplotypes obtained from NCBI for additional species recorded at Coral Bay, and red circles represent all other *Lethrinus* haplotypes available from NCBI. Numerals in brackets indicate the number of samples the haplotype was detected in (out of a total of 9). Genbank accession numbers are also indicated in brackets.

OTUs are commonly used to assess genetic diversity in a taxonomic independent approach that is free from the constraints of incomplete taxonomic frameworks and reference DNA databases^67,68^. OTU-based approaches are especially useful when comparing complex metabarcoding data from different locations and/or collection times, often via multivariate methods. Here we also investigated the potential of eDNA to inform on intra- and inter-species haplotype diversity beyond the OTU approach, and selected the fish genus *Lethrinus* as a model to explore this given the importance of species in this genus in recreational and commercial fisheries^43^. An error rate of 1.79% ± 1.59 was calculated for fish 16S amplicons (see methods), and was subsequently used to screen out low-frequency sequences from the metabarcoding data recovered from Coral Bay that assigned to *Lethrinus*. This approach is comparable to a recent study investigating whale shark population genetic diversity inferred from eDNA using a frequency cut-off of 1.3%^26^. After screening out low abundance sequences, and those not identified across more than one DNA sample, we detected ten reproducible *Lethrinus* haplotypes at Coral Bay (Fig. 5) that are unlikely to represent sequencing artefacts. One haplotype that we detected was identical to a reference barcode for *L. nebulosus* (GenBank Accession Number: AB793300), a species of *Lethrinus* present at Coral Bay based on the Atlas of Living Australia and complimentary observational surveys^69^. In addition, we detected four haplotypes that were 1 bp different to *L. nebulosus*. Considering that *L. nebulosus* has another reference haplotype 2 bp different (GenBank Accession Number: JN688794), we consider all five of these sequences to represent intra-species diversity for *L. nebulosus* at Coral Bay. A further five haplotypes that clustered together but were separate from other *Lethrinus* sequences in the network were identified in the sequence data. The Lethrinid species that these correlate to, however, is unknown, as there are no 16S reference barcodes from multiple species of *Lethrinus* known to occur in Coral Bay (e.g. *L. atkinsoni*, *L. genivittatus*, *L. laticaudis*, *L. olivaceus*, *L. variegatus*). That said, our haplotype data for *Lethrinus* showcases the capacity to extract and explore a particular species or genus within a more complex dataset (i.e. from all fish species that are co-amplified in a metabarcoding assay), as opposed to extracting haplotypes from a species-specific dataset, which was done for the whale shark study^26^. Collectively, these analyses demonstrate that when metabarcoding data is properly filtered, eDNA has the ability to extend beyond taxa lists and provide information on the genetic diversity of species across both time and space.

### Caveats and future directions

Our data show that ToL-metabarcoding can significantly advance the capacity to monitor tropical environments and implement EBM, but this approach, like those before it, does not represent an endpoint. For example, our study shows that ToL-metabarcoding performs better than ESS by providing more identifiable taxa across the tree of life in the face of an abundance of bacteria. Moreover, the use of multiple assays in ToL-metabarcoding revealed a significant fraction of taxa that would have been missed with a single (universal) assay. That said, despite filtering several one litre replicate samples in this study (*N* = 9), the sampling design did not enable a thorough investigation of inter-sample variation. We have therefore yet to explore how repeatable these assays are, or how effective they lend themselves to tracking changes in biodiversity across multiple spatial and temporal scales^25^. Rigorous testing can be achieved by reprocessing all of the samples from the beginning of the workflow to the end and then comparing results, sampling the same location at another time point or additional sampling at more distant sites, but that is beyond the scope of this study. Regardless of the NGS method used, there was always a significant fraction of the sequence data that could not be assigned a taxonomic rank. This deficiency reinforces the need for improved DNA reference databases, but also the awareness that tandemly running taxonomic-independent approaches (i.e. OTU analysis) provides more accurate measures of beta and alpha diversity. Finally, at present, ToL-metabarcoding can be expensive, labour intensive and difficult for more general ecologists to implement when compared to traditional biodiversity survey methods in the field, but this will only continue to decrease with the refinement of metabarcoding lab protocols and availability of commercial services. Moreover, no traditional survey method has yet been able to holistically capture the ecosystem composition across the entire tree of life in the way ToL-metabarcoding can.

## Conclusions

The goal of EBM is to consider biodiversity holistically, and in this regard, the taxa identified here using eDNA sourced from seawater is unparalleled in scope by any other survey method yet devised. Further, sampling seawater is easier than other methodologies currently used for assessing biodiversity, as it requires minimal equipment, is rapid, non-invasive, overcomes the need for deploying infrastructure and has the potential to be routinely collected using autonomous gliders or drones^16,18^. With present sequencing technologies and associated costs, we have shown that metabarcoding is superior to ESS in terms of representing ocean biodiversity and for increasing the recovery of non-microbial taxa. Multiple PCR assays should be employed however, that include universal primers, to provide biodiversity snapshots across a broad taxonomic spectrum, and taxon-specific primers, to focus on groups of interest. Once the collection and isolation of DNA from the environment, as well as laboratory and bioinformatics workflows are standardised, the application of eDNA analyses hold great promise for future marine biomonitoring^16,18^.

## Materials and Methods

### Sampling site, water collections and DNA extraction

Nine one litre seawater samples were collected using sterile Nalgene bottles from the surface at Coral Bay, which is located within the World Heritage site of Ningaloo Reef on the northwest coast of Australia. Three samples were collected on the 17^th^ of March 2015 at 9:00 am from Coral Bay jetty (−23.154793, 113.766328), and six samples were collected from within the lagoon (−23.154793, 113.766328); three at 5:30 pm on the 17^th^ of March and three at 6:30 am on the 18^th^ of March. Each one litre seawater sample was filtered across a Millipore 0.2 μm hydrophilic nylon membrane (Merck Millipore, Massachusetts, USA) using a Masterflex peristaltic pump (Cole Palmer, Vernon Hills, USA). The membrane disc containing captured eDNA and cellular material from the water column was immediately frozen and stored at −20 °C. Total nucleic acids were extracted directly from the filter membranes using the DNeasy Blood and Tissue Kit (Qiagen; Venlo, Netherlands) in a dedicated PCR-free DNA extraction laboratory, as well as a blank extraction control, and eluted in 100 μL AE buffer (Qiagen; Venlo, Netherlands).

### Shotgun sequencing and analyses

Equal volumes of each of the nine DNA extracts were pooled together and used to prepare a single shotgun library using the Nextera XT DNA library kit (Illumina, San Diego, USA). ESS was performed using a NextSeq® 500/550 v2 high output 300 cycle kit on an Illumina NextSeq system located in the Trace and Environmental DNA (TrEnD) Laboratory at Curtin University. Sequences with an average Q score ≤25 that contained no ambiguous nucleotides and were 151 base pairs (bp) in size (corresponding to the length of a DNA fragment sequenced uni-directionally), were compared to the National Center for Biotechnology Information (NCBI) nucleotide database using BLASTN on the Magnus Cray XC40 system located in the Pawsey Supercomputing Centre at Technology Park in west Australia. Assignment of sequences to taxa at a particular taxonomic level was assessed using the software MEGAN 5.11.3^70^ using a Min Score of 100 and a Top Percent of 10 under the LCA Parameters.

### PCR metabarcoding and sequencing

PCR was performed in duplicate on each of the nine DNA extracts using ten primer sets containing template-specific oligonucleotides (Supplementary Data 6) that target different taxonomic groups. To reduce the likelihood of cross-contamination, chimera production and index-tag switching^71^, amplification of target DNA was performed in a single round of PCR using fusion tag primers consisting of an Illumina adaptor, indexes unique to this study and the template specific oligonucleotide in an ultra-clean laboratory designed for ancient DNA work. PCR reagents included 1 × AmpliTaq Gold® Buffer (Life Technologies, Massachusetts, USA), 2 mM MgCl_2_, 0.25 μM dNTPs, 10 μg BSA, 5 pmol of each primer, 0.12 × SYBR® Green (Life Technologies), 1 Unit AmpliTaq Gold DNA polymerase (Life Technologies), 2 μl of DNA and Ultrapure™Distilled Water (Life Technologies) made up to 25 μl total volume. PCR was performed on a StepOnePlus Real-Time PCR System (Applied Biosystems, Massachusetts, USA) under the following conditions: initial denaturation at 95 °C for 5 min, followed by 40 cycles of 30 s at 95 °C, 30 s at the primer annealing temperature and 45 s at 72 °C, with a final extension for 10 min at 72 °C. Complete primer information and annealing temperatures are provided in Supplementary Data 6. For the three primer sets that target the nuclear 18S and mitochondrial COI genes, PCR was performed using three annealing temperatures in an attempt to maximise template amplification and the diversity of taxa detected. All duplicate PCR products from the same 1 L sample were combined prior to library pooling. PCR negative controls were included for all assays and any taxa detected within them were removed from all samples for analyses.

Libraries for sequencing were made by pooling amplicons into equimolar ratios based on qPCR Ct values and band intensity on a 2% agarose gel stained with ethidium bromide. Amplicons in each library were size-selected using a Pippin Prep (Sage Science, Beverly, USA) and purified using the Qiaquick PCR Purification Kit (Qiagen; Venlo, Netherlands). The volume of purified library added to the sequencing run was determined using qPCR against DNA standards of known molarity as in Murray *et al*.^72^. Depending on the amplicon size, libraries were either unidrectionally sequenced using a 300 cycle MiSeq® V2 Reagent Kit and nano flow cell, or with paired-end sequencing using a 500 cycle MiSeq® V2 Reagent Kit and standard flow cell on an Illumina MiSeq platform located in the TrEnD Laboratory at Curtin University. Sequence data is available from the Dryad Digital Repository: http://dx.doi.org/10.5061/dryad.qq11c.

### Metabarcoding analyses

All data generated by Illumina sequencing were filtered through a series of quality control steps prior to taxonomic assignment and OTU analyses. Metabarcoding reads recovered by paired-end sequencing were first stitched together using the Illumina MiSeq analysis software under the default settings. In order to eliminate low quality sequences, only reads matching 100% to Illumina adaptors, index barcodes and template specific oligonucleotides identified using Geneious® 8.1.4.^73^ were kept for downstream analyses. For each sample, Mothur 1.36.1^74^ was used to remove singletons, sequences that had an average Q score ≤25 and reads that contained ambiguous bases. Potential chimeras were identified using Perseus^75^ and removed from the dataset. Amplicons originating from eukaryotes that passed quality filtering were queried against the NCBI nucleotide database using BLASTN on the Magnus Cray XC40 system.

Given the lack of reference barcodes for most taxa, which limits the ability to asses inter-species diversity, we used a conservative approach to assign sequences to species, as opposed to percentage sequence similarity thresholds used in other studies^34,52^. Taxonomic identification was assigned to a species only if there was a 100% sequence identity match, if a sequence from at least one other species within the same genus was available for comparison (and <100% identical) and if the distribution of the species hit matched online database records for flora and fauna known to the region (e.g. Atlas of Living Australia; http://www.ala.org.au.). Otherwise, the taxonomic resolution achieved for a sequence was collapsed to the genus level or even higher. Taxonomic nomenclature was based on the World Register of Marine Species (WoRMS; http://www.marinespecies.org/). For prokaryotic 16S rDNA sequences, OTUs were identified following the MiSeq SOP outlined in Kozich *et al*.^76^ and using the NR SILVA database (release 123) accessed from the Mothur website (http://www.mothur.org/wiki/MiSeq_SOP) on the 2^nd^ of May, 2016. OTU classification for sequences assigned to the fish class Actinopterygii was also parsed using a 98% sequence similarity in Mothur 1.36.1^74^. Rarefaction analyses were performed using Analytic Rarefaction 1.3^77^ and plotted using R^78^.

### Network analyses

To assess haplotype diversity within the commercially targeted fish genus *Lethrinus*, a sequencing error rate for fish 16S rDNA was determined from single source tissue samples and thus used to filter out potential sequence artefacts. To achieve this, DNA extracted from 13 fish species, representing 12 different families (including two *Lethrinus* species), were individually amplified and the resulting 16S rDNA amplicons were sequenced as per above on an Illumina MiSeq. A single dominant haplotype for each species was considered the true representative haplotype for that species, and additional sequences were designated error generated during the PCR process, Illumina cluster formation and/or sequencing. The frequency of the highest erroneous sequence as a percentage of the true haplotype for each species was calculated, and an average error rate for fish 16S rDNA amplicons determined. This error rate was used to filter out low abundance sequences assigned to *Lethrinus* generated by PCR for the eDNA samples collected at Coral Bay when compared to the most abundant sequence assigned to *Lethrinus* in the same sample. In addition, potential *Lethrinus* 16S rDNA haplotypes that surpassed this error threshold needed to be present in multiple PCR libraries amplified from each of the seawater samples collected at Coral Bay. A network of *Lethrinus* 16S rDNA haplotypes recovered from Coral Bay and all available *Lethrinus* 16S sequences on Genbank was constructed in PopART (http://popart.otago.ac.nz). Because indels cannot be treated as a 5^th^ character state in PopART, the alignment was edited to make gaps informative. The Atlas of Living Australia was used to determine the feasibility of *Lethrinus* species that we recorded at Coral Bay.

## Electronic supplementary material

Supplementary Data
