# Supplementary material for: Ecosystem biomonitoring with eDNA: metabarcoding across the tree of life in a tropical marine environment
Source: Sci Rep. 2017 Sep 25;7:12240. doi: 10.1038/s41598-017-12501-5 (PMC5612959; doi:10.1038/s41598-017-12501-5)
Supplement: Supplementary file 1 — Supplementary Data [file 41598_2017_12501_MOESM1_ESM.pdf]

Ecosystem biomonitoring with eDNA: metabarcoding across the tree of life in a tropical marine environment

Michael Stat, Megan J. Huggett, Rachele Bernasconi, Joseph D. DiBattista, Tina Berry, Stephen J. Newman, Euan S Harvey, Michael Bunce

Supplementary Data 1

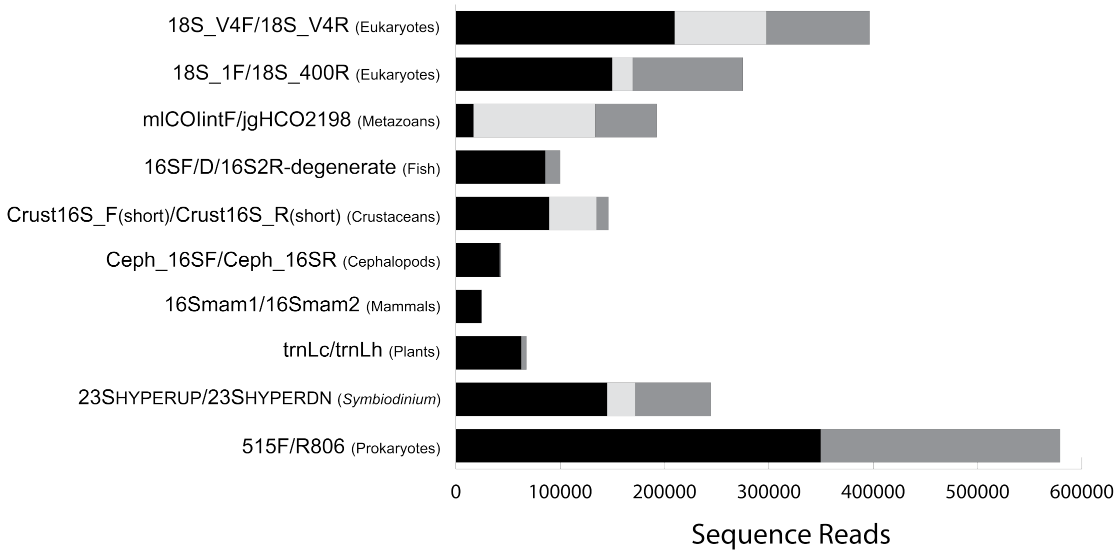

Number of sequence reads per primer set used in assigning taxa from replicate seawater samples collected at Coral Bay in West Australia using eDNA metabarcoding. Bar graphs indicate the total number of sequence reads recovered using each primer set. Within each bar, black segments represent the number of reads that passed quality filtering and could be assigned a taxon; the light shaded grey represents the number of reads that passed quality filtering but could not be assigned a taxon; and the dark shaded grey represents the number of sequences removed after quality filtering. The target taxa of each primer set is indicated in brackets following the primer name.

Supplementary Data 2

The mean number and standard deviation of sequences passing quality filtering, unique sequences, and taxa for the ten metabarcoding assays used in this study across the nine water samples collected at Coral Bay, West Australia.

**Assay**	**# Sequences ± SD**	**# Unique Sequences ± SD**	**# Taxa ± SD**
18S_V4 (Eukaryotes) [a]	12277 ± 3529	757 ± 147	53 ± 8
18S_V1-V3 (Eukaryotes) [b]	6230 ± 3202	477 ± 171	42 ± 5
COI (Metazoans) [c]	6457 ± 598	716 ± 61	33 ± 5
16S (Fish)	9507 ± 1869	301 ± 43	23 ± 4
16S (Crustaceans)	14998 ± 3096	221 ± 62	14 ± 5
16S (Cephalopods)	4652 ± 1639	63 ± 11	8 ± 4
trnL (Plants)	7901 ± 2224	182 ± 54	4 ± 1
16S (Mammals)	2702 ± 593	31 ± 9	3 ± 1
23S (*Symbiodinium*)	19118 ± 2512	382 ± 65	6 ± 1 (clades)
16S (Prokaryotes)	38843 ± 16208	2247 ± 726	195 ± 54 (OTUs)

[a] represents the sequences and taxa recovered when using an annealing temperature of 50° C.
[b] represents the sequences and taxa recovered when using an annealing temperature of 52° C.
[c] represents the sequences and taxa recovered when using an annealing temperature of 46° C.

Supplementary Data 3 - All eukaryotic taxa

Count	Kingdom	Phylum	Class	Order	Family	Genus	Species
1	Animalia	Annelida	Phascolosomatidea	Phascolosomatiformes	Phascolosomatidae	*Phascolosoma*	
2	Animalia	Annelida	Polychaeta	Capitellida	Capitellidae	*Dasybranchus*	
3	Animalia	Annelida	Polychaeta	Eunicida	Dorvilleidae	*Dorvillea*	
4	Animalia	Annelida	Polychaeta	Eunicida	Eunicidae	*Eunice*	
5	Animalia	Annelida	Polychaeta	Phyllodocida	Chrysopetalidae	*Chrysopetalum*	
6	Animalia	Annelida	Polychaeta	Phyllodocida	Hesionidae		
7	Animalia	Annelida	Polychaeta	Phyllodocida	Nereididae	*Ceratonereis*	
8	Animalia	Annelida	Polychaeta	Phyllodocida	Syllidae	*Branchiosyllis*	
9	Animalia	Annelida	Polychaeta	Phyllodocida	Syllidae	*Erinaceusyllis*	
10	Animalia	Annelida	Polychaeta	Phyllodocida	Syllidae	*Exogone*	
11	Animalia	Annelida	Polychaeta	Phyllodocida	Syllidae	*Salvatoria*	
12	Animalia	Annelida	Polychaeta	Sabellida	Fabriciidae	*Fabricinuda*	
13	Animalia	Annelida	Polychaeta	Sabellida	Fabriciidae	*Pseudofabriciola*	
14	Animalia	Annelida	Polychaeta	Sabellida	Sabellidae		
15	Animalia	Annelida	Polychaeta	Sabellida	Serpulidae		
16	Animalia	Annelida	Polychaeta	Spionida	Chaetopteridae	*Phyllochaetopterus*	
17	Animalia	Annelida	Polychaeta	Spionida	Spionidae	*Aurospio*	
18	Animalia	Annelida	Polychaeta	Spionida	Spionidae	*Spio*	
19	Animalia	Annelida	Polychaeta	Terebellida	Terebellidae		
20	Animalia	Annelida	Polychaeta		Opheliidae		
21	Animalia	Annelida	Polychaeta		Orbiniidae		
22	Animalia	Annelida	Polychaeta		Protodrilidae	*Protodrilus*	
23	Animalia	Arthropoda	Insecta	Blattodea			
24	Animalia	Arthropoda	Insecta	Coleoptera	Endomychidae		
25	Animalia	Arthropoda	Insecta	Hemiptera	Cicadellidae	*Opsius*	
26	Animalia	Arthropoda	Insecta	Hymenoptera	Formicidae	*Pheidole*	
27	Animalia	Arthropoda	Insecta	Orthoptera	Acrididae		
28	Animalia	Arthropoda	Malacostraca	Decapoda	Alpheidae	*Alpheus*	
29	Animalia	Arthropoda	Malacostraca	Decapoda	Axiidae	*Axiopsis*	
30	Animalia	Arthropoda	Malacostraca	Decapoda	Callianassidae		
31	Animalia	Arthropoda	Malacostraca	Decapoda	Diogenidae	*Calcinus*	*latens*
32	Animalia	Arthropoda	Malacostraca	Decapoda	Diogenidae	*Clibanarius*	
33	Animalia	Arthropoda	Malacostraca	Decapoda	Domeciidae		
34	Animalia	Arthropoda	Malacostraca	Decapoda	Dromiidae		
35	Animalia	Arthropoda	Malacostraca	Decapoda	Epialtinae		
36	Animalia	Arthropoda	Malacostraca	Decapoda	Galatheidae		
37	Animalia	Arthropoda	Malacostraca	Decapoda	Grapsidae	*Grapsus*	*albolineatus*
38	Animalia	Arthropoda	Malacostraca	Decapoda	Grapsidae	*Pachygrapsus*	*minutus*
39	Animalia	Arthropoda	Malacostraca	Decapoda	Hippolytidae	*Hippolyte*	
40	Animalia	Arthropoda	Malacostraca	Decapoda	Macrophthalmidae	*Macrophthalmus*	
41	Animalia	Arthropoda	Malacostraca	Decapoda	Majidae	*Micippa*	*platipes*
42	Animalia	Arthropoda	Malacostraca	Decapoda	Majidae	*Schizophrys*	
43	Animalia	Arthropoda	Malacostraca	Decapoda	Majidae	*Tiarinia*	
44	Animalia	Arthropoda	Malacostraca	Decapoda	Matutoidea	*Ashtoret*	
45	Animalia	Arthropoda	Malacostraca	Decapoda	Munididae		
46	Animalia	Arthropoda	Malacostraca	Decapoda	Palaemonidae	*Coralliocaris*	
47	Animalia	Arthropoda	Malacostraca	Decapoda	Penaeidae	*Melicertus*	
48	Animalia	Arthropoda	Malacostraca	Decapoda	Percnidae	*Percnon*	
49	Animalia	Arthropoda	Malacostraca	Decapoda	Pilumnidae		
50	Animalia	Arthropoda	Malacostraca	Decapoda	Portunidae	*Carupa*	
51	Animalia	Arthropoda	Malacostraca	Decapoda	Portunidae	*Charybdis*	
52	Animalia	Arthropoda	Malacostraca	Decapoda	Portunidae	*Libystes*	
53	Animalia	Arthropoda	Malacostraca	Decapoda	Portunidae	*Portunus*	*pelagicus*
54	Animalia	Arthropoda	Malacostraca	Decapoda	Portunidae	*Thalamita*	*admete*
55	Animalia	Arthropoda	Malacostraca	Decapoda	Tetraliidae	*Tetralia*	
56	Animalia	Arthropoda	Malacostraca	Decapoda	Trapeziidae	*Trapezia*	
57	Animalia	Arthropoda	Malacostraca	Decapoda	Varunidae		
58	Animalia	Arthropoda	Malacostraca	Decapoda	Xanthidae	*Atergatis*	
59	Animalia	Arthropoda	Malacostraca	Decapoda	Xanthidae	*Chlorodiella*	*laevissima*
60	Animalia	Arthropoda	Malacostraca	Decapoda	Xanthidae	*Cyclodius*	
61	Animalia	Arthropoda	Malacostraca	Decapoda	Xanthidae	*Etisus*	
62	Animalia	Arthropoda	Malacostraca	Decapoda	Xanthidae	*Liocarpilodes*	
63	Animalia	Arthropoda	Malacostraca	Decapoda	Xanthidae	*Liomera*	
64	Animalia	Arthropoda	Malacostraca	Decapoda	Xanthidae	*Macromedaeus*	
65	Animalia	Arthropoda	Malacostraca	Decapoda	Xanthidae	*Paraxanthias*	
66	Animalia	Arthropoda	Malacostraca	Decapoda	Xanthidae	*Pilodius*	*areolatus*
67	Animalia	Arthropoda	Malacostraca	Decapoda	Xanthidae	*Psaumis*	
68	Animalia	Arthropoda	Malacostraca	Stromatopoda	Nannosquillidae	*Pullosquilla*	
69	Animalia	Arthropoda	Maxillopoda	Calanoida	Acartiidae		
70	Animalia	Arthropoda	Maxillopoda	Cyclopoida			
71	Animalia	Arthropoda	Maxillopoda	Harpacticoida	Ameiridae		
72	Animalia	Arthropoda	Maxillopoda	Harpacticoida	Canuellidae		
73	Animalia	Arthropoda	Maxillopoda	Lithoglyptida	Lithoglyptidae	*Auritoglyptes*	
74	Animalia	Arthropoda	Maxillopoda	Poecilostomatoida	Catiniidae		
75	Animalia	Arthropoda	Maxillopoda	Poecilostomatoida	Lichomolgidae	*Lichomolgus*	

76	Animalia	Arthropoda	Maxillopoda	Poecilostomatoida	Pseudanthessiidae	*Pseudanthessius*	
77	Animalia	Brachiopoda	Rhynchonellata				
78	Animalia	Bryozoa	Gymnolaemata	Cheilostomata	Beaniidae		
79	Animalia	Bryozoa	Gymnolaemata	Cheilostomata	Bictectiporidae		
80	Animalia	Bryozoa	Gymnolaemata	Cheilostomata	Smittinidae		
81	Animalia	Chaetognatha		Biphragmophora	Spadellidae	*Spadella*	
82	Animalia	Chordata	Actinopterygii	Anguiliformes	Muraenidae	*Gymnothorax*	*pseudothyrsoideus*
83	Animalia	Chordata	Actinopterygii	Atheriniformes	Atherinidae	*Atherinomorus*	
84	Animalia	Chordata	Actinopterygii	Atheriniformes	Atherinidae	*Hypoatherina*	
85	Animalia	Chordata	Actinopterygii	Beloniformes	Belonidae	*Strongylura*	
86	Animalia	Chordata	Actinopterygii	Beloniformes	Belonidae	*Tylosurus*	*gavialoides*
87	Animalia	Chordata	Actinopterygii	Beloniformes	Hemiramphidae	*Hyporhamphus*	
88	Animalia	Chordata	Actinopterygii	Beryciformes	Holocentridae	*Sargocentron*	*rubrum*
89	Animalia	Chordata	Actinopterygii	Cluperiformes	Clupeidae	*Sardinella*	
90	Animalia	Chordata	Actinopterygii	Cluperiformes	Clupeidae	*Spratelloides*	
91	Animalia	Chordata	Actinopterygii	Cluperiformes	Engrulidae	*Engraulis*	
92	Animalia	Chordata	Actinopterygii	Mugiliformes	Mugilidae	*Mugil*	*cephalus*
93	Animalia	Chordata	Actinopterygii	Perciformes	Acanthuridae	*Acanthurus*	*triostegus*
94	Animalia	Chordata	Actinopterygii	Perciformes	Apogonidae	*Apogon*	
95	Animalia	Chordata	Actinopterygii	Perciformes	Blenniidae	*Salarias*	
96	Animalia	Chordata	Actinopterygii	Perciformes	Carangidae	*Alectis*	
97	Animalia	Chordata	Actinopterygii	Perciformes	Carangidae	*Caranx*	
98	Animalia	Chordata	Actinopterygii	Perciformes	Carangidae	*Gnathanodon*	*speciosus*
99	Animalia	Chordata	Actinopterygii	Perciformes	Carangidae	*Scomberoides*	*commersonnianus*
100	Animalia	Chordata	Actinopterygii	Perciformes	Carangidae	*Trachinotus*	*blochii*
101	Animalia	Chordata	Actinopterygii	Perciformes	Chaetodontidae	*Chaetodon*	
102	Animalia	Chordata	Actinopterygii	Perciformes	Gerreidae	*Gerres*	
103	Animalia	Chordata	Actinopterygii	Perciformes	Gobiidae	*Asterropteryx*	
104	Animalia	Chordata	Actinopterygii	Perciformes	Gobiidae	*Bathygobius*	
105	Animalia	Chordata	Actinopterygii	Perciformes	Gobiidae	*Gnatholepis*	
106	Animalia	Chordata	Actinopterygii	Perciformes	Gobiidae	*Gobiodon*	*axillaris*
107	Animalia	Chordata	Actinopterygii	Perciformes	Haemulidae	*Plectorhinchus*	
108	Animalia	Chordata	Actinopterygii	Perciformes	Kyphosidae	*Kyphosus*	*bigibbus*
109	Animalia	Chordata	Actinopterygii	Perciformes	Labridae	*Anampses*	
110	Animalia	Chordata	Actinopterygii	Perciformes	Labridae	*Cheilinus*	*trilobatus*
111	Animalia	Chordata	Actinopterygii	Perciformes	Labridae	*Cheilio*	*inermis*
112	Animalia	Chordata	Actinopterygii	Perciformes	Labridae	*Chlorurus*	*sordidus*
113	Animalia	Chordata	Actinopterygii	Perciformes	Labridae	*Coris*	*aygula*
114	Animalia	Chordata	Actinopterygii	Perciformes	Labridae	*Cymolutes*	
115	Animalia	Chordata	Actinopterygii	Perciformes	Labridae	*Halichoeres*	*nebulosus*
116	Animalia	Chordata	Actinopterygii	Perciformes	Labridae	*Hemigymnus*	*melapterus*
117	Animalia	Chordata	Actinopterygii	Perciformes	Labridae	*Labrichthys*	
118	Animalia	Chordata	Actinopterygii	Perciformes	Labridae	*Labroides*	*dimidiatus*
119	Animalia	Chordata	Actinopterygii	Perciformes	Labridae	*Stethojulis*	
120	Animalia	Chordata	Actinopterygii	Perciformes	Labridae	*Thalassoma*	*lunare*
121	Animalia	Chordata	Actinopterygii	Perciformes	Lethrinidae	*Lethrinus*	*nebulosus*
122	Animalia	Chordata	Actinopterygii	Perciformes	Lutjanidae	*Lutjanus*	*fulviflamma*
123	Animalia	Chordata	Actinopterygii	Perciformes	Microdesmidae	*Gunnellichthys*	
124	Animalia	Chordata	Actinopterygii	Perciformes	Mullidae	*Parupeneus*	
125	Animalia	Chordata	Actinopterygii	Perciformes	Pinguipedidae		
126	Animalia	Chordata	Actinopterygii	Perciformes	Pomacentridae	*Abudefduf*	*vaigiensis*
127	Animalia	Chordata	Actinopterygii	Perciformes	Pomacentridae	*Cheiloprion*	*labiatus*
128	Animalia	Chordata	Actinopterygii	Perciformes	Pomacentridae	*Chromis*	*viridis*
129	Animalia	Chordata	Actinopterygii	Perciformes	Pomacentridae	*Dascyllus*	*aruanus*
130	Animalia	Chordata	Actinopterygii	Perciformes	Pomacentridae	*Pomacentrus*	*coelestis*
131	Animalia	Chordata	Actinopterygii	Perciformes	Pomacentridae	*Pomacentrus*	*moluccensis*
132	Animalia	Chordata	Actinopterygii	Perciformes	Pomacentridae	*Stegastes*	*nigricans*
133	Animalia	Chordata	Actinopterygii	Perciformes	Priacanthidae	*Heteropriacanthus*	
134	Animalia	Chordata	Actinopterygii	Perciformes	Pseudochromidae	*Psedodochromis*	
135	Animalia	Chordata	Actinopterygii	Perciformes	Labridae	*Hipposcarus*	
136	Animalia	Chordata	Actinopterygii	Perciformes	Labridae	*Scarus*	*schlegeli*
137	Animalia	Chordata	Actinopterygii	Perciformes	Scatophagidae	*Selenotoca*	
138	Animalia	Chordata	Actinopterygii	Perciformes	Serranidae	*Epinephelus*	*quoyanus*
139	Animalia	Chordata	Actinopterygii	Perciformes	Siganidae	*Siganus*	
140	Animalia	Chordata	Actinopterygii	Perciformes	Sillaginidae	*Sillago*	
141	Animalia	Chordata	Actinopterygii	Perciformes	Sphyraenidae	*Sphyraena*	
142	Animalia	Chordata	Actinopterygii	Scorpaeniformes	Sebastidae		
143	Animalia	Chordata	Actinopterygii	Siluriformes			
144	Animalia	Chordata	Actinopterygii	Syngnathiformes	Fistularidae	*Fistularia*	
145	Animalia	Chordata	Actinopterygii	Tetraodontiformes	Balistidae	*Rhinecanthus*	*aculeatus*
146	Animalia	Chordata	Actinopterygii	Tetraodontiformes	Diodontidae	*Diodon*	*hystrix*
147	Animalia	Chordata	Actinopterygii	Tetraodontiformes	Monacanthidae	*Pervagor*	
148	Animalia	Chordata	Actinopterygii	Tetraodontiformes	Ostraciidae	*Ostracion*	*cubicus*
149	Animalia	Chordata	Actinopterygii	Tetraodontiformes	Tetraodontidae	*Arothron*	*hispidis*
150	Animalia	Chordata	Ascidiaceae	Enterogona	Ascidiidae	*Ascidia*	
151	Animalia	Chordata	Ascidiaceae	Pleurogona	Stylelidae	*Botryllus*	
152	Animalia	Chordata	Ascidiaceae	Stolidobranchia	Pyuridae	*Pyura*	

153	Animalia	Chordata	Chondrichthyes	Myliobatiformes	Dasyatidae	*Himantura*	*leoparda*
154	Animalia	Chordata	Chondrichthyes	Myliobatiformes	Dasyatidae	*Neotrygon*	*ningalooensis*
155	Animalia	Chordata	Chondrichthyes	Myliobatiformes	Dasyatidae	*Pastinachus*	*atrus*
156	Animalia	Chordata	Chondrichthyes	Myliobatiformes	Dasyatidae	*Taeniura*	
157	Animalia	Chordata	Chondrichthyes	Myliobatiformes	Myliobatidae	*Aetobatus*	
	Animalia	Chordata	Mammalia	Artiodactyla	Bovidae	*Bos*	
	Animalia	Chordata	Mammalia	Artiodactyla	Suidae	*Sus*	*scrofa*
158	Animalia	Chordata	Mammalia	Cetacea	Delphinidae		
	Animalia	Chordata	Mammalia	Primates	Hominidae	*Homo*	*sapiens*
159	Animalia	Chordata	Ophiuroidea	Ophiurida			
160	Animalia	Cnidaria	Anthozoa	Actinaria			
161	Animalia	Cnidaria	Anthozoa	Alcyonacea	Alcyoniidae		
162	Animalia	Cnidaria	Anthozoa	Scleractinia	Acroporidae	*Montipora*	
163	Animalia	Cnidaria	Anthozoa	Scleractinia	Pocilloporidae	*Pocillopora*	
164	Animalia	Cnidaria	Hydrozoa	Leptothecata	Campanulariidae	*Campanularia*	
165	Animalia	Cnidaria	Hydrozoa	Leptothecata	Campanulariidae	*Clytia*	
166	Animalia	Cnidaria	Hydrozoa	Leptothecata	Haleciidae	*Halecium*	
167	Animalia	Cnidaria	Hydrozoa	Leptothecata	Mitrocomidae	*Mitrocomella*	
168	Animalia	Cnidaria	Hydrozoa	Leptothecata	Plumulariidae	*Plumularia*	
169	Animalia	Cnidaria	Hydrozoa	Leptothecata	Sertulariidae		
170	Animalia	Cnidaria		Bivalvulida	Myxidiiadae		
171	Animalia	Ctenophora	Tentaculata	Platyctenida	Coeloplanidae	*Vallicula*	
172	Animalia	Echinodermata	Echinoidea				
173	Animalia	Echinodermata	Holothuroidea	Aspidochirotida	Holothuridae	*Holothuria*	*atra*
174	Animalia	Echinodermata	Ophiuroidea	Ophiurida	Amphiuridae	*Amphipholis*	
175	Animalia	Echinodermata	Ophiuroidea	Ophiurida	Ophiocomidae	*Ophiocoma*	
176	Animalia	Echinodermata	Ophiuroidea	Ophiurida	Ophiothrichidae	*Macrophiothrix*	*caenosa*
177	Animalia	Gastrotricha		Chaetonotida	Chaetonotidae		
178	Animalia	Mollusca	Bivalvia	Limoida	Limidae	*Limaria*	
179	Animalia	Mollusca	Bivalvia	Lucinoida	Lucinidae		
180	Animalia	Mollusca	Bivalvia	Mytiloida	Mytilidae		
181	Animalia	Mollusca	Bivalvia	Ostreoida	Ostreidae	*Lopha*	
182	Animalia	Mollusca	Bivalvia	Pterioida	Isognomonidae	*Isognomon*	
183	Animalia	Mollusca	Bivalvia	Solemyida	Solemyidae	*Solemya*	
184	Animalia	Mollusca	Bivalvia	Veneroida	Cardiidae	*Fragum*	
185	Animalia	Mollusca	Bivalvia	Veneroida	Lucinidae		
186	Animalia	Mollusca	Bivalvia	Veneroida	Petricolidae		
187	Animalia	Mollusca	Cephalopoda	Octopoda	Octopodidae	*Abdopus*	
188	Animalia	Mollusca	Cephalopoda	Octopoda	Octopodidae	*Octopus*	*cyanea*
189	Animalia	Mollusca	Cephalopoda	Octopoda	Octopodidae	*Octopus*	
190	Animalia	Mollusca	Cephalopoda	Teuthida	Loliginidae	*Sepioteuthis*	
191	Animalia	Mollusca	Gastropoda	Caenogastropoda	Cerithiidae	*Cerithium*	*atromarginatum*
192	Animalia	Mollusca	Gastropoda	Caenogastropoda	Cerithiidae	*Cerithium*	*rostratum*
193	Animalia	Mollusca	Gastropoda	Caenogastropoda	Cerithiidae	*Clypeomorus*	
194	Animalia	Mollusca	Gastropoda	Caenogastropoda	Litiopidae	*Alaba*	
195	Animalia	Mollusca	Gastropoda	Caenogastropoda	Turritellidae		
196	Animalia	Mollusca	Gastropoda	Cerithimorpha	Cerithidae	*Cerithium*	
197	Animalia	Mollusca	Gastropoda	Hypsogastropoda	Strombidae	*Strombus*	
198	Animalia	Mollusca	Gastropoda	Nudipleura			
199	Animalia	Mollusca	Gastropoda	Vetigastropoda	Haliotidae	*Haliotis*	*asinina*
200	Animalia	Mollusca	Gastropoda	Vetigastropoda	Trochidae	*Pseudostomatella*	
201	Animalia	Mollusca	Gastropoda	Vetigastropoda	Trochidae	*Rossiteria*	
202	Animalia	Mollusca	Gastropoda	Vetigastropoda	Trochidae	*Stomatella*	*impertusa*
203	Animalia	Mollusca	Gastropoda	Vetigastropoda	Trochidae	*Stomatia*	
204	Animalia	Mollusca	Gastropoda	Vetigastropoda	Turbinidae	*Turbo (Marmarostoma)*	
205	Animalia	Mollusca	Gastropoda		Littorinidae	*Echinolittorina*	
206	Animalia	Mollusca	Gastropoda		Strombidae	*Lambis*	
207	Animalia	Mollusca	Polyplacophora	Neoloricata	Mopaliidae		
208	Animalia	Nematoda	Chromadorea	Chromadorida	Chromadoridae		
209	Animalia	Nemertea	Enopla	Monostilifera	Tetrastemmatidae	*Tetrastemma*	
210	Animalia	Nemertea	Palaeonemertea		Cephalothricidae	*Cephalothrix*	
211	Animalia	Platyhelminthes	Monogenea				
212	Animalia	Platyhelminthes	Rhabditophora	Polycladida			
213	Animalia	Platyhelminthes	Trematoda				
214	Animalia	Platyhelminthes	Turbellaria	Macrostomida	Macrostomidae		
215	Animalia	Platyhelminthes	Turbellaria	Macrostomida	Microstomidae		
216	Animalia	Platyhelminthes	Turbellaria	Rhabdocoela			
217	Animalia	Porifera	Calcarea				
218	Animalia	Porifera	Demospongiae	Dictyoceratida			
219	Animalia	Porifera	Demospongiae	Hadromerida	Clionaidae	*Pione*	
220	Animalia	Porifera	Demospongiae	Halichondrida	Halichondriidae	*Halichondria*	
221	Animalia	Porifera	Demospongiae	Halichondrida	Halichondriidae	*Hymeniacidon*	
222	Animalia	Porifera	Demospongiae	Haplosclerida			
223	Animalia	Porifera	Demospongiae	Poecilosclerida	Desmacellidae	*Biemna*	
224	Animalia	Porifera	Demospongiae	Poecilosclerida	Microcionidae	*Clathria*	
225	Animalia	Porifera	Homoscleromorpha	Homosclerophorida	Plakinidae		
226	Animalia	Porifera		Homosclerophorida	Oscarellidae	*Oscarella*	

227	Animalia	Xenacoelomorpha	Acoela		Isodiametridae		
228	Chromista	Bigyra	Labyrinthulomycetes	Labyrinthulomycetes	Thraustochytriaceae	*Aplanochytrium*	
229	Chromista	Bigyra		Bicosoecida	Bicosoecidae	*Bicosoeca*	
230	Chromista	Bigyra		Bicosoecida		*Ceacitellus*	
231	Chromista	Cercozoa	Chlorarachnea	Chlorachniida	Chlorarachniaceae	*Bigelowiella*	
232	Chromista	Cercozoa	Imbricatea	Thaumatomanadida	Thaumatomastigidae		
233	Chromista	Cercozoa	Sarcomonadea	Cercomonadida	Cercomonadidae	*Massisteria*	
234	Chromista	Cercozoa	Thecofilosea	Cryomonadida		*Cryothecomonas*	
235	Chromista	Cercozoa				*Minorisa*	
236	Chromista	Ciliophora	Heterotrichea	Heterotrichida	Condylostomatidae	*Condylostoma*	
237	Chromista	Ciliophora	Litostomatea	Haptorida	Lacrymariidae	*Lacrymaria*	
238	Chromista	Ciliophora	Litostomatea	Pleurostomatida	Litonotidae	*Litonotus*	
239	Chromista	Ciliophora	Nassophorea	Synhymeniida	Orthodonellidae	*Zosterodasys*	
240	Chromista	Ciliophora	Oligohymenophorea	Hymenostomatida			
241	Chromista	Ciliophora	Oligohymenophorea	Peniculida			
242	Chromista	Ciliophora	Oligohymenophorea	Philasterida	Cinetochilidae	*Cinetochilum*	
243	Chromista	Ciliophora	Oligohymenophorea	Philasterida	Cohnilembidae	*Cohnilembus*	
244	Chromista	Ciliophora	Oligohymenophorea	Philasterida	Cohnilembidae	*Porpostoma*	
245	Chromista	Ciliophora	Oligohymenophorea	Philasterida	Loxocephalidae	*Cardiostomatella*	
246	Chromista	Ciliophora	Oligohymenophorea	Philasterida	Orchitophryidae	*Metanophrys*	
247	Chromista	Ciliophora	Oligohymenophorea	Philasterida	Orchitophryidae	*Paranophrys*	
248	Chromista	Ciliophora	Oligohymenophorea	Philasterida	Philasteridae		
249	Chromista	Ciliophora	Oligohymenophorea	Philasterida	Uronematidae	*Uronema*	*heteromarinum*
250	Chromista	Ciliophora	Oligohymenophorea	Philasterida	Uronematidae	*Uronemella*	
251	Chromista	Ciliophora	Oligohymenophorea	Pleuronematida	Cyclidiidae	*Cyclidium*	
252	Chromista	Ciliophora	Oligohymenophorea	Pleuronematida	Pleurodematidae	*Pleuronema*	*coronatum*
253	Chromista	Ciliophora	Phyllopharyngea	Dysteriida	Dysteriidae	*Dysteria*	
254	Chromista	Ciliophora	Prostomatea	Prorodontida	Colepidae	*Tiarina*	
255	Chromista	Ciliophora	Prostomatea	Prorodontida	Urotrichidae	*Urotricha*	
256	Chromista	Ciliophora	Spirotrichea	Choreotrichida	Strobilidiidae	*Pelagostrobilidium*	
257	Chromista	Ciliophora	Spirotrichea	Choreotrichida	Strobilidiidae	*Rimostrombidium*	
258	Chromista	Ciliophora	Spirotrichea	Choreotrichida	Strombidinopsidae	*Parastrombidinopsis*	
259	Chromista	Ciliophora	Spirotrichea	Choreotrichida	Strombidinopsidae	*Strombidinopsis*	
260	Chromista	Ciliophora	Spirotrichea	Euplotida	Uronychiidae	*Diophrys*	
261	Chromista	Ciliophora	Spirotrichea	Sporadotrichida	Oxytrichidae	*Hemigastrostyla*	
262	Chromista	Ciliophora	Spirotrichea	Tintinnida	Tintinnidae		
263	Chromista	Ciliophora	Spirotrichea	Urostylida	Holostichidae	*Holosticha*	*diademata*
264	Chromista	Ciliophora	Spirotrichea		Cyrtostrombidiidae	*Cyrtostrombidium*	
265	Chromista	Ciliophora	Spirotrichea		Strombidiidae	*Strombidium*	
266	Chromista	Ciliophora	Spirotrichea		Totoniidae	*Pseudotontonia*	
267	Chromista	Ciliophora	Spirotrichea			*Protocruzia*	
268	Chromista	Cryptophyta	Cryptophyceae	Cryptomonadales	Cryptomonadaceae		
269	Chromista	Cryptophyta	Cryptophyceae	Cryptomonadales	Goniomonadaceae	*Goniomonas*	
270	Chromista	Cryptophyta	Cryptophyceae	Cryptomonadales	Hemiselmidaceae	*Hemiselmis*	
271	Chromista	Cryptophyta	Cryptophyceae	Pyrenomonadales	Geminigeraceae	*Proteomonas*	
272	Chromista	Cryptophyta	Cryptophyceae	Pyrenomonadales	Geminigeraceae	*Teleaulax*	
273	Chromista	Cryptophyta	Cryptophyceae	Pyrenomonadales	Pyrenomonadaceae	*Rhodomonas*	
274	Chromista	Cryptophyta	Katablepharidophyta		Katablepharidaceae		
275	Chromista	Cryptophyta	Telonemea	Telonemida		*Telonema*	
276	Chromista	Haptophyta	Chrysophyceae	Chromulinales	Chromulinaceae		
277	Chromista	Haptophyta	Coccolithophyceae	Phaeocystales	Phaeocystaceae	*Phaeocystis*	
278	Chromista	Haptophyta	Coccolithophyceae	Prymnesiales	Chrysochromulinaceae	*Chrysochromulina*	
279	Chromista	Haptophyta	Pavlovophycidae	Pavlovales	Pavlovaceae	*Pavlova*	*pinguis*
280	Chromista	Haptophyta	Prymnesiophyceae	Isochrysidales	Noelaerhabdaceae	*Emiliania*	
281	Chromista	Heliozoa	Centrohelea	Centrohelida	Acanthocystidae	*Raineriophrys*	
282	Chromista	Myzozoa	Dinophyceae	Gonyaulacales	Amphidomataceae	*Amphidoma*	
283	Chromista	Myzozoa	Dinophyceae	Gonyaulacales	Amphidomataceae	*Azadinium*	
284	Chromista	Myzozoa	Dinophyceae	Gonyaulacales	Ceratiaceae	*Neoceratium*	
285	Chromista	Myzozoa	Dinophyceae	Gonyaulacales	Gonyaulacaceae	*Alexandrium*	
286	Chromista	Myzozoa	Dinophyceae	Gonyaulacales	Gonyaulacaceae	*Gonyaulax*	
287	Chromista	Myzozoa	Dinophyceae	Gymnodiniales	Brachidiniaceae	*Brachidinium*	
288	Chromista	Myzozoa	Dinophyceae	Gymnodiniales	Gymnodiniaceae	*Amphidinium*	*klebsii*
289	Chromista	Myzozoa	Dinophyceae	Gymnodiniales	Gymnodiniaceae	*Cochlodinium*	
290	Chromista	Myzozoa	Dinophyceae	Gymnodiniales	Gymnodiniaceae	*Gymnodinium*	
291	Chromista	Myzozoa	Dinophyceae	Gymnodiniales	Gymnodiniaceae	*Gyrodinium*	
292	Chromista	Myzozoa	Dinophyceae	Gymnodiniales	Gymnodiniaceae	*Lepidodinium*	
293	Chromista	Myzozoa	Dinophyceae	Gymnodiniales	Gymnodiniaceae	*Paragymnodinium*	
294	Chromista	Myzozoa	Dinophyceae	Gymnodiniales	Kareniaceae	*Karenia*	
295	Chromista	Myzozoa	Dinophyceae	Gymnodiniales	Kareniaceae	*Karlodinium*	
296	Chromista	Myzozoa	Dinophyceae	Gymnodiniales	Warnowiaceae	*Warnowia*	
297	Chromista	Myzozoa	Dinophyceae	Lophodiniales	Lophodiniaceae	*Woloszynskia*	
298	Chromista	Myzozoa	Dinophyceae	Peridiniales	Glenodiniaceae	*Glenodinium*	
299	Chromista	Myzozoa	Dinophyceae	Peridiniales	Heterocapsaceae	*Heterocapsa*	
300	Chromista	Myzozoa	Dinophyceae	Peridiniales	Peridiniaceae	*Pentapharsodinium*	
301	Chromista	Myzozoa	Dinophyceae	Peridiniales	Peridiniaceae	*Peridinium*	
302	Chromista	Myzozoa	Dinophyceae	Peridiniales	Pfiesteriaceae		
303	Chromista	Myzozoa	Dinophyceae	Peridiniales	Podolampaceae	*Blepharocysta*	

304	Chromista	Myzozoa	Dinophyceae	Peridiniales	Podolampaceae	*Podolampas*	
305	Chromista	Myzozoa	Dinophyceae	Peridiniales	Protoperidiniaceae	*Protoperidinium*	
306	Chromista	Myzozoa	Dinophyceae	Peridiniales	Thoracosphaeraceae	*Scrippsiella*	
307	Chromista	Myzozoa	Dinophyceae	Peridiniales		*Galeidinium*	
308	Chromista	Myzozoa	Dinophyceae	Prorocentrales	Prorocentraceae	*Exuviaella*	
309	Chromista	Myzozoa	Dinophyceae	Prorocentrales	Prorocentraceae	*Prorocentrum*	
310	Chromista	Myzozoa	Dinophyceae	Pyrocystales	Pyrocystaceae	*Dissodinium*	
311	Chromista	Myzozoa	Dinophyceae	Suessiales	Symbiodiniaceae	*Symbiodinium*	clade A
312	Chromista	Myzozoa	Dinophyceae	Suessiales	Symbiodiniaceae	*Symbiodinium*	clade B
313	Chromista	Myzozoa	Dinophyceae	Suessiales	Symbiodiniaceae	*Symbiodinium*	clade C
314	Chromista	Myzozoa	Dinophyceae	Suessiales	Symbiodiniaceae	*Symbiodinium*	clade D
315	Chromista	Myzozoa	Dinophyceae	Suessiales	Symbiodiniaceae	*Symbiodinium*	clade F
316	Chromista	Myzozoa	Dinophyceae	Suessiales	Symbiodiniaceae	*Symbiodinium*	clade G
317	Chromista	Myzozoa	Dinophyceae	Suessiales	Symbiodiniaceae	*Symbiodinium*	clade H
318	Chromista	Myzozoa	Dinophyceae	Syndiniales	Amoebophryaceae	*Amoebophrya*	
319	Chromista	Myzozoa	Dinophyceae	Syndiniales	Duboscquellidae	*Duboscquella*	
320	Chromista	Myzozoa	Dinophyceae	Syndiniales	Syndiniaceae	*Hematodinium*	*perezi*
321	Chromista	Myzozoa	Dinophyceae			*Stoeckeria*	
322	Chromista	Myzozoa	Gregarinasina	Eugregarinorida	Gregarinidae		
323	Chromista	Myzozoa	Gregarinasina	Eugregarinorida	Lecudinidae	*Lankesteria*	
324	Chromista	Myzozoa	Gregarinasina	Eugregarinorida	Lecudinidae	*Lecudina*	
325	Chromista	Myzozoa	Perkinsea	Perkinsida	Perkinsidae	*Perkinsus*	
326	Chromista	Ochrophyta	Bacillariophyceae	Achnanthales			
327	Chromista	Ochrophyta	Bacillariophyceae	Bacillariales	Bacillariaceae	*Cylindrotheca*	
328	Chromista	Ochrophyta	Bacillariophyceae	Bacillariales	Bacillariaceae	*Nitzschia*	
329	Chromista	Ochrophyta	Bacillariophyceae	Bacillariales	Bacillariaceae	*Pseudo-nitzschia*	
330	Chromista	Ochrophyta	Bacillariophyceae	Naviculales	Naviculaceae	*Meuniera*	
331	Chromista	Ochrophyta	Bacillariophyceae	Naviculales	Naviculaceae	*Navicula*	
332	Chromista	Ochrophyta	Bacillariophyceae	Naviculales	Pleurosigmataceae		
333	Chromista	Ochrophyta	Bacillariophyceae	Thalassiophysales	Catenulaceae	*Amphora*	
334	Chromista	Ochrophyta	Chrysophyceae	Chromulinales	Paraphysomonadaceae	*Paraphysomonas*	
335	Chromista	Ochrophyta	Coscinodiscophyceae	Chaetocerotales	Chaetocerotaceae	*Chaetoceros*	
336	Chromista	Ochrophyta	Coscinodiscophyceae	Corethrales	Corethraceae	*Corethron*	
337	Chromista	Ochrophyta	Coscinodiscophyceae	Coscinodiscales	Hemidiscaceae	*Actinocyclus*	
338	Chromista	Ochrophyta	Coscinodiscophyceae	Leptocylindrales	Leptocylindraceae		
339	Chromista	Ochrophyta	Coscinodiscophyceae	Rhizoleniales	Rhizosoleniaceae	*Guinardia*	
340	Chromista	Ochrophyta	Coscinodiscophyceae	Rhizoleniales	Rhizosoleniaceae	*Rhizosolenia*	
341	Chromista	Ochrophyta	Coscinodiscophyceae	Thalassiosirales	Skeletonemataceae	*Skeletonema*	*menzellii*
342	Chromista	Ochrophyta	Coscinodiscophyceae	Thalassiosirales	Thalassiosiraceae	*Minidiscus*	
343	Chromista	Ochrophyta	Coscinodiscophyceae	Thalassiosirales	Thalassiosiraceae	*Thalassiosira*	
344	Chromista	Ochrophyta	Dictyochophyceae	Florenciellales		*Florenciella*	
345	Chromista	Ochrophyta	Dictyochophyceae	Pedinellales		*Pseudopedinella*	
346	Chromista	Ochrophyta	Dictyochophyceae	Rhizochromulinales		*Rhizochromulina*	
347	Chromista	Ochrophyta	Fragilariophyceae	Fragilariales	Fragilariaceae	*Opephora*	
348	Chromista	Ochrophyta	Fragilariophyceae	Rhaponeidales	Rhaphoneidaceae		
349	Chromista	Ochrophyta	Fragilariophyceae	Striatellales		*Florella*	
350	Chromista	Ochrophyta	Fragilariophyceae	Thalssionematales	Thalssionemataceae	*Thalssionema*	
351	Chromista	Ochrophyta	Fragilariophyceae		Licmophoraceae	*Licmosphenia*	
352	Chromista	Ochrophyta	Mediophyceae	Cymatosirales	Cymatosiraceae	*Minutocellus*	
353	Chromista	Ochrophyta	Mediophyceae	Hemiaulales	Hemiaulaceae	*Cerataulina*	
354	Chromista	Ochrophyta	Mediophyceae	Thalassiosirales	Stephanodiscaceae	*Cyclotella*	
355	Chromista	Ochrophyta	Pelagophyceae	Pelagomonadales		*Pelagomonas*	
356	Chromista	Ochrophyta	Pelagophyceae	Sarcinochrysidales			
357	Chromista	Ochrophyta	Phaeophyceae	Dictyotales	Dictyotaceae	*Lobophora*	
358	Chromista	Ochrophyta	Phaeophyceae	Fucales	Sargassaceae	*Sargassum*	*ilicifolium*
359	Chromista	Ochrophyta	Raphidophyceae			*Psammamonas*	
360	Chromista	Ochrophyta		Dictyotales	Dictyotaceae	*Dictyota*	
361	Chromista	Ochrophyta		Dictyotales	Dictyotaceae	*Lobophora*	
362	Chromista	Ochrophyta		Ectocarpales	Chordariaceae		
363	Chromista	Ochrophyta		Ectocarpales	Scytosiphonaceae	*Hydroclathrus*	
364	Chromista	Ochrophyta		Fucales	Sargassaceae		
365	Chromista	Ochrophyta		Pinguiochrysidales	Pinguiochrysidaceae	*Pinguiochrysis*	
366	Chromista	Ochrophyta		Sphacelariales	Sphacelariaceae		
367	Chromista	Oomycota	Oomycetes	Lagenidiales	Haliphthoraceae		
368	Chromista	Oomycota	Oomycetes	Myzocytiopsidales	Eurychasmataceae		
369	Chromista	Oomycota	Oomycetes	Olpidiopsidales			
370	Chromista	Radiozoa	Acantharea	Arthracanthida			
371	Chromista	Radiozoa	Polycystinea	Spumellaria	Litheliidae	Larcopyle	
372	Chromista					*Chromerida*	
373	Fungi	Ascomycota	Dothideomycetes	Capnodiales	Cladosporiaceae	*Cladosporium*	
374	Fungi	Ascomycota	Dothideomycetes	Pleosporales	Pleosporaceae		
375	Fungi	Ascomycota	Eurotiomycetes	Eurotiales	Trichocomaceae	*Talaromyces*	
376	Fungi	Ascomycota	Saccharomycetes	Saccharomycetales			
377	Fungi	Basidiomycota	Agaricomycetes	Agaricales			
378	Fungi	Basidiomycota	Malasseziomycetes	Malasseziales	Malasseziaceae	*Malassezia*	
379	Fungi	Chytridiomycota	Chytridiomycetes	Chytridiales	Chytridiaceae	*Chytridium*	
380	Fungi	Entomophthoromycota	Basidiobolomycetes	Basidiobolales	Basidiobolaceae	*Basidiobolus*	

381	Plantae	Chlorophyta	Chlorodendrophyceae	Chlorodendrales	Chlorodendraceae	*Tetraselmis*
382	Plantae	Chlorophyta	Chlorophyceae	Chlamydomonadales		
383	Plantae	Chlorophyta	Mamiellophyceae	Dolichomastigaceae	Crustomastigaceae	
384	Plantae	Chlorophyta	Mamiellophyceae	Dolichomastigaceae	Dolichomastigaceae	
385	Plantae	Chlorophyta	Mamiellophyceae	Mamiellales	Bathycoccaceae	*Bathycoccus*
386	Plantae	Chlorophyta	Mamiellophyceae	Mamiellales	Bathycoccaceae	*Ostreococcus*
387	Plantae	Chlorophyta	Mamiellophyceae	Mamiellales	Mamiellaceae	*Mamiella*
388	Plantae	Chlorophyta	Mamiellophyceae	Mamiellales	Mamiellaceae	*Mantoniella*
389	Plantae	Chlorophyta	Mamiellophyceae	Mamiellales	Mamiellaceae	*Micromonas*
390	Plantae	Chlorophyta	Nephroselmidophyceae			*Nephroselmis*
391	Plantae	Chlorophyta	Prasinophyceae	Mamiellales	Mamiellaceae	*Mantoniella*
392	Plantae	Chlorophyta	Prasinophyceae	Pseudoscourfieldiales	Pycnococcaceae	*Prasinoderma*
393	Plantae	Chlorophyta	Prasinophyceae	Pseudoscourfieldiales	Pycnococcaceae	*Pseudoscourfieldia*
394	Plantae	Chlorophyta	Trebouxiophyceae			
395	Plantae	Chlorophyta	Ulvophyceae	Dasycladales	Polyphysaceae	*Acetabularia*
396	Plantae	Chlorophyta	Ulvophyceae	Oltmannsiellopsidales	Oltamannsiellopsidaceae	*Oltmannsiellopsis*
397	Plantae	Chlorophyta	Ulvophyceae	Ulvales	Ulvaceae	*Ulva*
398	Plantae	Chlorophyta	Ulvophyceae	Ulvales	Ulvellaceae	*Ochlochaete*
399	Plantae	Chlorophyta		Prasinococcales		*Prasinoderma*
400	Plantae	Chlorophyta		Pyramimonadales		*Pyramimonas*
401	Plantae	Chlorophyta			Prasinophyceae	
402	Plantae	Chlorophyta			Pycnococcaceae	*Pycnococcus*
403	Plantae	Glaucophyta	Glaucocystophyceae		Cyanophoraceae	*Cyanophora*
404	Plantae	Rhodophyta	Florideophyceae	Acrochaetiales	Acrochaetiaceae	*Acrochaetium*
405	Plantae	Rhodophyta	Florideophyceae	Ceramiales	Ceramiaceae	
406	Plantae	Rhodophyta	Florideophyceae	Ceramiales	Rhodomelaceae	
407	Plantae	Rhodophyta	Florideophyceae	Corallinales		
408	Plantae	Rhodophyta	Florideophyceae	Gigartinales	Peyssonneliaceae	*Sonderopelta*
409	Plantae	Rhodophyta	Florideophyceae	Gigartinales	Solieriaceae	
410	Plantae	Rhodophyta	Florideophyceae	Rhodymeniales	Lomentariaceae	
411	Plantae	Rhodophyta	Florideophyceae	Rhodymeniales	Rhodymeniaceae	*Coelothrix*
412	Plantae	Streptophyta	Equisetopsida	Apiales	Apiaceae	
413	Plantae	Streptophyta	Equisetopsida	Asterales	Asteraceae	
414	Plantae	Streptophyta	Equisetopsida	Brassicales	Caricaceae	
415	Plantae	Streptophyta	Equisetopsida	Caryophyllales	Amaranthaceae	
416	Plantae	Streptophyta	Equisetopsida	Cucurbitales	Cucurbitaceae	
417	Plantae	Streptophyta	Equisetopsida	Fabales	Fabaceae	
418	Plantae	Streptophyta	Equisetopsida	Poales	Poaceae	
419	Plantae	Streptophyta	Equisetopsida	Sapindales	Rutaceae	
420	Plantae	Streptophyta	Equisetopsida	Solanales	Solanaceae	*Solanum*
421	Plantae	Streptophyta		Lamiales	Acanthaceae	*Lankesteria*
422	Plantae	Streptophyta		Magnoliales	Magnoliaceae	
423	Protozoa	Amoebozoa		Dactylopodida	Vexilliferidae	*Neoparamoeba*
424	Protozoa	Apusozoa			Apusomonadidae	*Thecamonas*
425	Protozoa	Apusozoa			Planomonadidae	*Planomonas*
426	Protozoa	Apusozoa				*Ancyromonas*
427	Protozoa	Choanozoa	Choanoflagellatea	Choanoflagellida	Codonosigidae	*Monosiga*
428	Protozoa	Choanozoa	Choanoflagellatea	Choanoflagellida	Stephanoecidae	*Diaphanoeca*
429	Protozoa	Choanozoa	Choanoflagellatea	Choanoflagellida	Stephanoecidae	*Stephanoeca*
430	Protozoa	Choanozoa	Ichthyosporea			
431	Protozoa	Picozoa	Picomonadea	Picomonadida	Picomonadidae	
432	Protozoa	Picozoa				
433	Unranked					*Cantina*
434	Unranked					*Pirsonia*

Highlighted taxa were removed from the analysis

Supplementary Data 3 - 18SV4 taxa

Count	Kingdom	Phylum	Class	Order	Family	Genus	Species
1	Animalia	Annelida	Polychaeta	Capitellida	Capitellidae		
2	Animalia	Annelida	Polychaeta	Eunicida	Eunicidae	*Eunice*	
3	Animalia	Annelida	Polychaeta	Phyllodocida	Chrysopetalidae	*Chrysopetalum*	
4	Animalia	Annelida	Polychaeta	Phyllodocida	Hesionidae		
5	Animalia	Annelida	Polychaeta	Phyllodocida	Nereididae	*Ceratonereis*	
6	Animalia	Annelida	Polychaeta	Phyllodocida	Syllidae	*Branchiosyllis*	
7	Animalia	Annelida	Polychaeta	Phyllodocida	Syllidae	*Erinaceusyllis*	
8	Animalia	Annelida	Polychaeta	Phyllodocida	Syllidae	*Exogone*	
9	Animalia	Annelida	Polychaeta	Sabellida	Fabriciidae	*Fabricinuda*	
10	Animalia	Annelida	Polychaeta	Sabellida	Fabriciidae	*Pseudofabriciola*	
11	Animalia	Annelida	Polychaeta	Sabellida	Serpulidae		
12	Animalia	Annelida	Polychaeta	Spionida	Chaetopteridae		
13	Animalia	Annelida	Polychaeta	Spionida	Spionidae	*Aurospio*	
14	Animalia	Annelida	Polychaeta	Spionida	Spionidae	*Spio*	
15	Animalia	Annelida	Polychaeta	Terebellida	Terebellidae		
16	Animalia	Annelida	Polychaeta		Opheliidae		
17	Animalia	Annelida	Polychaeta		Orbiniidae		
18	Animalia	Annelida	Polychaeta		Protodrillidae	*Protodrilus*	
19	Animalia	Arthropoda	Maxillopoda	Cyclopoida			
20	Animalia	Arthropoda	Maxillopoda	Harpacticoida	Ameiridae		
21	Animalia	Arthropoda	Maxillopoda	Harpacticoida	Canuellidae		
22	Animalia	Arthropoda	Maxillopoda	Poecilostomatoida	Catiniidae		
23	Animalia	Arthropoda	Maxillopoda	Poecilostomatoida	Lichomolgidae	*Lichomolgus*	
24	Animalia	Arthropoda	Maxillopoda	Poecilostomatoida	Pseudanthessiidae	*Pseudanthessius*	
25	Animalia	Brachiopoda	Rhynchonellata				
26	Animalia	Chaetognatha		Biphragmophora	Spadellidae	*Spadella*	
27	Animalia	Chordata	Actinopterygii	Cluperiformes	Clupeidae		
28	Animalia	Chordata	Actinopterygii	Scorpaeniformes			
29	Animalia	Chordata	Actinopterygii	Siluriformes			
30	Animalia	Chordata	Actinopterygii	Tetraodontiformes	Tetraodontidae		
31	Animalia	Chordata	Ascidiacea				
32	Animalia	Chordata	Chondrichthyes	Myliobatiformes	Dasyatidae		
33	Animalia	Chordata	Ophiuroidea	Ophiurida			
34	Animalia	Cnidaria	Anthozoa	Actinaria			
35	Animalia	Cnidaria	Anthozoa	Scleractinia	Acroporidae	*Montipora*	
36	Animalia	Cnidaria	Anthozoa	Scleractinia	Pocilloporidae	*Pocillopora*	
37	Animalia	Cnidaria	Hydrozoa	Leptothecata	Campanulariidae		
38	Animalia	Cnidaria	Hydrozoa	Leptothecata	Mitrocomidae	*Mitrocomella*	
39	Animalia	Cnidaria	Hydrozoa	Leptothecata	Sertulariidae		
40	Animalia	Ctenophora					
41	Animalia	Echinodermata	Echinoidea				
42	Animalia	Echinodermata	Holothuroidea				
43	Animalia	Mollusca	Bivalvia	Limoida	Limidae	*Limaria*	
44	Animalia	Mollusca	Bivalvia	Lucinoida	Lucinidae		
45	Animalia	Mollusca	Bivalvia	Mytiloida	Mytilidae		
46	Animalia	Mollusca	Bivalvia	Ostreoida	Ostreidae	*Lopha*	
47	Animalia	Mollusca	Bivalvia	Pterioida	Isognomonidae	*Isognomon*	
48	Animalia	Mollusca	Bivalvia	Veneroida	Petricolidae		
49	Animalia	Mollusca	Gastropoda				
50	Animalia	Mollusca	Polyplacophora	Neoloricata	Mopaliidae		
51	Animalia	Nemertea	Enopla	Monostilifera	Tetrastemmatidae	*Tetrastemma*	
52	Animalia	Nemertea	Palaeonemertea		Cephalothricidae	*Cephalothrix*	
53	Animalia	Platyhelminthes	Rhabditophora	Polycladida			
54	Animalia	Platyhelminthes	Turbellaria	Macrostomida	Macrostomidae		
55	Animalia	Platyhelminthes	Turbellaria	Macrostomida	Microstomidae		
56	Animalia	Porifera	Calcarea				
57	Animalia	Porifera	Deomspongiae	Haplosclerida			
58	Animalia	Porifera	Homoscleromorpha	Homosclerophorida	Plakinidae		
59	Animalia	Xenacoelomorpha	Acoela		Isodiametridae		
60	Chromista	Bigyra	Labyrinthulomycetes		Thraustochytriaceae	*Aplanochytrium*	
61	Chromista	Bigyra		Bicosoecida	Bicosoecidae	*Bicosoeca*	
62	Chromista	Cercozoa	Sarcomonadea	Cercomonadida	Cercomonadidae	*Massisteria*	
63	Chromista	Cercozoa	Thecofilosea	Cryomonadida		*Cryothecomonas*	
64	Chromista	Ciliophora	Oligohymenophorea	Hymenostomatida			
65	Chromista	Ciliophora	Oligohymenophorea	Peniculida			
66	Chromista	Ciliophora	Oligohymenophorea	Philasterida	Cinetochilidae	*Cinetochilum*	
67	Chromista	Ciliophora	Oligohymenophorea	Philasterida	Cohnilembidae	*Cohnilembus*	
68	Chromista	Ciliophora	Oligohymenophorea	Philasterida	Cohnilembidae	*Porpostoma*	
69	Chromista	Ciliophora	Oligohymenophorea	Philasterida	Orchitophryidae	*Metanophrys*	

70	Chromista	Ciliophora	Oligohymenophorea	Philasterida	Orchitophryidae	*Paranophrys*	
71	Chromista	Ciliophora	Oligohymenophorea	Philasterida	Philasteridae		
72	Chromista	Ciliophora	Oligohymenophorea	Philasterida	Uronematidae	*Uronemella*	
73	Chromista	Ciliophora	Oligohymenophorea	Pleurondematida	Pleurodematidae	*Pleuronema*	*coronatum*
74	Chromista	Ciliophora	Phyllopharyngea	Dysteriida	Dysteriidae	*Dysteria*	
75	Chromista	Ciliophora	Prostomatea	Prorodontida	Colepidae	*Tiarina*	
76	Chromista	Ciliophora	Prostomatea	Prorodontida	Urotrichidae	*Urotricha*	
77	Chromista	Ciliophora	Spirotrichea	Choreotrichida	Strombidinopsidae	*Parastrombidinopsis*	
78	Chromista	Ciliophora	Spirotrichea	Euplotida	Uronychiidae	*Diophrys*	
79	Chromista	Ciliophora	Spirotrichea	Tintinnida			
80	Chromista	Ciliophora	Spirotrichea		Strombidiidae	*Strombidium*	
81	Chromista	Cryptophyta	Cryptophyceae	Cryptomonadales	Cryptomonadaceae		
82	Chromista	Cryptophyta	Cryptophyceae	Cryptomonadales	Goniomonadaceae	*Goniomonas*	
83	Chromista	Cryptophyta	Cryptophyceae	Cryptomonadales	Hemiselmidaceae	*Hemiselmis*	
84	Chromista	Cryptophyta	Cryptophyceae	Pyrenomonadales	Geminigeraceae	*Teleaulax*	
85	Chromista	Cryptophyta	Cryptophyceae	Pyrenomonadales	Pyrenomonadaceae	*Rhodomonas*	
86	Chromista	Cryptophyta	Katablepharidophyta		Katablepharidaceae		
87	Chromista	Cryptophyta	Telonemea	Telonemida		*Telonema*	
88	Chromista	Dinoflagellata	Dinophyceae	Gonyaulacales	Amphidomataceae	*Amphidoma*	
89	Chromista	Dinoflagellata	Dinophyceae	Gonyaulacales	Amphidomataceae	*Azadinium*	
90	Chromista	Dinoflagellata	Dinophyceae	Gonyaulacales	Ceratiaceae	*Neoceratium*	
91	Chromista	Dinoflagellata	Dinophyceae	Gonyaulacales	Gonyaulaceae	*Alexandrium*	
92	Chromista	Dinoflagellata	Dinophyceae	Gonyaulacales	Gonyaulaceae	*Gonyaulax*	
93	Chromista	Dinoflagellata	Dinophyceae	Gymnodiniales	Brachidiniaceae	*Brachidinium*	
94	Chromista	Dinoflagellata	Dinophyceae	Gymnodiniales	Gymnodiniaceae	*Amphidinium*	*klebsii*
95	Chromista	Dinoflagellata	Dinophyceae	Gymnodiniales	Gymnodiniaceae	*Cochlodinium*	
96	Chromista	Dinoflagellata	Dinophyceae	Gymnodiniales	Gymnodiniaceae	*Gymnodinium*	
97	Chromista	Dinoflagellata	Dinophyceae	Gymnodiniales	Gymnodiniaceae	*Gyrodinium*	
98	Chromista	Dinoflagellata	Dinophyceae	Gymnodiniales	Gymnodiniaceae	*Lepidodinium*	
99	Chromista	Dinoflagellata	Dinophyceae	Gymnodiniales	Gymnodiniaceae	*Paragymnodinium*	
100	Chromista	Dinoflagellata	Dinophyceae	Gymnodiniales	Kareniaceae	*Karenia*	
101	Chromista	Dinoflagellata	Dinophyceae	Gymnodiniales	Kareniaceae	*Karlodinium*	
102	Chromista	Dinoflagellata	Dinophyceae	Gymnodiniales	Warnowiaceae	*Warnowia*	
103	Chromista	Dinoflagellata	Dinophyceae	Peridiniales	Glenodiniaceae	*Glenodinium*	
104	Chromista	Dinoflagellata	Dinophyceae	Peridiniales	Heterocapsaceae	*Heterocapsa*	
105	Chromista	Dinoflagellata	Dinophyceae	Peridiniales	Peridiniaceae	*Pentapharsodinium*	
106	Chromista	Dinoflagellata	Dinophyceae	Peridiniales	Peridiniaceae	*Peridinium*	
107	Chromista	Dinoflagellata	Dinophyceae	Peridiniales	Podolampaceae	*Blepharocysta*	
108	Chromista	Dinoflagellata	Dinophyceae	Peridiniales	Podolampaceae	*Podolampas*	
109	Chromista	Dinoflagellata	Dinophyceae	Peridiniales	Protoperidiniaceae	*Protoperidinium*	
110	Chromista	Dinoflagellata	Dinophyceae	Peridiniales	Thoracosphaeraceae	*Scrippsiella*	
111	Chromista	Dinoflagellata	Dinophyceae	Peridiniales		*Galeidinium*	
112	Chromista	Dinoflagellata	Dinophyceae	Prorocentrales	Prorocentraceae	*Prorocentrum*	
113	Chromista	Dinoflagellata	Dinophyceae	Pyrocystales	Pyrocystaceae	*Dissodinium*	
114	Chromista	Dinoflagellata	Dinophyceae	Suessiales	Symbiodiniaceae	*Symbiodinium*	
115	Chromista	Dinoflagellata	Dinophyceae	Syndiniales	Amoebophryaceae	*Amoebophrya*	
116	Chromista	Dinoflagellata	Dinophyceae	Syndiniales	Syndiniaceae	*Hematodinium*	
117	Chromista	Dinoflagellata	Dinophyceae			*Stoeckeria*	
118	Chromista	Haptophyta	Chrysophyceae	Chromulinales	Chromulinaceae		
119	Chromista	Haptophyta	Chrysophyceae	Chromulinales	Paraphysomonadaceae	*Paraphysomonas*	
120	Chromista	Myzozoa	Perkinsea	Perkinsida	Perkinsidae		
121	Chromista	Ochrophyta	Bacillariophyceae	Achnanthales			
122	Chromista	Ochrophyta	Bacillariophyceae	Bacillariales	Bacillariaceae	*Cylindrotheca*	
123	Chromista	Ochrophyta	Bacillariophyceae	Bacillariales	Bacillariaceae	*Nitzschia*	
124	Chromista	Ochrophyta	Bacillariophyceae	Bacillariales	Bacillariaceae	*Pseudo-nitzschia*	
125	Chromista	Ochrophyta	Bacillariophyceae	Naviculales	Naviculaceae	*Meuniera*	
126	Chromista	Ochrophyta	Bacillariophyceae	Naviculales	Naviculaceae	*Navicula*	
127	Chromista	Ochrophyta	Bacillariophyceae	Naviculales	Pleurosigmataceae		
128	Chromista	Ochrophyta	Bacillariophyceae	Thalassiophysales	Catenulaceae	*Amphora*	
129	Chromista	Ochrophyta	Coscinodiscophyceae	Chaetocerotales	Chaetocerotaceae		
130	Chromista	Ochrophyta	Coscinodiscophyceae	Corethrales	Corethraceae	*Corethron*	
131	Chromista	Ochrophyta	Coscinodiscophyceae	Coscinodiscales	Hemidiscaceae	*Actinocyclus*	
132	Chromista	Ochrophyta	Coscinodiscophyceae	Leptocylindrales	Leptocylindraceae		
133	Chromista	Ochrophyta	Coscinodiscophyceae	Rhizoleniales	Rhizosoleniaceae	*Guinardia*	
134	Chromista	Ochrophyta	Coscinodiscophyceae	Rhizoleniales	Rhizosoleniaceae	*Rhizosolenia*	
135	Chromista	Ochrophyta	Coscinodiscophyceae	Thalassiosirales	Skeletonemataceae	*Skeletonema*	
136	Chromista	Ochrophyta	Coscinodiscophyceae	Thalassiosirales	Thalassiosiraceae	*Minidiscus*	
137	Chromista	Ochrophyta	Coscinodiscophyceae	Thalassiosirales	Thalassiosiraceae	*Thalassiosira*	
138	Chromista	Ochrophyta	Dictyochophyceae	Florenciellales		*Florenciella*	
139	Chromista	Ochrophyta	Dictyochophyceae	Pedinellales		*Pseudopedinella*	
140	Chromista	Ochrophyta	Fragilariophyceae	Fragilariales	Fragilariaceae	*Opephora*	

141	Chromista	Ochrophyta	Fragilariophyceae	Rhaponeidales	Rhaphoneidaceae	
142	Chromista	Ochrophyta	Fragilariophyceae	Striatellales		*Florella*
143	Chromista	Ochrophyta	Fragilariophyceae		Licmophoraceae	*Licmosphenia*
144	Chromista	Ochrophyta	Mediophyceae	Hemiaulales	Hemiaulaceae	
145	Chromista	Ochrophyta	Pelagophyceae	Pelagomonadales		*Pelagomonas*
146	Chromista	Ochrophyta	Pelagophyceae	Sarcinochrysidales		
147	Chromista	Ochrophyta	Raphidophyceae			*Psammamonas*
148	Chromista	Ochrophyta		Dictyotales	Dictyotaceae	*Lobophora*
149	Chromista	Ochrophyta		Fucales	Sargassaceae	
150	Chromista	Ochrophyta		Sphacelariales	Sphacelariaceae	
151	Chromista	Oomycota	Oomycetes	Lagenidiales	Haliphthoraceae	
152	Chromista	Oomycota	Oomycetes	Myzocytiopsidales	Eurychasmataceae	
153	Chromista	Oomycota	Oomycetes	Olpidiopsidales		
154	Chromista	Radiozoa	Acantharea	Arthracanthida		
155	Chromista	Radiozoa	Polycystinea	Spumellaria	Litheliidae	Larcopyle
156	Chromista					*Chromerida*
157	Fungi	Ascomycota	Dothideomycetes	Pleosporales	Pleosporaceae	
158	Fungi	Ascomycota	Eurotimycetes	Eurotiales		
159	Fungi	Ascomycota	Saccharomycetes	Saccharomycetales		
160	Fungi	Basidiomycota	Agaricomycetes	Agaricales		
161	Fungi	Basidiomycota	Malasseziomycetes	Malasseziales	Malasseziaceae	*Malassezia*
162	Fungi	Chytridiomycota	Chytridiomycetes	Chytridiales	Chytridiaceae	*Chytridium*
163	Fungi	Entomophthoromycota	Basidiobolomycetes	Basidiobales	Basidiobolaceae	*Basidiobolus*
164	Plantae	Chlorophyta	Chlorodendrophyceae	Chlorodendrales	Chlorodendraceae	*Tetraselmis*
165	Plantae	Chlorophyta	Chlorophyceae	Chlamydomonadales		
166	Plantae	Chlorophyta	Mamiellophyceae	Dolichomastigaceae	Crustomastigaceae	
167	Plantae	Chlorophyta	Mamiellophyceae	Dolichomastigaceae	Dolichomastigaceae	
168	Plantae	Chlorophyta	Mamiellophyceae	Mamiellales	Bathycoccaceae	*Bathycoccus*
169	Plantae	Chlorophyta	Mamiellophyceae	Mamiellales	Bathycoccaceae	*Ostreococcus*
170	Plantae	Chlorophyta	Mamiellophyceae	Mamiellales	Mamiellaceae	*Mamiella*
171	Plantae	Chlorophyta	Mamiellophyceae	Mamiellales	Mamiellaceae	*Mantoniella*
172	Plantae	Chlorophyta	Mamiellophyceae	Mamiellales	Mamiellaceae	*Micromonas*
173	Plantae	Chlorophyta	Nephroselmidophyceae			*Nephroselmis*
174	Plantae	Chlorophyta	Trebouxiophyceae			
175	Plantae	Chlorophyta	Ulvophyceae	Ulvales	Ulvaceae	*Ulva*
176	Plantae	Chlorophyta	Ulvophyceae	Ulvales	Ulvellaceae	*Ochlochaete*
177	Plantae	Chlorophyta		Prasinococcales		*Prasinoderma*
178	Plantae	Chlorophyta		Pyramimonadales		*Pyramimonas*
179	Plantae	Chlorophyta			Pycnococcaceae	*Pycnococcus*
180	Plantae	Glaucophyta	Glaucocystophyceae		Cyanophoraceae	*Cyanophora*
181	Plantae	Rhodophyta	Florideophyceae	Ceramiales	Ceramiaceae	
182	Plantae	Rhodophyta	Florideophyceae	Ceramiales	Rhodomelaceae	
183	Plantae	Rhodophyta	Florideophyceae	Rhodymeniales	Lomentariaceae	
184	Plantae	Streptophyta		Solonales	Solanaceae	*Solanum*
185	Protozoa	Amoebozoa		Dactylopodida	Vexilliferidae	*Neoparamoeba*
186	Protozoa	Apusozoa				*Ancyromonas*
187	Protozoa	Choanozoa	Choanoflagellatea	Choanoflagellida	Stephanoecidae	*Stephanoeca*
188	Protozoa	Choanozoa	Ichthyosporea			
189	Protozoa	Picozoa	Picomonadea	Picomonadida	Picomonadidae	
190						*Cantina*
191						*Pirsonia*

Supplementary Data 3 - 18S V1-V3 taxa

Count	Kingdom	Phylum	Class	Order	Family	Genus	Species
1	Animalia	Annelida	Phascolosomatidea	Phascolosomatiformes	Phascolosomatidae	*Phascolosoma*	
2	Animalia	Annelida	Polychaeta	Capitellida	Capitellidae	*Dasybranchus*	
3	Animalia	Annelida	Polychaeta	Eunicida	Eunicidae	*Eunice*	
4	Animalia	Annelida	Polychaeta	Phyllodocida	Syllidae	*Salvatoria*	
5	Animalia	Annelida	Polychaeta	Sabellida	Fabriciidae	*Fabricinuda*	
6	Animalia	Annelida	Polychaeta	Sabellida	Sabellidae		
7	Animalia	Annelida	Polychaeta		Opheliidae		
8	Animalia	Annelida	Polychaeta		Spionidae	*Spio*	
9	Animalia	Annelida	Polychaeta		Terebellidae		
10	Animalia	Arthropoda	Maxillopoda	Calanoida	Acartiidae		
11	Animalia	Arthropoda	Maxillopoda	Cyclopoida			
12	Animalia	Arthropoda	Maxillopoda	Poecilostomatoida	Catiniidae		
13	Animalia	Arthropoda	Maxillopoda	Poecilostomatoida	Lichomolgidae	*Lichomolgus*	
14	Animalia	Chordata	Actinopterygii	Perciformes			
15	Animalia	Chordata	Ascidiaceae	Pleurogona	Stylelidae	*Botryllus*	
16	Animalia	Chordata	Ascidiaceae	Stolidobranchia	Pyuridae	*Pyura*	
17	Animalia	Cnidaria	Anthozoa	Actiniaria			
18	Animalia	Cnidaria	Hydrozoa	Leptothecata			
19	Animalia	Cnidaria		Bivalvulida	Myxidiiadae		
20	Animalia	Ctenophora	Tentaculata	Platyctenida	Coeloplanidae	*Vallicula*	
21	Animalia	Echinodermata	Echinoidea				
22	Animalia	Gastrotricha		Chaetonotida	Chaetonotidae		
23	Animalia	Mollusca	Bivalvia	Limoida	Limidae	*Limaria*	
24	Animalia	Mollusca	Bivalvia	Mytiloida	Mytilidae		
25	Animalia	Mollusca	Bivalvia	Ostreoida	Ostreidae		
26	Animalia	Mollusca	Bivalvia	Solemyoida	Solemyidae	*Solemya*	
27	Animalia	Mollusca	Bivalvia	Veneroida	Cardiidae	*Fragum*	
28	Animalia	Mollusca	Bivalvia	Veneroida	Lucinidae		
29	Animalia	Mollusca	Gastropoda		Strombidae	*Lambis*	
30	Animalia	Nematoda	Chromadorea	Chromadorida	Chromadoridae		
31	Animalia	Nemertea	Enopla				
32	Animalia	Platyhelminthes	Monogenea				
33	Animalia	Platyhelminthes	Trematoda				
34	Animalia	Platyhelminthes	Turbellaria	Macrostomida	Macrostomidae		
35	Animalia	Platyhelminthes	Turbellaria	Rhabdocoela			
36	Animalia	Porifera	Calcarea				
37	Animalia	Porifera	Demospongiae	Dictyoceratida			
38	Animalia	Porifera	Demospongiae	Haplosclerida			
39	Animalia	Xenacoelomorpha	Acoela		Isodiametridae		
40	Chromista	Bigyra	Labyrinthulomycetes	Labyrinthulomycetes	Thraustochytriaceae	*Aplanochytrium*	
41	Chromista	Bigyra		Bicosoecida		*Ceacitellus*	
42	Chromista	Cercozoa	Imbricatea	Thaumatomanadida	Thaumatomastigidae		
43	Chromista	Ciliophora	Heterotrichea	Heterotrichida	Condylostomatidae	*Condylostoma*	
44	Chromista	Ciliophora	Litostomatea	Haptorida	Lacrymariidae	*Lacrymaria*	
45	Chromista	Ciliophora	Litostomatea	Pleurostomatida	Litonotidae	*Litonotus*	
46	Chromista	Ciliophora	Nassophorea	Synhymeniida	Orthodonellidae	*Zosterodasys*	
47	Chromista	Ciliophora	Oligohymenophorea	Philasterida	Loxocephalidae	*Cardiostomatella*	
48	Chromista	Ciliophora	Oligohymenophorea	Philasterida	Uronematidae	*Uronema*	*heteromarinum*
49	Chromista	Ciliophora	Oligohymenophorea	Pleuronematida	Cyclidiidae	*Cyclidium*	
50	Chromista	Ciliophora	Spirotrichea	Choreotrichida	Strobilidiidae	*Pelagostrobilidium*	
51	Chromista	Ciliophora	Spirotrichea	Choreotrichida	Strobilidiidae	*Rimostrombidium*	
52	Chromista	Ciliophora	Spirotrichea	Choreotrichida	Strombidinopsidae	*Strombidinopsis*	
53	Chromista	Ciliophora	Spirotrichea	Euplotida	Uronychiidae	*Diophrys*	
54	Chromista	Ciliophora	Spirotrichea	Sporadotrichida	Oxytrichidae	*Hemigastrostyla*	
55	Chromista	Ciliophora	Spirotrichea	Tintinnida	Tintinnidae		
56	Chromista	Ciliophora	Spirotrichea	Urostylida	Holostichidae	*Holosticha*	*diademata*
57	Chromista	Ciliophora	Spirotrichea		Cyrtostrombidiidae	*Cyrtostrombidium*	
58	Chromista	Ciliophora	Spirotrichea		Strombidiidae	*Strombidium*	
59	Chromista	Ciliophora	Spirotrichea		Totoniidae	*Pseudotontonia*	
60	Chromista	Ciliophora	Spirotrichea			*Protocruzia*	
61	Chromista	Cryptophyta	Cryptophyceae	Cryptomonadales	Goniomonadaceae	*Goniomonas*	
62	Chromista	Cryptophyta	Cryptophyceae	Pyrenomonadales	Geminigeraceae	*Proteomonas*	
63	Chromista	Cryptophyta	Cryptophyceae	Pyrenomonadales	Geminigeraceae	*Teleaulax*	
64	Chromista	Cryptophyta	Telonemea	Telonemida		*Telonema*	
65	Chromista	Haptophyta	Coccolithophyceae	Phaeocystales	Phaeocystaceae	*Phaeocystis*	
66	Chromista	Haptophyta	Coccolithophyceae	Prymnesiales	Chrysochromulinaceae	*Chrysochromulina*	
67	Chromista	Haptophyta	Pavlovophycidae	Pavlovales	Pavlovaceae	*Pavlova*	*pinguis*
68	Chromista	Heliozoa	Centrohelea	Centrohelida	Acanthocystidae	*Raineriophrys*	
69	Chromista	Myzozoa	Dinophyceae	Gonyaulacales	Amphidomataceae	*Amphidoma*	
70	Chromista	Myzozoa	Dinophyceae	Gonyaulacales	Amphidomataceae	*Azadinium*	
71	Chromista	Myzozoa	Dinophyceae	Gonyaulacales	Gonyaulacaceae	*Alexandrium*	

72	Chromista	Myzozoa	Dinophyceae	Gonyaulacales	Gonyaulacaceae	*Gonyaulax*	
73	Chromista	Myzozoa	Dinophyceae	Gymnodiniales	Gymnodiniaceae	*Amphidinium*	
74	Chromista	Myzozoa	Dinophyceae	Gymnodiniales	Gymnodiniaceae	*Gymnodinium*	
75	Chromista	Myzozoa	Dinophyceae	Gymnodiniales	Gymnodiniaceae	*Gyrodinium*	
76	Chromista	Myzozoa	Dinophyceae	Gymnodiniales	Gymnodiniaceae	*Paragymnodinium*	
77	Chromista	Myzozoa	Dinophyceae	Lophodiniales	Lophodiniaceae	*Woloszynskia*	
78	Chromista	Myzozoa	Dinophyceae	Peridiniales	Heterocapsaceae	*Heterocapsa*	
79	Chromista	Myzozoa	Dinophyceae	Peridiniales	Peridiniaceae	*Pentapharsodinium*	
80	Chromista	Myzozoa	Dinophyceae	Peridiniales	Peridiniaceae	*Peridinium*	
81	Chromista	Myzozoa	Dinophyceae	Peridiniales	Pfiesteriaceae		
82	Chromista	Myzozoa	Dinophyceae	Peridiniales	Protoperidiniaceae	*Protoperidinium*	
83	Chromista	Myzozoa	Dinophyceae	Peridiniales		*Galeidinium*	
84	Chromista	Myzozoa	Dinophyceae	Prorocentrales	Prorocentraceae	*Exuviaella*	
85	Chromista	Myzozoa	Dinophyceae	Prorocentrales	Prorocentraceae	*Prorocentrum*	
86	Chromista	Myzozoa	Dinophyceae	Syndiniales	Amoebophryaceae	*Amoebophrya*	
87	Chromista	Myzozoa	Dinophyceae	Syndiniales	Duboscquellidae	*Duboscquella*	
88	Chromista	Myzozoa	Dinophyceae	Syndiniales	Syndiniaceae	*Hematodinium*	*perezi*
89	Chromista	Myzozoa	Gregarinasina	Eugregarinorida	Gregarinidae		
90	Chromista	Myzozoa	Gregarinasina	Eugregarinorida	Lecudinidae	*Lankesteria*	
91	Chromista	Myzozoa	Gregarinasina	Eugregarinorida	Lecudinidae	*Lecudina*	
92	Chromista	Myzozoa	Perkinsea	Perkinsida	Perkinsidae	*Perkinsus*	
93	Chromista	Ochrophyta	Bacilliariophyceae	Bacilliariales	Bacillariaceae	*Pseudo-nitzschia*	
94	Chromista	Ochrophyta	Coscinodiscophyceae	Chaetocerotales	Chaetocerotaceae	*Chaetoceros*	
95	Chromista	Ochrophyta	Coscinodiscophyceae	Coscinodiscales	Hemidiscaceae	*Actinocyclus*	
96	Chromista	Ochrophyta	Coscinodiscophyceae	Thalassiosirales	Skeletonemataceae	*Skeletonema*	
97	Chromista	Ochrophyta	Coscinodiscophyceae	Thalassiosirales	Thalassiosiraceae	*Thalassiosira*	
98	Chromista	Ochrophyta	Dictyochophyceae	Florenciellales		*Florenciella*	
99	Chromista	Ochrophyta	Dictyochophyceae	Rhizochromulinales		*Rhizochromulina*	
100	Chromista	Ochrophyta	Fragilariophyceae	Thalssionematales	Thalssionemataceae	*Thalssionema*	
101	Chromista	Ochrophyta	Mediophyceae	Cymatosirales	Cymatosiraceae		
102	Chromista	Ochrophyta	Mediophyceae	Hemiaulales	Hemiaulaceae	*Cerataulina*	
103	Chromista	Ochrophyta	Phaeophyceae	Dictyotales	Dictyotaceae	*Lobophora*	
104	Chromista	Ochrophyta		Pinguiochrysidales	Pinguiochrysidaceae	*Pinguiochrysis*	
105	Fungi	Ascomycota	Dothideomycetes	Capnodiales	Cladosporiaceae	*Cladosporium*	
106	Fungi	Chytridiomycota					
107	Plantae	Chlorophyta	Chlorodendrophyeae	Chlorodendrales	Chlorodendraceae	*Tetraselmis*	
108	Plantae	Chlorophyta	Chlorophyceae	Chlamydomonadales			
109	Plantae	Chlorophyta	Mamiellophyceae	Mamiellales	Bathycoccaceae	*Bathycoccus*	
110	Plantae	Chlorophyta	Mamiellophyceae	Mamiellales	Bathycoccaceae	*Ostreococcus*	
111	Plantae	Chlorophyta	Mamiellophyceae	Mamiellales	Mamiellaceae	*Mamiella*	
112	Plantae	Chlorophyta	Mamiellophyceae	Mamiellales	Mamiellaceae	*Micromonas*	
113	Plantae	Chlorophyta	Ulvophyceae	Ulvales			
114	Plantae	Chlorophyta		Prasinococcales		*Prasinoderma*	
115	Plantae	Chlorophyta		Pyramimonadales		*Pyramimonas*	
116	Plantae	Chlorophyta			Prasinophyceae		
117	Plantae	Chlorophyta				*Oltmannsiellopsis*	
118	Plantae	Glaucophyta	Glaucocystophyceae		Cyanophoraceae	*Cyanophora*	
119	Plantae	Streptophyta		Lamiales	Acanthaceae	*Lankesteria*	
120	Protozoa	Amoebozoa		Dactylopodida	Vexilliferidae	*Neoparamoeba*	
121	Protozoa	Apusozoa			Planomonadidae	*Planomonas*	
122	Protozoa	Choanozoa	Choanoflagellatea	Choanoflagellida	Codonosigidae	*Monosiga*	
123	Protozoa	Choanozoa	Choanoflagellatea	Choanoflagellida	Stephanoecidae	*Diaphanoeca*	

Supplementary Data 3 - COI (metazoans) taxa

Count	Kingdom	Phylum	Class	Order	Family	Genus	Species
1	Animalia	Annelida	Polychaeta		Chaetopteridae	*Phyllochaetopterus*	
2	Animalia	Annelida	Polychaeta		Dorvilleidae	*Dorvillea*	
3	Animalia	Annelida	Polychaeta		Eunicidae		
4	Animalia	Arthropoda	Malacostraca	Decapoda	Xanthidae	*Etisus*	
5	Animalia	Arthropoda	Maxillopoda	Cyclopoida			
6	Animalia	Chordata	Actinopterygii	Atheriniformes	Atherinidae	*Atherinomorus*	
7	Animalia	Chordata	Actinopterygii	Atheriniformes	Atherinidae	*Hypoatherina*	
8	Animalia	Chordata	Actinopterygii	Beloniformes	Belonidae	*Tylosurus*	*gavialoides*
9	Animalia	Chordata	Actinopterygii	Cluperiformes	Clupeidae	*Spratelloides*	
10	Animalia	Chordata	Actinopterygii	Mugiliformes	Mugilidae	*Mugil*	*cephalus*
11	Animalia	Chordata	Actinopterygii	Perciformes	Carangidae	*Scomberoides*	*commersonnianus*
12	Animalia	Chordata	Actinopterygii	Perciformes	Gerreidae	*Gerres*	
13	Animalia	Chordata	Actinopterygii	Perciformes	Labridae	*Chlorurus*	*sordidus*
14	Animalia	Chordata	Actinopterygii	Perciformes	Labridae	*Coris*	*aygula*
15	Animalia	Chordata	Actinopterygii	Perciformes	Lethrinidae	*Lethrinus*	*nebulosus*
16	Animalia	Chordata	Actinopterygii	Perciformes	Pomacentridae	*Dascyllus*	*aruanus*
17	Animalia	Chordata	Actinopterygii	Perciformes	Pomacentridae	*Pomacentrus*	*coelestis*
18	Animalia	Chordata	Actinopterygii	Perciformes	Priacanthidae	*Heteropriacanthus*	
19	Animalia	Chordata	Actinopterygii	Perciformes	Scatophagidae	*Selenotoca*	
20	Animalia	Chordata	Actinopterygii	Tetraodontiformes	Tetraodontidae	*Arothron*	*hispidus*
21	Animalia	Chordata	Ascidiacea	Enterogona	Ascidiidae	*Ascidia*	
22	Animalia	Chordata	Chondrichthyes	Myliobatiformes	Dasyatidae	*Himantura*	*leoparda*
23	Animalia	Chordata	Chondrichthyes	Myliobatiformes	Dasyatidae	*Neotrygon*	*ningalooensis*
24	Animalia	Chordata	Chondrichthyes	Myliobatiformes	Dasyatidae	*Pastinachus*	*atrus*
25	Animalia	Chordata	Chondrichthyes	Myliobatiformes	Dasyatidae	*Taeniura*	
26	Animalia	Cnidaria	Anthozoa	Alcyonacea	Alcyoniidae		
27	Animalia	Cnidaria	Anthozoa	Scleractinia	Pocilloporidae	*Pocillopora*	
28	Animalia	Cnidaria	Hydrozoa	Leptothecata	Campanulariidae	*Campanularia*	
29	Animalia	Cnidaria	Hydrozoa	Leptothecata	Campanulariidae	*Clytia*	
30	Animalia	Cnidaria	Hydrozoa	Leptothecata	Haleciidae	*Halecium*	
31	Animalia	Echinodermata	Holothuroidea	Aspidochirotida	Holothuridae	*Holothuria*	*atra*
32	Animalia	Mollusca	Bivalvia	Solemyida	Solemyidae	*Solemya*	
33	Animalia	Mollusca	Cephalopoda	Octopoda	Octopodidae	*Octopus*	*cyanea*
34	Animalia	Mollusca	Gastropoda	Cerithimorpha	Cerithidae	*Cerithium*	
35	Animalia	Mollusca	Gastropoda	Hypsogastropoda	Strombidae	*Strombus*	
36	Animalia	Mollusca	Gastropoda		Littorinidae	*Echinolittorina*	
37	Animalia	Porifera	Demospongiae	Hadromerida	Clionaidae	*Pione*	
38	Animalia	Porifera	Demospongiae	Halichondrida	Halichondriidae	*Halichondria*	
39	Animalia	Porifera	Demospongiae	Halichondrida	Halichondriidae	*Hymeniacidon*	
40	Animalia	Porifera	Demospongiae	Haplosclerida			
41	Animalia	Porifera	Demospongiae	Poecilosclerida	Desmacellidae	*Biemna*	
42	Animalia	Porifera	Demospongiae	Poecilosclerida	Microcionidae	*Clathria*	
43	Animalia	Porifera		Homosclerophorida	Oscarellidae	*Oscarella*	
44	Chromista	Cercozoa	Chlorarachnea	Chlorachniida	Chlorarachniaceae	*Bigelowiella*	
45	Chromista	Haptophyta	Coccolithophyceae	Phaeocystales	Phaeocystaceae	*Phaeocystis*	
46	Chromista	Haptophyta	Coccolithophyceae	Prymnesiales	Chrysochromulinaceae	*Chrysochromulina*	
47	Chromista	Haptophyta	Prymnesiophyceae	Isochrysidales	Noelaerhabdaceae	*Emiliania*	
48	Chromista	Myzozoa	Dinophyceae	Gymnodiniales	Gymnodiniaceae	*Gymnodinium*	
49	Chromista	Myzozoa	Dinophyceae	Syndiniales	Syndiniaceae	*Hematodinium*	
50	Chromista	Ochrophyta	Bacillariophyceae	Bacillariales	Bacillariaceae	*Cylindrotheca*	
51	Chromista	Ochrophyta	Bacillariophyceae	Bacillariales	Bacillariaceae	*Nitzschia*	
52	Chromista	Ochrophyta	Bacillariophyceae	Bacillariales	Bacillariaceae	*Pseudo-nitzschia*	
53	Chromista	Ochrophyta	Coscinodiscophyceae	Chaetocerotales	Chaetocerotaceae	*Chaetoceros*	
54	Chromista	Ochrophyta	Coscinodiscophyceae	Thalassiosirales	Skeletonemataceae	*Skeletonema*	*menzellii*
55	Chromista	Ochrophyta	Coscinodiscophyceae	Thalassiosirales	Thalassiosiraceae	*Thalassiosira*	
56	Chromista	Ochrophyta	Mediophyceae	Cymatosirales	Cymatosiraceae	*Minutocellus*	
57	Chromista	Ochrophyta	Mediophyceae	Thalassiosirales	Stephanodiscaceae	*Cyclotella*	
58	Chromista	Ochrophyta	Phaeophyceae	Dictyotales	Dictyotaceae	*Dictyota*	
59	Chromista	Ochrophyta	Phaeophyceae	Fucales	Sargassaceae	*Sargassum*	*ilicifolium*
60	Chromista	Ochrophyta		Ectocarpales	Chordariaceae		
61	Chromista	Ochrophyta		Ectocarpales	Scytosiphonaceae	*Hydroclathrus*	
62	Fungi	Ascomycota	Eurotiomycetes	Eurotiales	Trichocomaceae	*Talaromyces*	
63	Plantae	Chlorophyta	Prasinophyceae	Mamiellales	Mamiellaceae	*Mantoniella*	
64	Plantae	Chlorophyta	Prasinophyceae	Mamiellales	Mamiellaceae	*Micromonas*	
65	Plantae	Chlorophyta	Prasinophyceae	Pseudoscourfieldiales	Pycnococcaceae	*Prasinoderma*	
66	Plantae	Chlorophyta	Prasinophyceae	Pseudoscourfieldiales	Pycnococcaceae	*Pseudoscourfieldia*	
67	Plantae	Chlorophyta	Ulvophyceae	Dasycladales	Polyphysaceae	*Acetabularia*	
68	Plantae	Chlorophyta	Ulvophyceae	Oltmannsiellopsidales	Oltamannsiellopsidaceae	*Oltmannsiellopsis*	
69	Plantae	Rhodophyta	Florideophyceae	Acrochaetiales	Acrochaetiaceae	*Acrochaetium*	
70	Plantae	Rhodophyta	Florideophyceae	Ceramiales	Rhodomelaceae		

71	Plantae	Rhodophyta	Florideophyceae	Corallinales		
72	Plantae	Rhodophyta	Florideophyceae	Gigartinales	Peyssonneliaceae	*Sonderopelta*
73	Plantae	Rhodophyta	Florideophyceae	Gigartinales	Solieriaceae	
74	Plantae	Rhodophyta	Florideophyceae	Rhodymeniales	Rhodymeniaceae	*Coelothrix*
75	Protozoa	Apusozoa			Apusomonadidae	*Thecamonas*

Supplementary Data 3 - Fish 16S taxa

**Count**	**Kingdom**	**Phylum**	**Class**	**Order**	**Family**	**Genus**	**Species**
1	Animalia	Chordata	Actinopterygii	Anguiliformes	Muraenidae	*Gymnothorax*	*pseudothyrsoideus*
2	Animalia	Chordata	Actinopterygii	Atheriniformes	Atherinidae	*Atherinomorus*	
3	Animalia	Chordata	Actinopterygii	Atheriniformes	Atherinidae	*Hypoatherina*	
4	Animalia	Chordata	Actinopterygii	Beloniformes	Belonidae	*Strongylura*	
5	Animalia	Chordata	Actinopterygii	Beloniformes	Hemiramphidae	*Hyporhamphus*	
6	Animalia	Chordata	Actinopterygii	Beryciformes	Holocentridae	*Sargocentron*	*rubrum*
7	Animalia	Chordata	Actinopterygii	Cluperiformes	Clupeidae	*Sardinella*	
8	Animalia	Chordata	Actinopterygii	Cluperiformes	Clupeidae	*Spratelloides*	
9	Animalia	Chordata	Actinopterygii	Cluperiformes	Engrulidae	*Engraulis*	
10	Animalia	Chordata	Actinopterygii	Mugiliformes	Mugilidae	*Mugil*	*cepahalus*
11	Animalia	Chordata	Actinopterygii	Perciformes	Acanthuridae	*Acanthurus*	*triostegus*
12	Animalia	Chordata	Actinopterygii	Perciformes	Apogonidae	*Apogon*	
13	Animalia	Chordata	Actinopterygii	Perciformes	Blenniidae	*Salarias*	
14	Animalia	Chordata	Actinopterygii	Perciformes	Carangidae	*Alectis*	
15	Animalia	Chordata	Actinopterygii	Perciformes	Carangidae	*Caranx*	
16	Animalia	Chordata	Actinopterygii	Perciformes	Carangidae	*Gnathanodon*	*speciosus*
17	Animalia	Chordata	Actinopterygii	Perciformes	Carangidae	*Scomberoides*	
18	Animalia	Chordata	Actinopterygii	Perciformes	Carangidae	*Trachinotus*	*blochii*
19	Animalia	Chordata	Actinopterygii	Perciformes	Chaetodontidae	*Chaetodon*	
20	Animalia	Chordata	Actinopterygii	Perciformes	Gerreidae	*Gerres*	
21	Animalia	Chordata	Actinopterygii	Perciformes	Gobiidae	*Asterropteryx*	
22	Animalia	Chordata	Actinopterygii	Perciformes	Gobiidae	*Bathygobius*	
23	Animalia	Chordata	Actinopterygii	Perciformes	Gobiidae	*Gnatholepis*	
24	Animalia	Chordata	Actinopterygii	Perciformes	Gobiidae	*Gobiodon*	*axillaris*
25	Animalia	Chordata	Actinopterygii	Perciformes	Haemulidae	*Plectorhinchus*	
26	Animalia	Chordata	Actinopterygii	Perciformes	Kyphosidae	*Kyphosus*	*bigibbus*
27	Animalia	Chordata	Actinopterygii	Perciformes	Labridae	*Anampses*	
28	Animalia	Chordata	Actinopterygii	Perciformes	Labridae	*Cheilinus*	*trilobatus*
29	Animalia	Chordata	Actinopterygii	Perciformes	Labridae	*Cheilio*	*inermis*
30	Animalia	Chordata	Actinopterygii	Perciformes	Labridae	*Chlorurus*	
31	Animalia	Chordata	Actinopterygii	Perciformes	Labridae	*Coris*	*aygula*
32	Animalia	Chordata	Actinopterygii	Perciformes	Labridae	*Cymolutes*	
33	Animalia	Chordata	Actinopterygii	Perciformes	Labridae	*Halichoeres*	*nebulosus*
34	Animalia	Chordata	Actinopterygii	Perciformes	Labridae	*Hemigymnus*	*melapterus*
35	Animalia	Chordata	Actinopterygii	Perciformes	Labridae	*Labrichthys*	
36	Animalia	Chordata	Actinopterygii	Perciformes	Labridae	*Labroides*	*dimidiatus*
37	Animalia	Chordata	Actinopterygii	Perciformes	Labridae	*Stethojulis*	
38	Animalia	Chordata	Actinopterygii	Perciformes	Labridae	*Thalassoma*	*lunare*
39	Animalia	Chordata	Actinopterygii	Perciformes	Lethrinidae	*Lethrinus*	*nebulosus*
40	Animalia	Chordata	Actinopterygii	Perciformes	Lutjanidae	*Lutjanus*	*fulviflamma*
41	Animalia	Chordata	Actinopterygii	Perciformes	Microdesmidae	*Gunnellichthys*	
42	Animalia	Chordata	Actinopterygii	Perciformes	Mullidae	*Parupeneus*	
43	Animalia	Chordata	Actinopterygii	Perciformes	Pinguipedidae		
44	Animalia	Chordata	Actinopterygii	Perciformes	Pomacentridae	*Abudefduf*	*vaigiensis*
45	Animalia	Chordata	Actinopterygii	Perciformes	Pomacentridae	*Cheiloprion*	*labiatus*
46	Animalia	Chordata	Actinopterygii	Perciformes	Pomacentridae	*Chromis*	*viridis*
47	Animalia	Chordata	Actinopterygii	Perciformes	Pomacentridae	*Dascyllus*	*aruanus*
48	Animalia	Chordata	Actinopterygii	Perciformes	Pomacentridae	*Pomacentrus*	*moluccensis*
49	Animalia	Chordata	Actinopterygii	Perciformes	Pomacentridae	*Stegastes*	*nigricans*
50	Animalia	Chordata	Actinopterygii	Perciformes	Pseudochromidae	*Psedodochromis*	
51	Animalia	Chordata	Actinopterygii	Perciformes	Labridae	*Hipposcarus*	
52	Animalia	Chordata	Actinopterygii	Perciformes	Labridae	*Scarus*	*schlegeli*
53	Animalia	Chordata	Actinopterygii	Perciformes	Scatophagidae		
54	Animalia	Chordata	Actinopterygii	Perciformes	Serranidae	*Epinephelus*	*quoyanus*
55	Animalia	Chordata	Actinopterygii	Perciformes	Siganidae	*Siganus*	
56	Animalia	Chordata	Actinopterygii	Perciformes	Sillaginidae	*Sillago*	
57	Animalia	Chordata	Actinopterygii	Perciformes	Sphyraenidae	*Sphyraena*	
58	Animalia	Chordata	Actinopterygii	Scorpaeniformes	Sebastidae		
59	Animalia	Chordata	Actinopterygii	Syngnathiformes	Fistularidae	*Fistularia*	

60	Animalia	Chordata	Actinopterygii	Tetraodontiformes	Balistidae	*Rhinecanthus*	*aculeatus*
61	Animalia	Chordata	Actinopterygii	Tetraodontiformes	Diodontidae	*Diodon*	*hystrix*
62	Animalia	Chordata	Actinopterygii	Tetraodontiformes	Monacanthidae	*Pervagor*	
63	Animalia	Chordata	Actinopterygii	Tetraodontiformes	Ostraciidae	*Ostracion*	*cubicus*
64	Animalia	Chordata	Actinopterygii	Tetraodontiformes	Tetraodontidae	*Arothron*	*hispidis*
65	Animalia	Chordata	Chondrichthyes	Myliobatiformes	Myliobatidae	*Aetobatus*	

Supplementary Data 3 - 16S mammals

Count	Kingdom	Phylum	Class	Order	Family	Genus	Species
1	Animalia	Chordata	Actinopterygii	Perciformes	Acanthuridae	*Acanthurus*	*triostegus*
2	Animalia	Chordata	Actinopterygii	Perciformes	Pomacentridae	*Stegastes*	*nigricans*
	Animalia	Chordata	Mammalia	Primates	Hominidae	*Homo*	*sapiens*
3	Animalia	Chordata	Mammalia	Cetacea	Delphinidae		
	Animalia	Chordata	Mammalia	Artiodactyla	Bovidae	*Bos*	
	Animalia	Chordata	Mammalia	Artiodactyla	Suidae	*Sus*	*scrofa*

Supplementary Data 3 - 16S crust

Count	Kingdom	Phylum	Class	Order	Family	Genus	Species
1	Animalia	Annelida	Polychaeta		Spionidae		
2	Animalia	Arthropoda	Insecta	Blattodea			
3	Animalia	Arthropoda	Insecta	Coleoptera	Endomychidae		
4	Animalia	Arthropoda	Insecta	Hemiptera	Cicadellidae	*Opsius*	
5	Animalia	Arthropoda	Insecta	Hymenoptera	Formicidae	*Pheidole*	
6	Animalia	Arthropoda	Malacostraca	Decapoda	Alpheidae	*Alpheus*	
7	Animalia	Arthropoda	Malacostraca	Decapoda	Axiidae	*Axiopsis*	
8	Animalia	Arthropoda	Malacostraca	Decapoda	Callianassidae		
9	Animalia	Arthropoda	Malacostraca	Decapoda	Diogenidae	*Calcinus*	*latens*
10	Animalia	Arthropoda	Malacostraca	Decapoda	Diogenidae	*Clibanarius*	
11	Animalia	Arthropoda	Malacostraca	Decapoda	Domeciidae		
12	Animalia	Arthropoda	Malacostraca	Decapoda	Dromiidae		
13	Animalia	Arthropoda	Malacostraca	Decapoda	Epialtinae		
14	Animalia	Arthropoda	Malacostraca	Decapoda	Galatheidae		
15	Animalia	Arthropoda	Malacostraca	Decapoda	Grapsidae	*Grapsus*	*albolineatus*
16	Animalia	Arthropoda	Malacostraca	Decapoda	Grapsidae	*Pachygrapsus*	*minutus*
17	Animalia	Arthropoda	Malacostraca	Decapoda	Hippolytidae	*Hippolyte*	
18	Animalia	Arthropoda	Malacostraca	Decapoda	Macrophthalmidae	*Macrophthalmus*	
19	Animalia	Arthropoda	Malacostraca	Decapoda	Majidae	*Micippa*	*platipes*
20	Animalia	Arthropoda	Malacostraca	Decapoda	Majidae	*Schizophrys*	
21	Animalia	Arthropoda	Malacostraca	Decapoda	Majidae	*Tiarinia*	
22	Animalia	Arthropoda	Malacostraca	Decapoda	Matutoidea	*Ashtoret*	
23	Animalia	Arthropoda	Malacostraca	Decapoda	Munididae		
24	Animalia	Arthropoda	Malacostraca	Decapoda	Palaemonidae	*Coralliocaris*	
25	Animalia	Arthropoda	Malacostraca	Decapoda	Penaeidae	*Melicertus*	
26	Animalia	Arthropoda	Malacostraca	Decapoda	Percnidae	*Percnon*	
27	Animalia	Arthropoda	Malacostraca	Decapoda	Pilumnidae		
28	Animalia	Arthropoda	Malacostraca	Decapoda	Portunidae	*Carupa*	
29	Animalia	Arthropoda	Malacostraca	Decapoda	Portunidae	*Charybdis*	
30	Animalia	Arthropoda	Malacostraca	Decapoda	Portunidae	*Libystes*	
31	Animalia	Arthropoda	Malacostraca	Decapoda	Portunidae	*Portunus*	*pelagicus*
32	Animalia	Arthropoda	Malacostraca	Decapoda	Portunidae	*Thalamita*	*admete*
33	Animalia	Arthropoda	Malacostraca	Decapoda	Tetraliidae	*Tetralia*	
34	Animalia	Arthropoda	Malacostraca	Decapoda	Trapeziidae	*Trapezia*	
35	Animalia	Arthropoda	Malacostraca	Decapoda	Varunidae		
36	Animalia	Arthropoda	Malacostraca	Decapoda	Xanthidae	*Atergatis*	
37	Animalia	Arthropoda	Malacostraca	Decapoda	Xanthidae	*Chlorodiella*	*laevissima*
38	Animalia	Arthropoda	Malacostraca	Decapoda	Xanthidae	*Cyclodius*	
39	Animalia	Arthropoda	Malacostraca	Decapoda	Xanthidae	*Etisus*	
40	Animalia	Arthropoda	Malacostraca	Decapoda	Xanthidae	*Liocarpilodes*	
41	Animalia	Arthropoda	Malacostraca	Decapoda	Xanthidae	*Liomera*	
42	Animalia	Arthropoda	Malacostraca	Decapoda	Xanthidae	*Macromedaeus*	
43	Animalia	Arthropoda	Malacostraca	Decapoda	Xanthidae	*Paraxanthias*	
44	Animalia	Arthropoda	Malacostraca	Decapoda	Xanthidae	*Pilodius*	*areolatus*
45	Animalia	Arthropoda	Malacostraca	Decapoda	Xanthidae	*Psaumis*	
46	Animalia	Arthropoda	Malacostraca	Stromatopoda	Nannosquillidae	*Pullosquilla*	
47	Animalia	Arthropoda	Maxillopoda	Lithoglyptida	Lithoglyptidae	*Auritoglyptes*	
48	Animalia	Bryozoa	Gymnolaemata	Cheilostomata	Beaniidae		
49	Animalia	Bryozoa	Gymnolaemata	Cheilostomata	Bictectiporidae		
50	Animalia	Bryozoa	Gymnolaemata	Cheilostomata	Smittinidae		
51	Animalia	Cnidaria	Hydrozoa	Leptothecata	Plumulariidae	*Plumularia*	
52	Animalia	Echinodermata	Ophiuroidea	Ophiurida	Amphiuridae	*Amphipholis*	
53	Animalia	Echinodermata	Ophiuroidea	Ophiurida	Ophiocomidae	*Ophiocoma*	

Supplementary Data 3 - 16S Ceph

Count	Kingdom	Phylum	Class	Order	Family	Genus	Species
1	Animalia	Arthropoda	Insecta	Coleoptera			
2	Animalia	Arthropoda	Insecta	Orthoptera	Acrididae		
3	Animalia	Echinodermata	Ophiuroidea	Ophiurida	Ophiothrichidae	*Macrophiothrix*	*caenosa*
4	Animalia	Mollusca	Bivalvia	Solemyoida	Solemyidae	*Solemya*	
5	Animalia	Mollusca	Cephalopoda	Octopoda	Octopodidae	*Abdopus*	
6	Animalia	Mollusca	Cephalopoda	Octopoda	Octopodidae	*Octopus*	*cyanea*
7	Animalia	Mollusca	Cephalopoda	Teuthida	Loliginidae	*Sepioteuthis*	
8	Animalia	Mollusca	Gastropoda	Caenogastropoda	Cerithiidae	*Cerithium*	*atromarginatum*
9	Animalia	Mollusca	Gastropoda	Caenogastropoda	Cerithiidae	*Cerithium*	*rostratum*
10	Animalia	Mollusca	Gastropoda	Caenogastropoda	Cerithiidae	*Clypeomorus*	
11	Animalia	Mollusca	Gastropoda	Caenogastropoda	Litiopidae	*Alaba*	
12	Animalia	Mollusca	Gastropoda	Caenogastropoda	Turritellidae		
13	Animalia	Mollusca	Gastropoda	Nudipleura			
14	Animalia	Mollusca	Gastropoda	Vetigastropoda	Haliotidae	*Haliotis*	*asinina*
15	Animalia	Mollusca	Gastropoda	Vetigastropoda	Trochidae	*Pseudostomatella*	
16	Animalia	Mollusca	Gastropoda	Vetigastropoda	Trochidae	*Rossiteria*	
17	Animalia	Mollusca	Gastropoda	Vetigastropoda	Trochidae	*Stomatella*	*impertusa*
18	Animalia	Mollusca	Gastropoda	Vetigastropoda	Trochidae	*Stomatia*	
19	Animalia	Mollusca	Gastropoda	Vetigastropoda	Turbinidae	*Turbo (Marmarostoma)*	

Supplementary Data 3 - 23S

Count	Kingdom	Phylum	Class	Order	Family	Genus	Species
1	Chromista	Myzozoa	Dinophyceae	Suessiales	Symbiodiniaceae	*Symbiodinium*	clade D
2	Chromista	Myzozoa	Dinophyceae	Suessiales	Symbiodiniaceae	*Symbiodinium*	clade B
3	Chromista	Myzozoa	Dinophyceae	Suessiales	Symbiodiniaceae	*Symbiodinium*	clade A
4	Chromista	Myzozoa	Dinophyceae	Suessiales	Symbiodiniaceae	*Symbiodinium*	clade C
5	Chromista	Myzozoa	Dinophyceae	Suessiales	Symbiodiniaceae	*Symbiodinium*	clade G
6	Chromista	Myzozoa	Dinophyceae	Suessiales	Symbiodiniaceae	*Symbiodinium*	clade H
7	Chromista	Myzozoa	Dinophyceae	Suessiales	Symbiodiniaceae	*Symbiodinium*	clade F

Supplementary Data 3 - trnL

Count	Kingdom	Phylum	Class	Order	Family	Genus	Species
1	Plantae	Streptophyta	Equisetopsida	Apiales	Apiaceae		
2	Plantae	Streptophyta	Equisetopsida	Asterales	Asteraceae		
3	Plantae	Streptophyta	Equisetopsida	Solanales	Solanaceae		
4	Plantae	Streptophyta	Equisetopsida	Caryophyllales	Amaranthaceae		
5	Plantae	Streptophyta	Equisetopsida	Cucurbitales	Cucurbitaceae		
6	Plantae	Streptophyta	Equisetopsida	Fabales	Fabaceae		
7	Plantae	Streptophyta	Equisetopsida	Brassicales	Caricaceae		
8	Plantae	Streptophyta	Equisetopsida	Sapindales	Rutaceae		
9	Plantae	Streptophyta	Equisetopsida	Poales	Poaceae		
10	Plantae	Streptophyta		Magnoliales	Magnoliaceae		

Supplementary Data 3- Prokaryotes

OTUNumber	Abundance	repSeqName	OTUConTaxonomy
Otu0001	45176	FTP55_1101_18143_2721_(reversed)	Bacteria(100);Proteobacteria(100);Alphaproteobacteria(100);unclassified(55);unclassified(55);unclassified(55);
Otu0002	71923	FTP55_1101_15943_2366_(reversed)	Bacteria(100);Proteobacteria(100);Alphaproteobacteria(100);Rhodobacterales(100);Rhodobacteraceae(100);unclassified(100);
Otu0003	9803	FTP55_1101_20199_2810_(reversed)	Bacteria(100);Proteobacteria(100);Gammaproteobacteria(100);Vibrionales(100);Vibrionaceae(100);unclassified(72);
Otu0004	78361	FTP55_1101_15313_2070_(reversed)	Bacteria(100);Bacteroidetes(100);Flavobacteriia(100);Flavobacteriales(100);Cryomorphaceae(100);Owenweeksia(100);
Otu0005	49244	FTP55_1101_15278_2260_(reversed)	Bacteria(100);Proteobacteria(100);Gammaproteobacteria(100);Oceanospirillales(100);Litoricolaceae(100);Litoricola(100);
Otu0006	3651	FTP55_1101_18449_2881_(reversed)	Bacteria(100);Proteobacteria(100);Alphaproteobacteria(100);Rickettsiales(99);SAR116_clade(99);unclassified(99);
Otu0007	5899	FTP55_1101_16830_3031_(reversed)	Bacteria(100);Proteobacteria(100);Alphaproteobacteria(100);Rickettsiales(100);SAR116_clade(100);unclassified(100);
Otu0008	10179	FTP55_1101_13057_2056_(reversed)	Bacteria(100);Proteobacteria(100);Gammaproteobacteria(100);Alteromonadales(100);Alteromonadaceae(100);Alteromonas(89);
Otu0009	7759	FTP55_1101_19067_2743_(reversed)	Bacteria(100);Bacteroidetes(100);Flavobacteriia(100);Flavobacteriales(100);Flavobacteriaceae(100);NS5_marine_group(100);
Otu0010	1725	FTP55_1101_14639_2893_(reversed)	Bacteria(100);Bacteroidetes(100);Flavobacteriia(100);Flavobacteriales(100);Flavobacteriaceae(100);NS5_marine_group(100);
Otu0011	10018	FTP55_1101_18956_2291_(reversed)	Bacteria(100);Proteobacteria(100);Gammaproteobacteria(100);Oceanospirillales(100);SAR86_clade(100);unclassified(100);
Otu0012	2173	FTP55_1101_19270_12716_(reversed)	Bacteria(100);Proteobacteria(100);Alphaproteobacteria(100);Rickettsiales(100);SAR116_clade(100);unclassified(100);
Otu0013	4993	FTP55_1101_18280_2152_(reversed)	Bacteria(100);Bacteroidetes(100);Flavobacteriia(100);Flavobacteriales(100);Flavobacteriaceae(100);NS5_marine_group(99);
Otu0014	2705	FTP55_1101_14645_3152_(reversed)	Bacteria(100);Bacteroidetes(100);Flavobacteriia(100);Flavobacteriales(100);Flavobacteriaceae(100);NS5_marine_group(100);
Otu0015	1613	FTP55_1101_14773_3284__(reversed)	Bacteria(100);Proteobacteria(100);Gammaproteobacteria(100);Oceanospirillales(100);Oceanospirillaceae(100);Oleibacter(99);
Otu0016	442	FTP55_1102_12013_9321__(reversed)	Bacteria(100);Proteobacteria(100);Alphaproteobacteria(100);Rhodobacterales(100);Rhodobacteraceae(100);unclassified(71);
Otu0017	2039	FTP55_1101_19339_4436_(reversed)	Bacteria(100);Proteobacteria(100);Alphaproteobacteria(100);OCS116_clade(100);unclassified(100);unclassified(100);
Otu0018	1696	FTP55_1101_7183_6749__(reversed)	Bacteria(100);Proteobacteria(100);Gammaproteobacteria(100);Oceanospirillales(100);Oceanospirillaceae(100);Oleibacter(99);
Otu0019	3846	FTP55_1101_13990_4046_(reversed)	Bacteria(100);Bacteroidetes(100);Flavobacteriia(100);Flavobacteriales(100);NS9_marine_group(100);unclassified(100);
Otu0020	5755	FTP55_1101_17029_2092_(reversed)	Bacteria(100);Proteobacteria(100);Gammaproteobacteria(100);Alteromonadales(100);Alteromonadaceae(100);Glaciecola(90);
Otu0021	455	FTP55_1101_18350_4332_(reversed)	Bacteria(100);Proteobacteria(100);Alphaproteobacteria(100);unclassified(61);unclassified(61);unclassified(61);
Otu0022	773	FTP55_1101_16825_2568_(reversed)	Bacteria(100);Proteobacteria(100);Alphaproteobacteria(100);Rickettsiales(100);SAR116_clade(100);unclassified(100);
Otu0023	970	FTP55_1101_7148_7390_(reversed)	Archaea(100);Euryarchaeota(100);Thermoplasmata(100);Thermoplasmatales(100);Marine_Group_II(100);unclassified(100);
Otu0024	2836	FTP55_1101_17847_3091_(reversed)	Bacteria(100);Proteobacteria(100);Gammaproteobacteria(100);Cellvibrionales(100);Halieaceae(100);OM60(NOR5)_clade(100);
Otu0025	268	FTP55_1101_17482_4815_(reversed)	Bacteria(100);Proteobacteria(100);Gammaproteobacteria(100);Oceanospirillales(100);Oleiphilaceae(98);Oleiphilus(98);
Otu0026	1070	FTP55_1101_11759_10918_(reversed)	Bacteria(100);Bacteroidetes(100);Flavobacteriia(100);Flavobacteriales(100);NS9_marine_group(100);unclassified(100);
Otu0027	1095	FTP55_1101_10957_2864_(reversed)	Bacteria(100);Bacteroidetes(100);Flavobacteriia(100);Flavobacteriales(100);Flavobacteriaceae(100);NS4_marine_group(98);
Otu0028	320	FTP55_1101_8969_7680_(reversed)	Bacteria(100);Proteobacteria(100);Alphaproteobacteria(100);Rhodobacterales(100);Rhodobacteraceae(100);unclassified(99);
Otu0029	665	FTP55_1101_14997_5128__(reversed)	Bacteria(100);Bacteroidetes(100);Flavobacteriia(100);Flavobacteriales(100);NS9_marine_group(93);unclassified(93);
Otu0030	539	FTP55_1101_18102_2180_(reversed)	Bacteria(100);Proteobacteria(100);Alphaproteobacteria(100);Rickettsiales(100);SAR116_clade(100);Candidatus_Puniceispirillum(83);
Otu0031	472	FTP55_1101_12206_11773_(reversed)	Bacteria(100);Proteobacteria(100);Alphaproteobacteria(100);Rickettsiales(100);SAR116_clade(100);unclassified(100);
Otu0032	1805	FTP55_1101_20734_2687_(reversed)	Bacteria(100);Bacteroidetes(100);Flavobacteriia(100);Flavobacteriales(100);Flavobacteriaceae(100);Formosa(99);
Otu0033	112	FTP55_1101_12343_16976_(reversed)	Bacteria(100);Proteobacteria(100);Alphaproteobacteria(100);Rickettsiales(100);SAR116_clade(100);unclassified(100);
Otu0034	290	FTP55_1101_24371_5736_(reversed)	Bacteria(100);Proteobacteria(100);Alphaproteobacteria(100);unclassified(63);unclassified(63);unclassified(63);
Otu0035	146	FTP55_1101_22828_20866_(reversed)	Bacteria(100);Proteobacteria(100);Alphaproteobacteria(100);Rickettsiales(100);SAR116_clade(100);unclassified(100);
Otu0036	243	FTP55_1101_13226_2190_(reversed)	Bacteria(100);Proteobacteria(100);Gammaproteobacteria(100);Cellvibrionales(100);Halieaceae(100);OM60(NOR5)_clade(100);
Otu0037	1321	FTP55_1101_20381_2581_(reversed)	Bacteria(100);Proteobacteria(100);Gammaproteobacteria(100);Oceanospirillales(100);Litoricolaceae(100);Litoricola(100);
Otu0038	486	FTP55_1101_17857_2193_(reversed)	Bacteria(100);Proteobacteria(100);Alphaproteobacteria(100);Rhodobacterales(100);Rhodobacteraceae(100);unclassified(100);
Otu0039	558	FTP55_1101_19029_5863_(reversed)	Bacteria(100);Bacteroidetes(100);Flavobacteriia(100);Flavobacteriales(100);Flavobacteriaceae(100);NS5_marine_group(99);
Otu0040	62	FTP55_101_16092_21768__(reversed)	Bacteria(100);Proteobacteria(100);Alphaproteobacteria(100);Rickettsiales(100);SAR116_clade(100);unclassified(100);
Otu0041	60	FTP55_1102_22354_3149_(reversed)	Bacteria(100);Proteobacteria(100);Alphaproteobacteria(100);Rhodobacterales(100);Rhodobacteraceae(100);unclassified(95);
Otu0042	137	FTP55_1101_10624_13132__(reversed)	Bacteria(100);Proteobacteria(100);Gammaproteobacteria(100);Vibrionales(99);Vibrionaceae(99);unclassified(95);
Otu0043	181	FTP55_1101_7711_22958_(reversed)	Bacteria(100);Proteobacteria(100);Alphaproteobacteria(100);Rhodobacterales(100);Rhodobacteraceae(100);Shimia(80);
Otu0044	46	FTP55_1101_7196_6519_(reversed)	Bacteria(100);Proteobacteria(100);Gammaproteobacteria(100);Vibrionales(100);Vibrionaceae(100);Photobacterium(96);
Otu0045	146	FTP55_1101_11742_6851_(reversed)	Bacteria(100);Bacteroidetes(100);Flavobacteriia(100);Flavobacteriales(100);Flavobacteriaceae(100);NS5_marine_group(100);
Otu0046	41	FTP55_1101_12083_4107__(reversed)	Bacteria(100);Bacteroidetes(100);Flavobacteriia(100);Flavobacteriales(100);Flavobacteriaceae(100);NS5_marine_group(100);
Otu0047	120	FTP55_1101_19565_8985_(reversed)	Bacteria(100);Proteobacteria(100);Gammaproteobacteria(100);Oceanospirillales(100);SAR86_clade(100);unclassified(100);
Otu0048	412	FTP55_1101_19863_3266_(reversed)	Bacteria(100);Bacteroidetes(100);Flavobacteriia(100);Flavobacteriales(100);NS9_marine_group(100);unclassified(100);
Otu0049	122	FTP55_1101_20864_5559_(reversed)	Bacteria(100);Bacteroidetes(100);Flavobacteriia(100);Flavobacteriales(100);Flavobacteriaceae(100);NS2b_marine_group(100);
Otu0050	301	FTP55_1101_13796_10812_(reversed)	Bacteria(100);Proteobacteria(100);Alphaproteobacteria(100);Rickettsiales(100);SAR116_clade(100);unclassified(100);
Otu0051	280	FTP55_1101_14953_2931_(reversed)	Bacteria(100);Bacteroidetes(100);Flavobacteriia(100);Flavobacteriales(100);Flavobacteriaceae(100);NS5_marine_group(100);
Otu0052	111	FTP55_1101_7343_14096_(reversed)	Bacteria(100);Proteobacteria(100);Alphaproteobacteria(100);unclassified(68);unclassified(68);unclassified(68);
Otu0053	19	FTP55_1102_16432_10746_(reversed)	Bacteria(100);Proteobacteria(100);Alphaproteobacteria(100);Rickettsiales(100);SAR116_clade(100);unclassified(100);
Otu0054	149	FTP55_1101_26237_6683_(reversed)	Bacteria(100);Proteobacteria(100);Epsilonproteobacteria(100);Campylobacterales(100);Campylobacteraceae(100);Arcobacter(100);
Otu0055	36	FTP55_1102_26983_14194_(reversed)	Bacteria(100);Bacteroidetes(100);Flavobacteriia(100);Flavobacteriales(100);Cryomorphaceae(75);Owenweeksia(75);
Otu0056	136	FTP55_1101_15849_22487_(reversed)	Bacteria(100);Bacteroidetes(100);Flavobacteriia(100);Flavobacteriales(100);Flavobacteriaceae(100);unclassified(100);
Otu0057	276	FTP55_1102_9026_5189_(reversed)	Bacteria(100);Proteobacteria(100);Gammaproteobacteria(100);Oceanospirillales(100);Oceanospirillaceae(100);Thalassolituus(100);
Otu0058	61	FTP55_1101_11615_4337__(reversed)	Bacteria(100);Proteobacteria(100);Epsilonproteobacteria(100);Campylobacterales(100);Campylobacteraceae(100);Arcobacter(100);
Otu0059	665	FTP55_1101_19249_3601_(reversed)	Bacteria(100);Proteobacteria(100);Alphaproteobacteria(100);Rhodobacterales(99);Rhodobacteraceae(99);unclassified(99);
Otu0060	209	FTP55_1101_8153_5341_(reversed)	Archaea(100);Euryarchaeota(100);Thermoplasmata(100);Thermoplasmatales(100);Marine_Group_II(100);unclassified(100);
Otu0061	246	FTP55_1101_9699_4837_(reversed)	Bacteria(100);Proteobacteria(100);Gammaproteobacteria(100);Oceanospirillales(100);Oceanospirillaceae(100);Litoribacillus(100);
Otu0062	16	FTP55_1101_14720_5125__(reversed)	Bacteria(100);Bacteroidetes(100);Flavobacteriia(100);Flavobacteriales(100);Flavobacteriaceae(100);Tenacibaculum(100);
Otu0063	172	FTP55_1101_21891_7504__(reversed)	Bacteria(100);Proteobacteria(100);Deltaproteobacteria(100);Bdellovibrionales(100);Bdellovibrionaceae(100);OM27_clade(100);
Otu0064	32	FTP55_1101_22614_13572__(reversed)	Bacteria(100);Proteobacteria(100);Alphaproteobacteria(100);Rickettsiales(100);S25-593(100);unclassified(100);
Otu0065	531	FTP55_1101_8706_7471_(reversed)	Bacteria(100);Proteobacteria(100);Gammaproteobacteria(100);Oceanospirillales(100);SAR86_clade(100);unclassified(100);
Otu0066	45	FTP55_1101_13949_11758_(reversed)	Bacteria(100);Proteobacteria(100);Gammaproteobacteria(100);Alteromonadales(100);Colwelliaceae(100);Thalassotalea(100);
Otu0067	99	FTP55_1101_11896_9451_(reversed)	Bacteria(100);Proteobacteria(100);Alphaproteobacteria(100);Rhodospirillales(100);Rhodospirillaceae(100);AEGEAN-169_marine_group(100);
Otu0068	48	FTP55_1101_14424_13462_(reversed)	Bacteria(100);Bacteroidetes(100);Flavobacteriia(100);Flavobacteriales(100);Flavobacteriaceae(100);Tenacibaculum(75);
Otu0069	229	FTP55_1102_9475_7254_(reversed)	Bacteria(100);Bacteroidetes(100);Flavobacteriia(100);Flavobacteriales(100);Flavobacteriaceae(100);NS5_marine_group(100);
Otu0070	227	FTP55_1101_6010_8191_(reversed)	Bacteria(100);Proteobacteria(100);Gammaproteobacteria(100);Oceanospirillales(100);SAR86_clade(100);unclassified(100);
Otu0071	124	FTP55_1102_22889_8475_(reversed)	Bacteria(100);Proteobacteria(100);Gammaproteobacteria(100);Alteromonadales(100);Pseudoalteromonadaceae(100);Pseudoalteromonas(100);
Otu0072	264	FTP55_1101_16060_7148_(reversed)	Bacteria(100);Actinobacteria(100);Acidimicrobiia(100);Acidimicrobiales(100);OM1_clade(100);Candidatus_Actinomarina(100);
Otu0073	15	FTP55_101_5848_10463__(reversed)	Bacteria(100);Proteobacteria(100);Epsilonproteobacteria(100);Campylobacterales(100);Campylobacteraceae(100);Arcobacter(100);
Otu0074	21	FTP55_1101_6035_14848__(reversed)	Bacteria(100);Proteobacteria(100);Alphaproteobacteria(100);unclassified(91);unclassified(91);unclassified(91);
Otu0075	70	FTP55_1101_11070_7908_(reversed)	Bacteria(100);Bacteroidetes(100);Flavobacteriia(100);Flavobacteriales(100);Flavobacteriaceae(100);NS4_marine_group(100);
Otu0076	11	FTP55_1101_18300_12813__(reversed)	Bacteria(100);Tenericutes(100);Mollicutes(100);NB1-n(100);unclassified(100);unclassified(100);
Otu0077	32	FTP55_1102_11988_11769_(reversed)	Bacteria(100);Proteobacteria(100);Alphaproteobacteria(100);OCS116_clade(100);unclassified(100);unclassified(100);
Otu0078	421	FTP55_1101_10018_16676_(reversed)	Bacteria(100);Proteobacteria(100);Gammaproteobacteria(100);Cellvibrionales(100);Spongiibacteraceae(100);Spongiibacter(100);
Otu0079	58	FTP55_1101_18997_11221_(reversed)	Bacteria(100);Proteobacteria(100);Gammaproteobacteria(100);Alteromonadales(100);Alteromonadaceae(100);Aestuariibacter(100);
Otu0080	14	FTP55_101_25528_15080__(reversed)	Bacteria(100);Proteobacteria(100);Alphaproteobacteria(100);Rickettsiales(100);SAR116_clade(100);unclassified(100);
Otu0081	30	FTP55_1101_23159_11265_(reversed)	Bacteria(100);Proteobacteria(100);Gammaproteobacteria(100);Vibrionales(100);Vibrionaceae(100);Enterovibrio(70);
Otu0082	347	FTP55_1101_13929_3769_(reversed)	Bacteria(100);Bacteroidetes(100);Flavobacteriia(100);Flavobacteriales(100);Flavobacteriaceae(100);NS5_marine_group(100);
Otu0083	14	FTP55_1102_3799_16980__(reversed)	Bacteria(100);Bacteroidetes(100);Flavobacteriia(100);Flavobacteriales(100);Flavobacteriaceae(100);NS5_marine_group(100);
Otu0084	172	FTP55_1101_7545_18495_(reversed)	Bacteria(100);Proteobacteria(100);Gammaproteobacteria(100);Alteromonadales(99);Colwelliaceae(99);unclassified(99);
Otu0085	225	FTP55_1101_3322_9337_(reversed)	Bacteria(100);Proteobacteria(100);Alphaproteobacteria(100);unclassified(96);unclassified(96);unclassified(96);
Otu0086	10	FTP55_1101_5523_8568_(reversed)	Bacteria(100);Proteobacteria(100);Gammaproteobacteria(100);Oceanospirillales(100);Oceanospirillaceae(100);Marinomonas(100);
Otu0087	11	FTP55_1101_23232_10702__(reversed)	Bacteria(100);Proteobacteria(100);Gammaproteobacteria(100);Oceanospirillales(100);Hahellaceae(100);Endozoicomonas(100);
Otu0088	89	FTP55_1101_7444_6550__(reversed)	Archaea(100);Euryarchaeota(100);Thermoplasmata(100);Thermoplasmatales(100);Marine_Group_II(100);unclassified(100);
Otu0089	89	FTP55_1101_18890_10489_(reversed)	Bacteria(100);Proteobacteria(100);Gammaproteobacteria(100);Cellvibrionales(100);Porticoccaceae(100);SAR92_clade(100);
Otu0090	46	FTP55_1101_8205_7104_(reversed)	Bacteria(100);Proteobacteria(100);Gammaproteobacteria(100);Vibrionales(100);Vibrionaceae(100);Vibrio(90);
Otu0091	9	FTP55_1102_14870_10287__(reversed)	Bacteria(100);Proteobacteria(100);Alphaproteobacteria(100);Rhodobacterales(100);Rhodobacteraceae(100);unclassified(100);
Otu0092	66	FTP55_1101_13037_14463_(reversed)	Bacteria(100);Proteobacteria(100);Epsilonproteobacteria(100);Campylobacterales(100);Campylobacteraceae(100);Arcobacter(100);
Otu0093	33	FTP55_101_18907_24776__(reversed)	Bacteria(100);Proteobacteria(100);Epsilonproteobacteria(100);Campylobacterales(100);Helicobacteraceae(100);Sulfurovum(100);
Otu0094	101	FTP55_1101_17571_9900__(reversed)	Bacteria(100);Proteobacteria(100);Epsilonproteobacteria(100);Campylobacterales(100);Campylobacteraceae(100);Arcobacter(100);
Otu0095	6	FTP55_1101_3567_12996__(reversed)	Bacteria(100);Proteobacteria(100);Gammaproteobacteria(100);Alteromonadales(100);Alteromonadaceae(100);Agaribacter(67);
Otu0096	21	FTP55_1101_16630_10704__(reversed)	Bacteria(100);Proteobacteria(100);Gammaproteobacteria(100);Oceanospirillales(100);Oceanospirillaceae(100);Amphritea(100);
Otu0097	31	FTP55_1102_20056_6998__(reversed)	Bacteria(100);Proteobacteria(100);Alphaproteobacteria(100);Rhodobacterales(100);Rhodobacteraceae(100);unclassified(100);
Otu0098	649	FTP55_1102_15845_10475_(reversed)	Bacteria(100);Proteobacteria(100);Gammaproteobacteria(100);Oceanospirillales(100);SAR86_clade(100);unclassified(100);
Otu0099	89	FTP55_1101_17586_5606_(reversed)	Bacteria(100);Proteobacteria(100);Gammaproteobacteria(100);Vibrionales(100);Vibrionaceae(100);Photobacterium(100);
Otu0100	12	FTP55_1101_12589_5806__(reversed)	Bacteria(100);Proteobacteria(100);Gammaproteobacteria(100);Oceanospirillales(100);Oceanospirillaceae(100);Reinekea(100);
Otu0101	24	FTP55_1101_13004_9954_(reversed)	Bacteria(100);Proteobacteria(100);Gammaproteobacteria(100);Oceanospirillales(100);Oceanospirillaceae(100);Pseudospirillum(100);
Otu0102	142	FTP55_1101_18728_5094_(reversed)	Bacteria(100);Proteobacteria(100);Gammaproteobacteria(100);Oceanospirillales(100);Oceanospirillaceae(100);unclassified(97);
Otu0103	7	FTP55_1101_15088_21233_(reversed)	Bacteria(100);Proteobacteria(100);Alphaproteobacteria(100);Rhodobacterales(100);Rhodobacteraceae(100);unclassified(100);
Otu0104	10	FTP55_1101_17649_16220__(reversed)	Bacteria(100);Proteobacteria(100);Alphaproteobacteria(100);Rhodobacterales(100);Rhodobacteraceae(100);unclassified(100);
Otu0105	56	FTP55_1101_22693_7333_(reversed)	Bacteria(100);Proteobacteria(100);Epsilonproteobacteria(100);Campylobacterales(100);Campylobacteraceae(100);Arcobacter(100);
Otu0106	9	FTP55_1101_22614_21082__(reversed)	Bacteria(100);Proteobacteria(100);unclassified(100);unclassified(100);unclassified(100);unclassified(100);

Otu0107	398	FTP55_1101_17595_4676_(reversed)	Bacteria(100);Proteobacteria(100);Alphaproteobacteria(100);SAR11_clade(100);Surface_1(100);Candidatus_Pelagibacter(95);
Otu0108	23	FTP55_1101_10033_10702__(reversed)	Bacteria(100);Proteobacteria(100);Alphaproteobacteria(100);Rhodobacterales(100);Rhodobacteraceae(100);unclassified(100);
Otu0109	44	FTP55_1102_10557_4973_(reversed)	Bacteria(100);Proteobacteria(100);Betaproteobacteria(100);Methylophilales(100);Methylophilaceae(100);OM43_clade(100);
Otu0110	80	FTP55_1101_14323_8062_(reversed)	Bacteria(100);Proteobacteria(100);Gammaproteobacteria(100);Order_Incertae_Sedis(100);Family_Incertae_Sedis(100);Marinicella(100);
Otu0111	16	FTP55_1101_16222_6026_(reversed)	Bacteria(100);Tenericutes(100);Mollicutes(100);NB1-n(100);unclassified(100);unclassified(100);
Otu0112	7	FTP55_1101_9951_9189__(reversed)	Bacteria(100);Bacteroidetes(100);Flavobacteriia(100);Flavobacteriales(100);Cryomorphaceae(100);Owenweeksia(100);
Otu0113	4	FTP55_1102_15552_2242__(reversed)	Bacteria(100);Verrucomicrobia(100);Verrucomicrobiae(100);Verrucomicrobiales(100);DEV007(100);unclassified(100);
Otu0114	23	FTP55_1101_8687_11094_(reversed)	Bacteria(100);Proteobacteria(100);Epsilonproteobacteria(100);Campylobacterales(100);Campylobacteraceae(100);Arcobacter(100);
Otu0115	6	FTP55_1101_4967_20956__(reversed)	Bacteria(100);Proteobacteria(100);Alphaproteobacteria(100);Rhodobacterales(100);Rhodobacteraceae(100);unclassified(100);
Otu0116	7	FTP55_1102_7137_4581__(reversed)	Bacteria(100);Proteobacteria(100);Gammaproteobacteria(100);unclassified(72);unclassified(72);unclassified(72);
Otu0117	4	FTP55_1102_18095_6869__(reversed)	Bacteria(100);Proteobacteria(100);Deltaproteobacteria(100);Bdellovibrionales(100);Bacteriovoracaceae(100);unclassified(100);
Otu0118	4	FTP55_1101_17572_11222__(reversed)	Bacteria(100);Proteobacteria(100);Gammaproteobacteria(100);Thiotrichales(100);Francisellaceae(100);Francisella(100);
Otu0119	4	FTP55_1101_5906_8653__(reversed)	Bacteria(100);Proteobacteria(100);Gammaproteobacteria(100);Alteromonadales(100);Alteromonadaceae(100);unclassified(100);
Otu0120	62	FTP55_1101_10413_8445_(reversed)	Bacteria(100);Bacteroidetes(100);Flavobacteriia(100);Flavobacteriales(100);unclassified(100);unclassified(100);
Otu0121	14	FTP55_1101_26541_6944__(reversed)	Bacteria(100);Proteobacteria(100);Alphaproteobacteria(100);Rhodospirillales(100);Rhodospirillaceae(100);AEGEAN-169_marine_group(100);
Otu0122	5	FTP55_1101_13105_9049__(reversed)	Bacteria(100);Tenericutes(100);Mollicutes(100);NB1-n(100);unclassified(100);unclassified(100);
Otu0123	34	FTP55_1101_5599_10035__(reversed)	Bacteria(100);Bacteroidetes(100);Cytophagia(100);Cytophagales(100);Flammeovirgaceae(100);Marinoscillum(100);
Otu0124	51	FTP55_1101_12929_5158_(reversed)	Bacteria(100);Proteobacteria(100);Alphaproteobacteria(100);SAR11_clade(100);Surface_4(100);unclassified(100);
Otu0125	12	FTP55_1101_25683_17724__(reversed)	Bacteria(100);Proteobacteria(100);Gammaproteobacteria(100);Vibrionales(100);Vibrionaceae(100);unclassified(100);
Otu0126	37	FTP55_1101_23683_14875_(reversed)	Bacteria(100);Gracilibacteria(100);unclassified(100);unclassified(100);unclassified(100);unclassified(100);
Otu0127	16	FTP55_1101_10690_3690__(reversed)	Bacteria(100);Bacteroidetes(100);Flavobacteriia(100);Flavobacteriales(100);Flavobacteriaceae(100);Aquimarina(100);
Otu0128	37	FTP55_1101_8280_8375_(reversed)	Bacteria(100);Bacteroidetes(100);Flavobacteriia(100);Flavobacteriales(100);Cryomorphaceae(100);Owenweeksia(100);
Otu0129	106	FTP55_1101_16544_7075_(reversed)	Bacteria(100);Proteobacteria(100);Gammaproteobacteria(100);Oceanospirillales(100);SAR86_clade(99);unclassified(99);
Otu0130	9	FTP55_101_10483_14862__(reversed)	Bacteria(100);Proteobacteria(100);Deltaproteobacteria(100);Bdellovibrionales(100);Bacteriovoracaceae(100);Halobacteriovorax(100);
Otu0131	4	FTP55_1102_8162_15100__(reversed)	Bacteria(100);Proteobacteria(100);Gammaproteobacteria(100);Alteromonadales(100);Colwelliaceae(100);Thalassotalea(100);
Otu0132	16	FTP55_1101_13598_27702__(reversed)	Bacteria(100);unclassified(100);unclassified(100);unclassified(100);unclassified(100);unclassified(100);
Otu0133	22	FTP55_1102_21131_2756__(reversed)	Bacteria(100);Bacteroidetes(100);Sphingobacteriia(100);Sphingobacteriales(100);Saprospiraceae(100);unclassified(100);
Otu0134	88	FTP55_1101_6699_14586_(reversed)	Bacteria(100);Bacteroidetes(100);unclassified(77);unclassified(77);unclassified(77);unclassified(77);
Otu0135	9	FTP55_1101_18915_17664__(reversed)	Bacteria(100);Bacteroidetes(100);Flavobacteriia(100);Flavobacteriales(100);Cryomorphaceae(100);Owenweeksia(100);
Otu0136	355	FTP55_1101_12117_5069_(reversed)	Bacteria(100);Proteobacteria(100);Alphaproteobacteria(100);Rhodospirillales(100);Rhodospirillaceae(100);AEGEAN-169_marine_group(100);
Otu0137	11	FTP55_1101_24409_16042__(reversed)	Bacteria(100);Proteobacteria(100);Deltaproteobacteria(100);Bdellovibrionales(100);Bacteriovoracaceae(100);Halobacteriovorax(73);
Otu0138	12	FTP55_1101_9103_13566__(reversed)	Bacteria(100);Proteobacteria(100);Alphaproteobacteria(100);unclassified(75);unclassified(75);unclassified(75);
Otu0139	29	FTP55_1101_18270_2060__(reversed)	Bacteria(100);Proteobacteria(100);Gammaproteobacteria(100);Oceanospirillales(100);OM182_clade(100);unclassified(100);
Otu0140	6	FTP55_102_11843_15488__(reversed)	Bacteria(100);Proteobacteria(100);Epsilonproteobacteria(100);Campylobacterales(100);Campylobacteraceae(100);Arcobacter(100);
Otu0141	10	FTP55_1102_8751_20236_(reversed)	Bacteria(100);Proteobacteria(100);Alphaproteobacteria(100);Rhodobacterales(100);Rhodobacteraceae(100);unclassified(100);
Otu0142	7	FTP55_101_27160_8290__(reversed)	Bacteria(100);Proteobacteria(100);unclassified(72);unclassified(72);unclassified(72);unclassified(72);
Otu0143	171	FTP55_1101_8398_4450_(reversed)	Bacteria(100);Bacteroidetes(100);Flavobacteriia(100);Flavobacteriales(100);Flavobacteriaceae(100);NS4_marine_group(100);
Otu0144	6	FTP55_1101_3976_14441_(reversed)	Bacteria(100);Proteobacteria(100);Alphaproteobacteria(100);Rhodospirillales(100);Rhodospirillaceae(100);Defluviicoccus(100);
Otu0145	86	FTP55_1101_6799_9318_(reversed)	Bacteria(100);Bacteroidetes(100);Flavobacteriia(100);Flavobacteriales(100);NS9_marine_group(100);unclassified(100);
Otu0146	5	FTP55_101_16355_16707__(reversed)	Bacteria(100);Proteobacteria(100);Gammaproteobacteria(100);Oceanospirillales(100);Oceanospirillaceae(100);Oceanospirillum(100);
Otu0147	12	FTP55_1101_4370_21114_(reversed)	Archaea(100);Euryarchaeota(100);Thermoplasmata(100);Thermoplasmatales(100);Marine_Group_II(100);unclassified(100);
Otu0148	12	FTP55_101_9270_19009__(reversed)	Bacteria(100);Proteobacteria(100);Epsilonproteobacteria(100);Campylobacterales(100);Campylobacteraceae(100);Arcobacter(100);
Otu0149	25	FTP55_1101_18750_8722__(reversed)	Bacteria(100);Proteobacteria(100);Gammaproteobacteria(100);Oceanospirillales(100);Oceanospirillaceae(100);Oleispira(100);
Otu0150	9	FTP55_1101_12346_18701__(reversed)	Bacteria(100);Bacteroidetes(100);Flavobacteriia(100);Flavobacteriales(100);NS9_marine_group(100);unclassified(100);
Otu0151	9	FTP55_1101_18135_4980__(reversed)	Bacteria(100);Bacteroidetes(100);Cytophagia(100);Cytophagales(100);Flammeovirgaceae(100);Reichenbachiella(100);
Otu0152	12	FTP55_1101_18041_7323__(reversed)	Bacteria(100);Proteobacteria(100);Gammaproteobacteria(100);Salinisphaerales(100);Salinisphaeraceae(100);Oceanococcus(100);
Otu0153	5	FTP55_1101_8572_11901__(reversed)	Bacteria(100);Proteobacteria(100);Alphaproteobacteria(100);Sphingomonadales(100);Erythrobacteraceae(100);Erythrobacter(100);
Otu0154	6	FTP55_1101_19244_22748__(reversed)	Bacteria(100);Proteobacteria(100);Gammaproteobacteria(100);unclassified(100);unclassified(100);unclassified(100);
Otu0155	11	FTP55_1101_12398_3775__(reversed)	Bacteria(100);Proteobacteria(100);Alphaproteobacteria(100);Rickettsiales(100);SAR116_clade(100);unclassified(100);
Otu0156	153	FTP55_1101_13182_2920_(reversed)	Bacteria(100);Marinimicrobia_(SAR406_clade)(100);unclassified(100);unclassified(100);unclassified(100);unclassified(100);
Otu0157	102	FTP55_1101_20755_11184_(reversed)	Bacteria(100);Proteobacteria(100);Alphaproteobacteria(100);Rickettsiales(100);SAR116_clade(100);unclassified(100);
Otu0158	9	FTP55_1101_15162_13623__(reversed)	Archaea(100);Euryarchaeota(100);Thermoplasmata(100);Thermoplasmatales(100);Marine_Group_II(100);unclassified(100);
Otu0159	5	FTP55_1101_11493_9911_(reversed)	Bacteria(100);Bacteroidetes(100);Flavobacteriia(100);Flavobacteriales(100);Flavobacteriaceae(100);Winogradskyella(100);
Otu0160	4	FTP55_1102_22696_11154__(reversed)	Bacteria(100);Tenericutes(100);Mollicutes(100);NB1-n(100);unclassified(100);unclassified(100);
Otu0161	11	FTP55_1101_19195_2702__(reversed)	Bacteria(100);Proteobacteria(100);Gammaproteobacteria(100);Thiotrichales(82);Thiotrichaceae(82);Thiothrix(82);
Otu0162	523	FTP55_1101_12956_5188_(reversed)	Bacteria(100);Proteobacteria(100);Betaproteobacteria(100);Methylophilales(100);Methylophilaceae(100);OM43_clade(100);
Otu0163	83	FTP55_1101_8944_3436_(reversed)	Bacteria(100);Proteobacteria(100);Alphaproteobacteria(100);unclassified(100);unclassified(100);unclassified(100);
Otu0164	5	FTP55_1101_13790_21110__(reversed)	Bacteria(100);Proteobacteria(100);Gammaproteobacteria(100);Oceanospirillales(100);Alcanivoracaceae(100);Kangiella(100);
Otu0165	4	FTP55_1101_21726_6149__(reversed)	Bacteria(100);Proteobacteria(100);Deltaproteobacteria(100);Desulfobacterales(100);Desulfobacteraceae(100);Desulforegula(100);
Otu0166	7	FTP55_1101_16291_14387__(reversed)	Bacteria(100);Proteobacteria(100);Gammaproteobacteria(100);Salinisphaerales(100);Salinisphaeraceae(100);Oceanococcus(100);
Otu0167	4	FTP55_1101_12523_12492__(reversed)	Bacteria(100);Proteobacteria(100);Alphaproteobacteria(100);Rhodobacterales(100);Rhodobacteraceae(100);unclassified(100);
Otu0168	22	FTP55_1102_16631_6691_(reversed)	Bacteria(100);Firmicutes(100);Clostridia(100);Clostridiales(100);Family_XII(100);Fusibacter(100);
Otu0169	11	FTP55_1101_21810_13993__(reversed)	Bacteria(100);Proteobacteria(100);Gammaproteobacteria(100);unclassified(100);unclassified(100);unclassified(100);
Otu0170	4	FTP55_1101_19692_22588__(reversed)	Bacteria(100);Proteobacteria(100);unclassified(100);unclassified(100);unclassified(100);unclassified(100);
Otu0171	5	FTP55_1101_20571_6662__(reversed)	Bacteria(100);Proteobacteria(100);unclassified(100);unclassified(100);unclassified(100);unclassified(100);
Otu0172	4	FTP55_1102_24790_11426__(reversed)	Bacteria(100);Bacteroidetes(100);Flavobacteriia(100);Flavobacteriales(100);NS9_marine_group(100);unclassified(100);
Otu0173	15	FTP55_1101_24914_14984_(reversed)	Bacteria(100);Bacteroidetes(100);unclassified(100);unclassified(100);unclassified(100);unclassified(100);
Otu0174	86	FTP55_1101_5159_6600_(reversed)	Bacteria(100);Proteobacteria(100);Epsilonproteobacteria(100);Campylobacterales(100);Campylobacteraceae(100);Arcobacter(100);
Otu0175	5	FTP55_1101_20166_16253__(reversed)	Bacteria(100);Proteobacteria(100);Alphaproteobacteria(100);Rickettsiales(100);SAR116_clade(100);unclassified(100);
Otu0176	9	FTP55_1101_21527_4330__(reversed)	Bacteria(100);Bacteroidetes(100);Flavobacteriia(100);Flavobacteriales(100);Flavobacteriaceae(100);unclassified(100);
Otu0177	128	FTP55_1101_10371_3388_(reversed)	Bacteria(100);Proteobacteria(100);Alphaproteobacteria(100);SAR11_clade(100);Surface_2(99);unclassified(99);
Otu0178	7	FTP55_1102_28049_18350__(reversed)	Bacteria(100);Proteobacteria(100);Deltaproteobacteria(100);Bdellovibrionales(100);Bdellovibrionaceae(100);OM27_clade(100);
Otu0179	6	FTP55_1102_18024_16266__(reversed)	Bacteria(100);Proteobacteria(100);Alphaproteobacteria(100);Rhodobacterales(100);Rhodobacteraceae(100);unclassified(100);
Otu0180	4	FTP55_1102_9543_24674__(reversed)	Bacteria(100);Proteobacteria(100);Alphaproteobacteria(100);Rickettsiales(100);SAR116_clade(100);unclassified(100);
Otu0181	2	FTP55_1101_22123_7258__(reversed)	Bacteria(100);unclassified(100);unclassified(100);unclassified(100);unclassified(100);unclassified(100);
Otu0182	2	FTP55_1101_22193_4386__(reversed)	Bacteria(100);Tenericutes(100);Mollicutes(100);Mollicutes_RF9(100);unclassified(100);unclassified(100);
Otu0183	2	FTP55_1101_22346_10988_(reversed)	Bacteria(100);Proteobacteria(100);Alphaproteobacteria(100);DB1-14(100);unclassified(100);unclassified(100);
Otu0184	2	FTP55_1101_22376_4718__(reversed)	Bacteria(100);Proteobacteria(100);unclassified(100);unclassified(100);unclassified(100);unclassified(100);
Otu0185	2	FTP55_1101_22110_23539_(reversed)	Bacteria(100);Proteobacteria(100);Deltaproteobacteria(100);Desulfobacterales(100);Desulfobulbaceae(100);unclassified(100);
Otu0186	2	FTP55_1101_22641_17699__(reversed)	Bacteria(100);unclassified(100);unclassified(100);unclassified(100);unclassified(100);unclassified(100);
Otu0187	2	FTP55_1101_22692_23753__(reversed)	Bacteria(100);Bacteroidetes(100);Flavobacteriia(100);Flavobacteriales(100);Flavobacteriaceae(100);unclassified(100);
Otu0188	2	FTP55_1101_23373_9561__(reversed)	Bacteria(100);Proteobacteria(100);Alphaproteobacteria(100);Sneathiellales(100);Sneathiellaceae(100);Sneathiella(100);
Otu0189	58	FTP55_1101_17710_6589_(reversed)	Bacteria(100);Proteobacteria(100);Gammaproteobacteria(100);KI89A_clade(100);unclassified(100);unclassified(100);
Otu0190	2	FTP55_1101_22023_13241__(reversed)	Bacteria(100);Proteobacteria(100);Gammaproteobacteria(100);unclassified(100);unclassified(100);unclassified(100);
Otu0191	2	FTP55_1101_23989_5459_(reversed)	Bacteria(100);Verrucomicrobia(100);Opitutae(100);MB11C04_marine_group(100);unclassified(100);unclassified(100);
Otu0192	2	FTP55_1101_10902_23984__(reversed)	Bacteria(100);Proteobacteria(100);Alphaproteobacteria(100);unclassified(100);unclassified(100);unclassified(100);
Otu0193	2	FTP55_1101_24316_12452__(reversed)	Bacteria(100);Proteobacteria(100);unclassified(100);unclassified(100);unclassified(100);unclassified(100);
Otu0194	2	FTP55_1101_21936_19105__(reversed)	Bacteria(100);Proteobacteria(100);Epsilonproteobacteria(100);Campylobacterales(100);Campylobacteraceae(100);Arcobacter(100);
Otu0195	2	FTP55_1101_24633_14670__(reversed)	Bacteria(100);Bacteroidetes(100);unclassified(100);unclassified(100);unclassified(100);unclassified(100);
Otu0196	2	FTP55_1101_2487_11226_(reversed)	Bacteria(100);unclassified(100);unclassified(100);unclassified(100);unclassified(100);unclassified(100);
Otu0197	2	FTP55_1101_25144_14401__(reversed)	Bacteria(100);Proteobacteria(100);Alphaproteobacteria(100);unclassified(100);unclassified(100);unclassified(100);
Otu0198	2	FTP55_1101_25448_6193__(reversed)	Bacteria(100);Proteobacteria(100);Gammaproteobacteria(100);Oceanospirillales(100);Oceanospirillaceae(100);Reinekea(100);
Otu0199	2	FTP55_1101_25451_14336_(reversed)	Bacteria(100);Proteobacteria(100);Epsilonproteobacteria(100);Campylobacterales(100);Campylobacteraceae(100);Arcobacter(100);
Otu0200	2	FTP55_1101_25687_13726__(reversed)	Bacteria(100);Proteobacteria(100);Gammaproteobacteria(100);Oceanospirillales(100);unclassified(100);unclassified(100);
Otu0201	2	FTP55_1101_21105_13477__(reversed)	Bacteria(100);Bacteroidetes(100);Flavobacteriia(100);Flavobacteriales(100);Cryomorphaceae(100);Fluviicola(100);
Otu0202	2	FTP55_1101_26322_24325__(reversed)	Bacteria(100);unclassified(100);unclassified(100);unclassified(100);unclassified(100);unclassified(100);
Otu0203	2	FTP55_1101_26962_23239__(reversed)	Bacteria(100);Proteobacteria(100);Alphaproteobacteria(100);Rhodobacterales(100);Rhodobacteraceae(100);unclassified(100);
Otu0204	2	FTP55_1101_11087_11644__(reversed)	Bacteria(100);Bacteroidetes(100);Flavobacteriia(100);Flavobacteriales(100);Flavobacteriaceae(100);unclassified(100);
Otu0205	39	FTP55_1102_21135_22201_(reversed)	Bacteria(100);Proteobacteria(100);Gammaproteobacteria(100);Oceanospirillales(100);unclassified(100);unclassified(100);
Otu0206	2	FTP55_1101_11199_15038_(reversed)	Bacteria(100);unclassified(100);unclassified(100);unclassified(100);unclassified(100);unclassified(100);
Otu0207	2	FTP55_1101_27755_9306__(reversed)	Bacteria(100);Proteobacteria(100);Gammaproteobacteria(100);Cellvibrionales(100);Porticoccaceae(100);SAR92_clade(100);
Otu0208	2	FTP55_1101_28447_16844__(reversed)	Bacteria(100);Proteobacteria(100);Gammaproteobacteria(100);Oceanospirillales(100);Litoricolaceae(100);Litoricola(100);
Otu0209	2	FTP55_1101_28496_11808__(reversed)	Bacteria(100);Fusobacteria(100);Fusobacteriia(100);Fusobacteriales(100);Fusobacteriaceae(100);Fusobacterium(100);
Otu0210	2	FTP55_1101_3083_16182__(reversed)	Bacteria(100);Proteobacteria(100);unclassified(100);unclassified(100);unclassified(100);unclassified(100);
Otu0211	2	FTP55_1101_10662_17152__(reversed)	Bacteria(100);Bacteroidetes(100);unclassified(100);unclassified(100);unclassified(100);unclassified(100);
Otu0212	2	FTP55_1101_20579_24818__(reversed)	Bacteria(100);Proteobacteria(100);Alphaproteobacteria(100);Rickettsiales(100);SAR116_clade(100);unclassified(100);
Otu0213	2	FTP55_1101_20565_21111__(reversed)	Bacteria(100);Proteobacteria(100);Gammaproteobacteria(100);Oceanospirillales(100);Alcanivoracaceae(100);Kangiella(100);
Otu0214	2	FTP55_1101_4365_18275__(reversed)	Bacteria(100);Proteobacteria(100);Alphaproteobacteria(100);Rickettsiales(100);unclassified(100);unclassified(100);

Otu0215	2	FTP55_1101_11217_8851__(reversed)	Bacteria(100);Bacteroidetes(100);unclassified(100);unclassified(100);unclassified(100);unclassified(100);
Otu0216	2	FTP55_1101_4673_18632__(reversed)	Bacteria(100);Proteobacteria(100);Deltaproteobacteria(100);SAR324_clade(Marine_group_B)(100);unclassified(100);unclassified(100);
Otu0217	2	FTP55_1101_4715_14416__(reversed)	Bacteria(100);Proteobacteria(100);Deltaproteobacteria(100);Myxococcales(100);unclassified(100);unclassified(100);
Otu0218	2	FTP55_1101_4839_17172__(reversed)	Bacteria(100);Proteobacteria(100);Gammaproteobacteria(100);Pseudomonadales(100);Moraxellaceae(100);Moraxella(100);
Otu0219	2	FTP55_1101_4842_10528__(reversed)	Bacteria(100);Bacteroidetes(100);unclassified(100);unclassified(100);unclassified(100);unclassified(100);
Otu0220	2	FTP55_1101_20428_15271__(reversed)	Bacteria(100);Proteobacteria(100);Gammaproteobacteria(100);Oceanospirillales(100);Oceanospirillaceae(100);Marinobacterium(100);
Otu0221	4	FTP55_1101_20281_6864__(reversed)	Bacteria(100);Proteobacteria(100);Gammaproteobacteria(100);unclassified(100);unclassified(100);unclassified(100);
Otu0222	43	FTP55_1101_23604_12552_(reversed)	Bacteria(100);Cyanobacteria(100);Cyanobacteria(100);SubsectionI(100);FamilyI(100);Prochlorococcus(100);
Otu0223	2	FTP55_1101_14483_19156__(reversed)	Bacteria(100);Bacteroidetes(100);unclassified(100);unclassified(100);unclassified(100);unclassified(100);
Otu0224	2	FTP55_1101_19825_14629__(reversed)	Bacteria(100);Bacteroidetes(100);Flavobacteriia(100);Flavobacteriales(100);Cryomorphaceae(100);Owenweeksia(100);
Otu0225	2	FTP55_1101_5444_5871__(reversed)	Bacteria(100);Proteobacteria(100);Gammaproteobacteria(100);Alteromonadales(100);unclassified(100);unclassified(100);
Otu0226	2	FTP55_1101_5491_10272__(reversed)	Bacteria(100);Proteobacteria(100);Alphaproteobacteria(100);Rhodospirillales(100);Rhodospirillaceae(100);Defluviicoccus(100);
Otu0227	2	FTP55_1101_11326_21584__(reversed)	Bacteria(100);Firmicutes(100);Clostridia(100);Clostridiales(100);unclassified(100);unclassified(100);
Otu0228	2	FTP55_1101_5736_11661__(reversed)	Bacteria(100);unclassified(100);unclassified(100);unclassified(100);unclassified(100);unclassified(100);
Otu0229	2	FTP55_102_18735_21743__(reversed)	Bacteria(100);Bacteroidetes(100);Flavobacteriia(100);Flavobacteriales(100);Cryomorphaceae(100);Owenweeksia(100);
Otu0230	2	FTP55_1101_11339_23580_(reversed)	Bacteria(100);Proteobacteria(100);Deltaproteobacteria(100);Desulfobacterales(100);Desulfobulbaceae(100);Desulfopila(100);
Otu0231	2	FTP55_1101_6069_17581__(reversed)	Bacteria(100);Bacteroidetes(100);unclassified(100);unclassified(100);unclassified(100);unclassified(100);
Otu0232	2	FTP55_1101_6148_7391__(reversed)	Bacteria(100);Proteobacteria(100);unclassified(100);unclassified(100);unclassified(100);unclassified(100);
Otu0233	4	FTP55_1101_6347_7584__(reversed)	Bacteria(100);Bacteroidetes(100);Flavobacteriia(100);Flavobacteriales(100);Flavobacteriaceae(100);NS2b_marine_group(100);
Otu0234	2	FTP55_1101_6711_14777__(reversed)	Bacteria(100);Proteobacteria(100);Gammaproteobacteria(100);NKB5(100);unclassified(100);unclassified(100);
Otu0235	2	FTP55_1101_6731_15967__(reversed)	Bacteria(100);Bacteroidetes(100);Flavobacteriia(100);Flavobacteriales(100);Flavobacteriaceae(100);NS5_marine_group(100);
Otu0236	2	FTP55_1101_6866_6786__(reversed)	Bacteria(100);Bacteroidetes(100);Bacteroidia(100);Bacteroidia_Incertae_Sedis(100);Draconibacteriaceae(100);Draconibacterium(100);
Otu0237	2	FTP55_1101_19514_28088__(reversed)	Bacteria(100);Proteobacteria(100);unclassified(100);unclassified(100);unclassified(100);unclassified(100);
Otu0238	2	FTP55_1101_6892_24645__(reversed)	Bacteria(100);Proteobacteria(100);Alphaproteobacteria(100);Rhodobacterales(100);Rhodobacteraceae(100);unclassified(100);
Otu0239	311	FTP55_1101_23489_5178_(reversed)	Bacteria(100);Cyanobacteria(100);Cyanobacteria(100);SubsectionI(100);FamilyI(100);Synechococcus(100);
Otu0240	2	FTP55_1101_7010_17744_(reversed)	Bacteria(100);Proteobacteria(100);Alphaproteobacteria(100);unclassified(100);unclassified(100);unclassified(100);
Otu0241	2	FTP55_1101_7133_22770_(reversed)	Bacteria(100);Bacteroidetes(100);Flavobacteriia(100);Flavobacteriales(100);Cryomorphaceae(100);Owenweeksia(100);
Otu0242	2	FTP55_1101_7323_24916__(reversed)	Bacteria(100);Lentisphaerae(100);Lentisphaeria(100);Lentisphaerales(100);Lentisphaeraceae(100);Lentisphaera(100);
Otu0243	2	FTP55_102_11966_14846__(reversed)	Bacteria(100);Bacteroidetes(100);unclassified(100);unclassified(100);unclassified(100);unclassified(100);
Otu0244	2	FTP55_1101_7829_18093__(reversed)	Bacteria(100);Proteobacteria(100);Gammaproteobacteria(100);Oceanospirillales(100);unclassified(100);unclassified(100);
Otu0245	2	FTP55_102_11680_6327__(reversed)	Bacteria(100);Proteobacteria(100);Gammaproteobacteria(100);Oceanospirillales(100);Oceanospirillaceae(100);unclassified(100);
Otu0246	29	FTP55_1101_21102_25059__(reversed)	Bacteria(100);Bacteroidetes(100);Bacteroidia(100);Bacteroidia_Incertae_Sedis(100);Prolixibacteraceae(100);Prolixibacter(100);
Otu0247	2	FTP55_1101_8073_23009__(reversed)	Bacteria(100);Proteobacteria(100);Gammaproteobacteria(100);Cellvibrionales(100);Cellvibrionaceae(100);unclassified(100);
Otu0248	2	FTP55_1101_8327_4950__(reversed)	Bacteria(100);Bacteroidetes(100);Flavobacteriia(100);Flavobacteriales(100);Cryomorphaceae(100);Owenweeksia(100);
Otu0249	50	FTP55_1101_20928_5110_(reversed)	Bacteria(100);Proteobacteria(100);Alphaproteobacteria(100);Rickettsiales(100);SAR116_clade(100);unclassified(100);
Otu0250	2	FTP55_1101_8511_20508__(reversed)	Bacteria(100);Proteobacteria(100);Deltaproteobacteria(100);Bdellovibrionales(100);Bacteriovoracaceae(100);Peredibacter(100);
Otu0251	25	FTP55_1101_10278_3093_(reversed)	Bacteria(100);Cyanobacteria(100);unclassified(100);unclassified(100);unclassified(100);unclassified(100);
Otu0252	2	FTP55_102_10880_16811__(reversed)	Bacteria(100);Proteobacteria(100);Deltaproteobacteria(100);Desulfuromonadales(100);GR-WP33-58(100);unclassified(100);
Otu0253	2	FTP55_1101_8768_12295__(reversed)	Bacteria(100);Proteobacteria(100);Gammaproteobacteria(100);Cellvibrionales(100);Halieaceae(100);OM60(NOR5)_clade(100);
Otu0254	2	FTP55_1101_19279_7219__(reversed)	Bacteria(100);Bacteroidetes(100);Flavobacteriia(100);Flavobacteriales(100);Flavobacteriaceae(100);unclassified(100);
Otu0255	2	FTP55_1101_19217_25639__(reversed)	Bacteria(100);Bacteroidetes(100);unclassified(100);unclassified(100);unclassified(100);unclassified(100);
Otu0256	2	FTP55_1101_9292_3887__(reversed)	Bacteria(100);Tenericutes(100);Mollicutes(100);Acholeplasmatales(100);Acholeplasmataceae(100);Acholeplasma(100);
Otu0257	2	FTP55_1101_9355_14569__(reversed)	Bacteria(100);Proteobacteria(100);Alphaproteobacteria(100);unclassified(100);unclassified(100);unclassified(100);
Otu0258	2	FTP55_1101_9451_9193__(reversed)	Bacteria(100);Bacteroidetes(100);Bacteroidia(100);Bacteroidales(100);Marinilabiaceae(100);Marinifilum(100);
Otu0259	2	FTP55_101_9291_8595__(reversed)	Bacteria(100);Proteobacteria(100);Gammaproteobacteria(100);unclassified(100);unclassified(100);unclassified(100);
Otu0260	2	FTP55_1102_10067_16503__(reversed)	Bacteria(100);Bacteroidetes(100);Sphingobacteriia(100);Sphingobacteriales(100);NS11-12_marine_group(100);unclassified(100);
Otu0261	2	FTP55_1101_11482_20957__(reversed)	Bacteria(100);Proteobacteria(100);Gammaproteobacteria(100);unclassified(100);unclassified(100);unclassified(100);
Otu0262	2	FTP55_1102_10793_3192__(reversed)	Bacteria(100);Gracilibacteria(100);unclassified(100);unclassified(100);unclassified(100);unclassified(100);
Otu0263	26	FTP55_1101_12299_21356__(reversed)	Bacteria(100);Bacteroidetes(100);unclassified(100);unclassified(100);unclassified(100);unclassified(100);
Otu0264	2	FTP55_1101_19139_23564__(reversed)	Bacteria(100);Bacteroidetes(100);Cytophagia(100);Cytophagales(100);Flammeovirgaceae(100);Fabibacter(100);
Otu0265	2	FTP55_1102_12100_16095__(reversed)	Bacteria(100);Bacteroidetes(100);unclassified(100);unclassified(100);unclassified(100);unclassified(100);
Otu0266	2	FTP55_1102_12670_4833__(reversed)	Bacteria(100);Proteobacteria(100);Deltaproteobacteria(100);Bdellovibrionales(100);Bacteriovoracaceae(100);Halobacteriovorax(100);
Otu0267	2	FTP55_1101_19124_3451_(reversed)	Bacteria(100);Proteobacteria(100);Gammaproteobacteria(100);unclassified(100);unclassified(100);unclassified(100);
Otu0268	2	FTP55_101_4358_19686__(reversed)	Bacteria(100);Proteobacteria(100);Alphaproteobacteria(100);Rhodobacterales(100);Rhodobacteraceae(100);unclassified(100);
Otu0269	2	FTP55_1102_13711_6233__(reversed)	Bacteria(100);Bacteroidetes(100);Flavobacteriia(100);Flavobacteriales(100);Cryomorphaceae(100);Owenweeksia(100);
Otu0270	20	FTP55_1101_9621_14005_(reversed)	Bacteria(100);Proteobacteria(100);Gammaproteobacteria(100);Alteromonadales(100);Alteromonadaceae(100);unclassified(100);
Otu0271	2	FTP55_101_3688_12769__(reversed)	Bacteria(100);Proteobacteria(100);Alphaproteobacteria(100);Rickettsiales(100);SAR116_clade(100);unclassified(100);
Otu0272	2	FTP55_1102_14411_5703__(reversed)	Bacteria(100);Proteobacteria(100);Gammaproteobacteria(100);unclassified(100);unclassified(100);unclassified(100);
Otu0273	2	FTP55_101_29070_12518__(reversed)	Bacteria(100);Proteobacteria(100);Gammaproteobacteria(100);Oceanospirillales(100);Oceanospirillaceae(100);unclassified(100);
Otu0274	2	FTP55_1102_14481_11112__(reversed)	Bacteria(100);Bacteroidetes(100);unclassified(100);unclassified(100);unclassified(100);unclassified(100);
Otu0275	2	FTP55_1102_14522_3800__(reversed)	Bacteria(100);Lentisphaerae(100);Oligosphaeria(100);unclassified(100);unclassified(100);unclassified(100);
Otu0276	2	FTP55_1102_14589_10807__(reversed)	Bacteria(100);Bacteroidetes(100);Cytophagia(100);Cytophagales(100);Flammeovirgaceae(100);Marinoscillum(100);
Otu0277	2	FTP55_1102_14737_11859__(reversed)	Bacteria(100);Proteobacteria(100);Deltaproteobacteria(100);Oligoflexales(100);Oligoflexaceae(100);unclassified(100);
Otu0278	2	FTP55_1102_14752_4745__(reversed)	Bacteria(100);Proteobacteria(100);Alphaproteobacteria(100);Rickettsiales(100);SAR116_clade(100);unclassified(100);
Otu0279	2	FTP55_1102_14755_10181__(reversed)	Bacteria(100);Bacteroidetes(100);Flavobacteriia(100);Flavobacteriales(100);unclassified(100);unclassified(100);
Otu0280	2	FTP55_1102_16146_8771__(reversed)	Bacteria(100);Proteobacteria(100);Alphaproteobacteria(100);Rhodospirillales(100);Rhodospirillaceae(100);AEGEAN-169_marine_group(100);
Otu0281	2	FTP55_1102_16161_3737__(reversed)	Bacteria(100);Proteobacteria(100);Alphaproteobacteria(100);Rhodobacterales(100);Rhodobacteraceae(100);unclassified(100);
Otu0282	2	FTP55_1101_11526_14087__(reversed)	Bacteria(100);Bacteroidetes(100);unclassified(100);unclassified(100);unclassified(100);unclassified(100);
Otu0283	2	FTP55_1102_16509_20036__(reversed)	Bacteria(100);Proteobacteria(100);Gammaproteobacteria(100);Oceanospirillales(100);Oceanospirillaceae(100);Oceaniserpentilla(100);
Otu0284	2	FTP55_1101_18620_15716_(reversed)	Bacteria(100);Proteobacteria(100);Gammaproteobacteria(100);Alteromonadales(100);unclassified(100);unclassified(100);
Otu0285	2	FTP55_1102_16758_12898__(reversed)	Bacteria(100);Proteobacteria(100);Deltaproteobacteria(100);Desulfuromonadales(100);unclassified(100);unclassified(100);
Otu0286	2	FTP55_1102_17482_2776_(reversed)	Bacteria(100);Bacteroidetes(100);Flavobacteriia(100);Flavobacteriales(100);Flavobacteriaceae(100);unclassified(100);
Otu0287	2	FTP55_1102_18511_2178__(reversed)	Bacteria(100);Proteobacteria(100);Deltaproteobacteria(100);Bdellovibrionales(100);Bdellovibrionaceae(100);OM27_clade(100);
Otu0288	2	FTP55_1102_18911_5551__(reversed)	Bacteria(100);Proteobacteria(100);Gammaproteobacteria(100);unclassified(100);unclassified(100);unclassified(100);
Otu0289	19	FTP55_1101_11740_14102_(reversed)	Bacteria(100);Proteobacteria(100);Gammaproteobacteria(100);Oceanospirillales(100);Oceanospirillaceae(100);Marinomonas(100);
Otu0290	2	FTP55_101_24708_9146__(reversed)	Bacteria(100);Proteobacteria(100);Gammaproteobacteria(100);Alteromonadales(100);unclassified(100);unclassified(100);
Otu0291	59	FTP55_1101_22226_12011_(reversed)	Bacteria(100);Bacteroidetes(100);Flavobacteriia(100);Flavobacteriales(100);Flavobacteriaceae(100);NS4_marine_group(100);
Otu0292	2	FTP55_1102_20662_2432__(reversed)	Bacteria(100);Bacteroidetes(100);Flavobacteriia(100);Flavobacteriales(100);Cryomorphaceae(100);Owenweeksia(100);
Otu0293	35	FTP55_1101_9195_9715_(reversed)	Bacteria(100);Proteobacteria(100);Alphaproteobacteria(100);Rhodobacterales(100);Rhodobacteraceae(100);unclassified(100);
Otu0294	2	FTP55_1102_20957_12728__(reversed)	Bacteria(100);Proteobacteria(100);Alphaproteobacteria(100);Rhodobacterales(100);Rhodobacteraceae(100);unclassified(100);
Otu0295	2	FTP55_1102_20989_8298__(reversed)	Bacteria(100);Proteobacteria(100);Gammaproteobacteria(100);Oceanospirillales(100);Oceanospirillaceae(100);Neptuniibacter(100);
Otu0296	2	FTP55_1101_18492_7454__(reversed)	Bacteria(100);Firmicutes(100);Clostridia(100);Clostridiales(100);Lachnospiraceae(100);unclassified(100);
Otu0297	2	FTP55_1102_21879_19430__(reversed)	Bacteria(100);Bacteroidetes(100);Flavobacteriia(100);Flavobacteriales(100);NS9_marine_group(100);unclassified(100);
Otu0298	40	FTP55_1101_14650_28277__(reversed)	Bacteria(100);Bacteroidetes(100);Flavobacteriia(100);Flavobacteriales(100);Flavobacteriaceae(100);NS5_marine_group(100);
Otu0299	2	FTP55_1102_22444_23767_(reversed)	Bacteria(100);Firmicutes(100);Clostridia(100);Clostridiales(100);Family_XII(100);Fusibacter(100);
Otu0300	2	FTP55_1101_12105_16676_(reversed)	Bacteria(100);Proteobacteria(100);Gammaproteobacteria(100);Alteromonadales(100);Alteromonadaceae(100);unclassified(100);
Otu0301	2	FTP55_1102_25264_9549__(reversed)	Bacteria(100);Fusobacteria(100);Fusobacteriia(100);Fusobacteriales(100);Leptotrichiaceae(100);Sebaldella(100);
Otu0302	2	FTP55_1102_27547_11169__(reversed)	Bacteria(100);Tenericutes(100);Mollicutes(100);Entomoplasmatales(100);Entomoplasmatales_Incertae_Sedis(100);Candidatus_Hepatoplasma(100);
Otu0303	2	FTP55_1102_4779_7559__(reversed)	Bacteria(100);Marinimicrobia_(SAR406_clade)(100);unclassified(100);unclassified(100);unclassified(100);unclassified(100);
Otu0304	2	FTP55_1102_5136_12481__(reversed)	Bacteria(100);Firmicutes(100);Clostridia(100);Clostridiales(100);unclassified(100);unclassified(100);
Otu0305	2	FTP55_1102_5544_10564__(reversed)	Bacteria(100);Proteobacteria(100);Gammaproteobacteria(100);unclassified(100);unclassified(100);unclassified(100);
Otu0306	2	FTP55_1102_5765_12310_(reversed)	Bacteria(100);Bacteroidetes(100);Flavobacteriia(100);Flavobacteriales(100);Flavobacteriaceae(100);unclassified(100);
Otu0307	2	FTP55_101_23154_9627__(reversed)	Bacteria(100);Proteobacteria(100);Alphaproteobacteria(100);DB1-14(100);unclassified(100);unclassified(100);
Otu0308	2	FTP55_1102_7706_6870__(reversed)	Bacteria(100);Proteobacteria(100);unclassified(100);unclassified(100);unclassified(100);unclassified(100);
Otu0309	2	FTP55_1102_8401_13690__(reversed)	Bacteria(100);Proteobacteria(100);Epsilonproteobacteria(100);Campylobacterales(100);Campylobacteraceae(100);Arcobacter(100);
Otu0310	2	FTP55_1101_17873_15860__(reversed)	Bacteria(100);Proteobacteria(100);Gammaproteobacteria(100);unclassified(100);unclassified(100);unclassified(100);
Otu0311	2	FTP55_1101_17863_20458__(reversed)	Bacteria(100);Proteobacteria(100);Gammaproteobacteria(100);Oceanospirillales(100);Oceanospirillaceae(100);unclassified(100);
Otu0312	16	FTP55_1101_7666_6256__(reversed)	Bacteria(100);Proteobacteria(100);Gammaproteobacteria(100);unclassified(100);unclassified(100);unclassified(100);
Otu0313	2	FTP55_1102_9426_7285__(reversed)	Bacteria(100);Proteobacteria(100);Gammaproteobacteria(100);unclassified(100);unclassified(100);unclassified(100);
Otu0314	2	FTP55_1102_9499_5395_(reversed)	Bacteria(100);Proteobacteria(100);Alphaproteobacteria(100);Rhizobiales(100);Rhodobiaceae(100);Rhodobium(100);
Otu0315	2	FTP55_101_22416_15482__(reversed)	Bacteria(100);Proteobacteria(100);Epsilonproteobacteria(100);Campylobacterales(100);Campylobacteraceae(100);Arcobacter(100);
Otu0316	2	FTP55_1101_12237_9893__(reversed)	Bacteria(100);Verrucomicrobia(100);Verrucomicrobiae(100);Verrucomicrobiales(100);Verrucomicrobiaceae(100);Roseibacillus(100);
Otu0317	2	FTP55_1101_17726_16900__(reversed)	Bacteria(100);Bacteroidetes(100);Flavobacteriia(100);Flavobacteriales(100);unclassified(100);unclassified(100);
Otu0318	2	FTP55_1101_17576_11791__(reversed)	Bacteria(100);Proteobacteria(100);Alphaproteobacteria(100);unclassified(100);unclassified(100);unclassified(100);
Otu0319	28	FTP55_1101_17648_12999__(reversed)	Bacteria(100);Proteobacteria(100);Gammaproteobacteria(100);Order_Incertae_Sedis(100);Family_Incertae_Sedis(100);Marinicella(100);
Otu0320	22	FTP55_1101_15645_5410_(reversed)	Bacteria(100);Marinimicrobia_(SAR406_clade)(100);unclassified(100);unclassified(100);unclassified(100);unclassified(100);
Otu0321	2	FTP55_1101_12268_24133__(reversed)	Bacteria(100);Bacteroidetes(100);Flavobacteriia(100);Flavobacteriales(100);Cryomorphaceae(100);Owenweeksia(100);
Otu0322	2	FTP55_101_20400_14137__(reversed)	Bacteria(100);Proteobacteria(100);unclassified(100);unclassified(100);unclassified(100);unclassified(100);

Otu0323	2	FTP55_1101_17480_8125__(reversed)	Bacteria(100);Proteobacteria(100);Epsilonproteobacteria(100);unclassified(100);unclassified(100);unclassified(100);
Otu0324	3	FTP55_1101_15868_13586__(reversed)	Bacteria(100);Bacteroidetes(100);Flavobacteriia(100);Flavobacteriales(100);Flavobacteriaceae(100);unclassified(100);
Otu0325	2	FTP55_1101_17405_15543__(reversed)	Bacteria(100);Proteobacteria(100);Gammaproteobacteria(100);Vibrionales(100);Vibrionaceae(100);unclassified(100);
Otu0326	3	FTP55_1101_16446_9311__(reversed)	Bacteria(100);Proteobacteria(100);Alphaproteobacteria(100);SAR11_clade(100);Surface_1(100);unclassified(100);
Otu0327	3	FTP55_1101_16502_10331__(reversed)	Bacteria(100);Bacteroidetes(100);unclassified(100);unclassified(100);unclassified(100);unclassified(100);
Otu0328	2	FTP55_1101_17344_8044__(reversed)	Bacteria(100);Bacteroidetes(100);Flavobacteriia(100);Flavobacteriales(100);Flavobacteriaceae(100);NS5_marine_group(100);
Otu0329	2	FTP55_1101_12302_4675__(reversed)	Bacteria(100);Proteobacteria(100);Alphaproteobacteria(100);Rhodospirillales(100);Rhodospirillaceae(100);Novispirillum(100);
Otu0330	3	FTP55_1101_19442_10865__(reversed)	Bacteria(100);Proteobacteria(100);Deltaproteobacteria(100);Bdellovibrionales(100);Bdellovibrionaceae(100);Bdellovibrio(100);
Otu0331	2	FTP55_1101_17167_22020__(reversed)	Bacteria(100);Proteobacteria(100);Betaproteobacteria(100);unclassified(100);unclassified(100);unclassified(100);
Otu0332	3	FTP55_1101_19805_15220__(reversed)	Bacteria(100);unclassified(100);unclassified(100);unclassified(100);unclassified(100);unclassified(100);
Otu0333	3	FTP55_1101_19987_12172__(reversed)	Bacteria(100);Proteobacteria(100);Alphaproteobacteria(100);4-Org1-14(100);unclassified(100);unclassified(100);
Otu0334	3	FTP55_1101_21823_11572__(reversed)	Bacteria(100);Bacteroidetes(100);unclassified(100);unclassified(100);unclassified(100);unclassified(100);
Otu0335	3	FTP55_1101_22559_14853__(reversed)	Bacteria(100);Proteobacteria(100);Epsilonproteobacteria(100);Campylobacterales(100);Campylobacteraceae(100);Arcobacter(100);
Otu0336	3	FTP55_1101_22563_9110_(reversed)	Bacteria(100);Proteobacteria(100);Alphaproteobacteria(100);Kordiimonadales(100);unclassified(100);unclassified(100);
Otu0337	3	FTP55_1101_25037_9111__(reversed)	Bacteria(100);Bacteroidetes(100);Sphingobacteriia(100);Sphingobacteriales(100);Saprospiraceae(100);unclassified(100);
Otu0338	2	FTP55_1101_12492_27121__(reversed)	Bacteria(100);Proteobacteria(100);Epsilonproteobacteria(100);unclassified(100);unclassified(100);unclassified(100);
Otu0339	15	FTP55_1101_17979_25670__(reversed)	Bacteria(100);Verrucomicrobia(100);Verrucomicrobiae(100);Verrucomicrobiales(100);Verrucomicrobiaceae(100);Roseibacillus(100);
Otu0340	17	FTP55_1101_17379_2190_(reversed)	Bacteria(100);Proteobacteria(100);Gammaproteobacteria(100);Alteromonadales(100);Pseudoalteromonadaceae(100);Psychrosphaera(100);
Otu0341	3	FTP55_1101_27351_8450__(reversed)	Bacteria(100);Proteobacteria(100);Alphaproteobacteria(100);Rickettsiales(100);SAR116_clade(100);unclassified(100);
Otu0342	3	FTP55_1101_28108_10151__(reversed)	Bacteria(100);Firmicutes(100);Bacilli(100);Lactobacillales(100);Streptococcaceae(100);Streptococcus(100);
Otu0343	3	FTP55_1101_3373_14563__(reversed)	Bacteria(100);Proteobacteria(100);unclassified(100);unclassified(100);unclassified(100);unclassified(100);
Otu0344	2	FTP55_101_18811_24684__(reversed)	Bacteria(100);Proteobacteria(100);Alphaproteobacteria(100);Rickettsiales(100);unclassified(100);unclassified(100);
Otu0345	3	FTP55_1101_4970_6526__(reversed)	Bacteria(100);Bacteroidetes(100);Cytophagia(100);Cytophagales(100);unclassified(100);unclassified(100);
Otu0346	3	FTP55_1101_5434_13513__(reversed)	Bacteria(100);Proteobacteria(100);Gammaproteobacteria(100);Oceanospirillales(100);unclassified(100);unclassified(100);
Otu0347	3	FTP55_1101_6061_8793__(reversed)	Bacteria(100);Cyanobacteria(100);Cyanobacteria(100);unclassified(100);unclassified(100);unclassified(100);
Otu0348	3	FTP55_1101_7463_11935_(reversed)	Bacteria(100);Proteobacteria(100);Gammaproteobacteria(100);Oceanospirillales(100);Litoricolaceae(100);Litoricola(100);
Otu0349	2	FTP55_1101_16846_4431__(reversed)	Bacteria(100);Proteobacteria(100);SC3-20(100);unclassified(100);unclassified(100);unclassified(100);
Otu0350	2	FTP55_101_17862_9298__(reversed)	Bacteria(100);Bacteroidetes(100);unclassified(100);unclassified(100);unclassified(100);unclassified(100);
Otu0351	5	FTP55_1102_8492_5707_(reversed)	Bacteria(100);Bacteroidetes(100);Flavobacteriia(100);Flavobacteriales(100);Flavobacteriaceae(100);Winogradskyella(100);
Otu0352	2	FTP55_101_17212_14536__(reversed)	Bacteria(100);Bacteroidetes(100);Flavobacteriia(100);Flavobacteriales(100);Flavobacteriaceae(100);Aquibacter(100);
Otu0353	4	FTP55_102_13595_9468__(reversed)	Bacteria(100);Proteobacteria(100);Gammaproteobacteria(100);Cellvibrionales(100);Halieaceae(100);Pseudohaliea(100);
Otu0354	14	FTP55_1101_18796_5694__(reversed)	Bacteria(100);Proteobacteria(100);Gammaproteobacteria(100);Oceanospirillales(100);Alcanivoracaceae(100);Alcanivorax(100);
Otu0355	2	FTP55_101_17061_8022__(reversed)	Bacteria(100);Proteobacteria(100);Alphaproteobacteria(100);Rickettsiales(100);SAR116_clade(100);unclassified(100);
Otu0356	4	FTP55_1101_11848_4295__(reversed)	Bacteria(100);Proteobacteria(100);Deltaproteobacteria(100);Bdellovibrionales(100);Bacteriovoracaceae(100);Halobacteriovorax(100);
Otu0357	2	FTP55_1101_16803_2576__(reversed)	Bacteria(100);Proteobacteria(100);Gammaproteobacteria(100);unclassified(100);unclassified(100);unclassified(100);
Otu0358	2	FTP55_1101_16754_18066__(reversed)	Bacteria(100);Proteobacteria(100);Alphaproteobacteria(100);Rhodobacterales(100);Rhodobacteraceae(100);Labrenzia(100);
Otu0359	4	FTP55_1101_13524_2080__(reversed)	Bacteria(100);Bacteroidetes(100);Flavobacteriia(100);Flavobacteriales(100);Cryomorphaceae(100);Owenweeksia(100);
Otu0360	6	FTP55_1101_13632_24668_(reversed)	Bacteria(100);Bacteroidetes(100);unclassified(100);unclassified(100);unclassified(100);unclassified(100);
Otu0361	4	FTP55_1101_13675_3052__(reversed)	Bacteria(100);Proteobacteria(100);Gammaproteobacteria(100);unclassified(100);unclassified(100);unclassified(100);
Otu0362	4	FTP55_1101_13917_2262__(reversed)	Bacteria(100);Proteobacteria(100);Deltaproteobacteria(100);Desulfobacterales(100);Desulfobacteraceae(100);unclassified(100);
Otu0363	4	FTP55_1101_13937_15779__(reversed)	Bacteria(100);Lentisphaerae(100);Oligosphaeria(100);unclassified(100);unclassified(100);unclassified(100);
Otu0364	2	FTP55_101_16826_7692__(reversed)	Bacteria(100);Proteobacteria(100);Gammaproteobacteria(100);unclassified(100);unclassified(100);unclassified(100);
Otu0365	4	FTP55_1101_15866_10530__(reversed)	Bacteria(100);Proteobacteria(100);Gammaproteobacteria(100);Oceanospirillales(100);Oceanospirillaceae(100);unclassified(100);
Otu0366	2	FTP55_101_15912_10437__(reversed)	Bacteria(100);Proteobacteria(100);Deltaproteobacteria(100);Bdellovibrionales(100);Bdellovibrionaceae(100);OM27_clade(100);
Otu0367	15	FTP55_1101_17412_9310__(reversed)	Bacteria(100);Proteobacteria(100);Alphaproteobacteria(100);Rickettsiales(100);S25-593(100);unclassified(100);
Otu0368	4	FTP55_1101_19629_14903_(reversed)	Bacteria(100);Proteobacteria(100);Gammaproteobacteria(100);Oceanospirillales(100);Oleiphilaceae(100);Oleiphilus(100);
Otu0369	4	FTP55_1101_19655_9411_(reversed)	Bacteria(100);unclassified(100);unclassified(100);unclassified(100);unclassified(100);unclassified(100);
Otu0370	2	FTP55_1101_16632_17718_(reversed)	Bacteria(100);Proteobacteria(100);Gammaproteobacteria(100);Alteromonadales(100);Pseudoalteromonadaceae(100);Algicola(100);
Otu0371	4	FTP55_1101_20441_12228_(reversed)	Bacteria(100);Proteobacteria(100);Alphaproteobacteria(100);Rhodobacterales(100);Rhodobacteraceae(100);unclassified(100);
Otu0372	4	FTP55_1101_22978_11852__(reversed)	Bacteria(100);Bacteroidetes(100);Flavobacteriia(100);Flavobacteriales(100);Flavobacteriaceae(100);NS5_marine_group(100);
Otu0373	4	FTP55_1101_23100_9039_(reversed)	Bacteria(100);Bacteroidetes(100);Flavobacteriia(100);Flavobacteriales(100);Cryomorphaceae(100);Owenweeksia(100);
Otu0374	12	FTP55_1101_8031_5530_(reversed)	Bacteria(100);Bacteroidetes(100);Flavobacteriia(100);Flavobacteriales(100);Flavobacteriaceae(100);Aquibacter(100);
Otu0375	12	FTP55_1101_5135_20450__(reversed)	Bacteria(100);Marinimicrobia_(SAR406_clade)(100);unclassified(100);unclassified(100);unclassified(100);unclassified(100);
Otu0376	2	FTP55_1101_12547_14674__(reversed)	Bacteria(100);Proteobacteria(100);Deltaproteobacteria(100);Desulfovibrionales(100);Desulfovibrionaceae(100);Desulfovibrio(100);
Otu0377	4	FTP55_1101_23944_4555__(reversed)	Bacteria(100);Proteobacteria(100);Deltaproteobacteria(100);SAR324_clade(Marine_group_B)(100);unclassified(100);unclassified(100);
Otu0378	2	FTP55_1101_16458_18111_(reversed)	Bacteria(100);Proteobacteria(100);Gammaproteobacteria(100);unclassified(100);unclassified(100);unclassified(100);
Otu0379	4	FTP55_1101_25441_14823__(reversed)	Bacteria(100);Bacteroidetes(100);Sphingobacteriia(100);Sphingobacteriales(100);Saprospiraceae(100);unclassified(100);
Otu0380	4	FTP55_1101_26883_12135__(reversed)	Bacteria(100);unclassified(100);unclassified(100);unclassified(100);unclassified(100);unclassified(100);
Otu0381	2	FTP55_101_15118_26030__(reversed)	Bacteria(100);Proteobacteria(100);Gammaproteobacteria(100);Thiotrichales(100);Thiotrichaceae(100);unclassified(100);
Otu0382	4	FTP55_1101_3711_9253__(reversed)	Bacteria(100);Proteobacteria(100);Alphaproteobacteria(100);unclassified(100);unclassified(100);unclassified(100);
Otu0383	12	FTP55_1101_15132_8174__(reversed)	Bacteria(100);Gracilibacteria(100);unclassified(100);unclassified(100);unclassified(100);unclassified(100);
Otu0384	4	FTP55_1101_4495_7193__(reversed)	Bacteria(100);Proteobacteria(100);Alphaproteobacteria(100);DB1-14(100);unclassified(100);unclassified(100);
Otu0385	2	FTP55_101_14765_5997__(reversed)	Bacteria(100);Proteobacteria(100);Deltaproteobacteria(100);Desulfobacterales(100);Desulfobulbaceae(100);unclassified(100);
Otu0386	15	FTP55_1101_13740_13697__(reversed)	Bacteria(100);Proteobacteria(100);Gammaproteobacteria(100);unclassified(100);unclassified(100);unclassified(100);
Otu0387	12	FTP55_1101_12500_5137_(reversed)	Bacteria(100);Proteobacteria(100);Gammaproteobacteria(100);Oceanospirillales(100);SAR86_clade(100);unclassified(100);
Otu0388	2	FTP55_1101_16332_11081__(reversed)	Bacteria(100);Proteobacteria(100);Gammaproteobacteria(100);unclassified(100);unclassified(100);unclassified(100);
Otu0389	4	FTP55_1102_8909_10206__(reversed)	Bacteria(100);Bacteroidetes(100);Flavobacteriia(100);Flavobacteriales(100);Flavobacteriaceae(100);NS4_marine_group(100);
Otu0390	4	FTP55_1102_9040_7344__(reversed)	Bacteria(100);Proteobacteria(100);unclassified(100);unclassified(100);unclassified(100);unclassified(100);
Otu0391	2	FTP55_1101_16151_10482__(reversed)	Bacteria(100);Proteobacteria(100);Alphaproteobacteria(100);Rhodobacterales(100);Rhodobacteraceae(100);unclassified(100);
Otu0392	11	FTP55_1102_11792_3434__(reversed)	Bacteria(100);Proteobacteria(100);Gammaproteobacteria(100);Oceanospirillales(100);Oceanospirillaceae(100);Marinobacterium(100);
Otu0393	5	FTP55_1101_12323_7419__(reversed)	Bacteria(100);Proteobacteria(100);Gammaproteobacteria(100);unclassified(100);unclassified(100);unclassified(100);
Otu0394	5	FTP55_1101_12372_11814_(reversed)	Bacteria(100);Proteobacteria(100);Deltaproteobacteria(100);Oligoflexales(100);Oligoflexaceae(100);unclassified(100);
Otu0395	2	FTP55_1101_13164_8951_(reversed)	Bacteria(100);unclassified(100);unclassified(100);unclassified(100);unclassified(100);unclassified(100);
Otu0396	5	FTP55_1101_14338_22436__(reversed)	Bacteria(100);Proteobacteria(100);unclassified(100);unclassified(100);unclassified(100);unclassified(100);
Otu0397	5	FTP55_1101_19111_9134__(reversed)	Bacteria(100);Bacteroidetes(100);unclassified(100);unclassified(100);unclassified(100);unclassified(100);
Otu0398	5	FTP55_1101_22119_17373__(reversed)	Bacteria(100);Bacteroidetes(100);Flavobacteriia(100);Flavobacteriales(100);Cryomorphaceae(100);Fluviicola(100);
Otu0399	5	FTP55_1101_24439_6959_(reversed)	Bacteria(100);Bacteroidetes(100);unclassified(100);unclassified(100);unclassified(100);unclassified(100);
Otu0400	2	FTP55_101_10903_5724__(reversed)	Bacteria(100);Proteobacteria(100);Gammaproteobacteria(100);Cellvibrionales(100);Cellvibrionaceae(100);Simiduia(100);
Otu0401	5	FTP55_1102_18168_2785__(reversed)	Bacteria(100);Bacteroidetes(100);Bacteroidia(100);Bacteroidia_Incertae_Sedis(100);Draconibacteriaceae(100);Draconibacterium(100);
Otu0402	11	FTP55_101_12620_25473__(reversed)	Archaea(100);Euryarchaeota(100);Thermoplasmata(100);Thermoplasmatales(100);Marine_Group_II(100);unclassified(100);
Otu0403	11	FTP55_1101_18343_28778__(reversed)	Bacteria(100);Proteobacteria(100);Gammaproteobacteria(100);Oceanospirillales(100);Oceanospirillaceae(100);Litoribacillus(100);
Otu0404	6	FTP55_101_8445_8224__(reversed)	Bacteria(100);Proteobacteria(100);Epsilonproteobacteria(100);Campylobacterales(100);Campylobacteraceae(100);Arcobacter(100);
Otu0405	6	FTP55_1101_17101_25748__(reversed)	Bacteria(100);Bacteroidetes(100);Flavobacteriia(100);Flavobacteriales(100);NS7_marine_group(100);unclassified(100);
Otu0406	6	FTP55_1101_20848_4399__(reversed)	Bacteria(100);Proteobacteria(100);Gammaproteobacteria(100);unclassified(100);unclassified(100);unclassified(100);
Otu0407	6	FTP55_1101_21869_11920__(reversed)	Bacteria(100);Proteobacteria(100);Gammaproteobacteria(100);Alteromonadales(100);Pseudoalteromonadaceae(100);Algicola(100);
Otu0408	6	FTP55_1101_22474_4312_(reversed)	Bacteria(100);Bacteroidetes(100);unclassified(100);unclassified(100);unclassified(100);unclassified(100);
Otu0409	6	FTP55_1101_24187_5079__(reversed)	Bacteria(100);Bacteroidetes(100);Flavobacteriia(100);Flavobacteriales(100);Cryomorphaceae(100);Owenweeksia(100);
Otu0410	6	FTP55_1101_25972_7812__(reversed)	Bacteria(100);Proteobacteria(100);Gammaproteobacteria(100);Salinisphaerales(100);Salinisphaeraceae(100);ZD0417_marine_group(100);
Otu0411	6	FTP55_1101_26353_10014__(reversed)	Bacteria(100);Proteobacteria(100);Epsilonproteobacteria(100);Campylobacterales(100);Campylobacteraceae(100);Arcobacter(100);
Otu0412	2	FTP55_101_10673_9518__(reversed)	Bacteria(100);Proteobacteria(100);Gammaproteobacteria(100);Thiotrichales(100);Francisellaceae(100);Francisella(100);
Otu0413	2	FTP55_1101_15282_20822__(reversed)	Bacteria(100);Proteobacteria(100);Epsilonproteobacteria(100);Campylobacterales(100);Campylobacteraceae(100);Arcobacter(100);
Otu0414	6	FTP55_1102_11263_5438__(reversed)	Bacteria(100);Proteobacteria(100);Epsilonproteobacteria(100);Campylobacterales(100);Helicobacteraceae(100);Sulfurovum(100);
Otu0415	10	FTP55_1102_24758_10891__(reversed)	Bacteria(100);Proteobacteria(100);Gammaproteobacteria(100);Alteromonadales(100);Ferrimonadaceae(100);Ferrimonas(100);
Otu0416	6	FTP55_1102_15663_17759__(reversed)	Bacteria(100);Proteobacteria(100);Deltaproteobacteria(100);Bdellovibrionales(100);Bdellovibrionaceae(100);OM27_clade(100);
Otu0417	10	FTP55_1102_22329_14339_(reversed)	Bacteria(100);Proteobacteria(100);Gammaproteobacteria(100);NKB5(100);unclassified(100);unclassified(100);
Otu0418	2	FTP55_1101_15090_26781_(reversed)	Bacteria(100);Proteobacteria(100);Gammaproteobacteria(100);Oceanospirillales(100);unclassified(100);unclassified(100);
Otu0419	10	FTP55_1102_11941_9712__(reversed)	Bacteria(100);Bacteroidetes(100);unclassified(100);unclassified(100);unclassified(100);unclassified(100);
Otu0420	6	FTP55_1102_20412_11797__(reversed)	Bacteria(100);Proteobacteria(100);Gammaproteobacteria(100);Cellvibrionales(100);Cellvibrionaceae(100);Pseudomaricurvus(100);
Otu0421	2	FTP55_1101_14267_13671__(reversed)	Bacteria(100);Proteobacteria(100);Gammaproteobacteria(100);unclassified(100);unclassified(100);unclassified(100);
Otu0422	7	FTP55_101_11961_3035__(reversed)	Bacteria(100);Proteobacteria(100);Epsilonproteobacteria(100);Campylobacterales(100);Helicobacteraceae(100);Rs-M59_termite_group(100);
Otu0423	7	FTP55_101_6221_6244__(reversed)	Bacteria(100);Proteobacteria(100);Epsilonproteobacteria(100);Campylobacterales(100);Campylobacteraceae(100);Sulfurospirillum(100);
Otu0424	11	FTP55_1101_16269_10580__(reversed)	Bacteria(100);Proteobacteria(100);Gammaproteobacteria(100);Oceanospirillales(100);SAR86_clade(100);unclassified(100);
Otu0425	7	FTP55_1101_22317_10622__(reversed)	Bacteria(100);Firmicutes(100);Clostridia(100);Clostridiales(100);Family_XII(100);Fusibacter(100);
Otu0426	7	FTP55_1101_25222_12679__(reversed)	Bacteria(100);Proteobacteria(100);Alphaproteobacteria(100);Rickettsiales(100);SAR116_clade(100);unclassified(100);
Otu0427	7	FTP55_1101_26089_15281__(reversed)	Bacteria(100);Bacteroidetes(100);Bacteroidia(100);Bacteroidales(100);Marinilabiaceae(100);Marinifilum(100);
Otu0428	7	FTP55_1101_4642_11894__(reversed)	Bacteria(100);unclassified(100);unclassified(100);unclassified(100);unclassified(100);unclassified(100);
Otu0429	9	FTP55_1102_26567_15754__(reversed)	Bacteria(100);Proteobacteria(100);Alphaproteobacteria(100);SAR11_clade(100);unclassified(100);unclassified(100);
Otu0430	8	FTP55_101_13735_15466__(reversed)	Bacteria(100);Proteobacteria(100);Epsilonproteobacteria(100);Campylobacterales(100);Helicobacteraceae(100);Sulfurovum(100);

Otu0431	8	FTP55_1101_14576_8251_(reversed)	Bacteria(100);Proteobacteria(100);Alphaproteobacteria(100);Rhodobacterales(100);Rhodobacteraceae(100);unclassified(100);
Otu0432	8	FTP55_1101_20566_2331__(reversed)	Bacteria(100);Fusobacteria(100);Fusobacteriia(100);Fusobacteriales(100);Fusobacteriaceae(100);Cetobacterium(100);
Otu0433	10	FTP55_1101_17573_20759_(reversed)	Bacteria(100);Proteobacteria(100);Deltaproteobacteria(100);Desulfovibrionales(100);Desulfovibrionaceae(100);Desulfovibrio(100);
Otu0434	8	FTP55_1101_26614_18965_(reversed)	Bacteria(100);Proteobacteria(100);Gammaproteobacteria(100);Cellvibrionales(100);Cellvibrionaceae(100);unclassified(100);
Otu0435	8	FTP55_1101_7596_10787__(reversed)	Bacteria(100);Proteobacteria(100);Gammaproteobacteria(100);Alteromonadales(100);Alteromonadaceae(100);Marinobacter(100);
Otu0436	9	FTP55_1101_10579_12643_(reversed)	Bacteria(100);Proteobacteria(100);Alphaproteobacteria(100);Rhodospirillales(100);Rhodospirillaceae(100);AEGEAN-169_marine_group(100);
Otu0437	10	FTP55_102_9908_19722__(reversed)	Bacteria(100);Proteobacteria(100);Epsilonproteobacteria(100);Campylobacterales(100);Helicobacteraceae(100);Sulfurimonas(100);
Otu0438	2	FTP55_1101_15059_13708_(reversed)	Bacteria(100);Bacteroidetes(100);Flavobacteriia(100);Flavobacteriales(100);NS9_marine_group(100);unclassified(100);
Otu0439	9	FTP55_1101_16561_18036_(reversed)	Bacteria(100);Bacteroidetes(100);Flavobacteriia(100);Flavobacteriales(100);Flavobacteriaceae(100);Nonlabens(100);
Otu0440	9	FTP55_1101_18458_18934__(reversed)	Bacteria(100);Proteobacteria(100);Gammaproteobacteria(100);Alteromonadales(100);Pseudoalteromonadaceae(100);Algicola(100);
Otu0441	2	FTP55_1101_14792_10734__(reversed)	Bacteria(100);Proteobacteria(100);Epsilonproteobacteria(100);Campylobacterales(100);Helicobacteraceae(100);Sulfurovum(100);
Otu0442	17	FTP55_1101_23568_6348__(reversed)	Bacteria(100);Proteobacteria(100);Gammaproteobacteria(100);Oceanospirillales(100);Oceanospirillaceae(100);Neptuniibacter(100);
Otu0443	9	FTP55_1102_10110_15101__(reversed)	Bacteria(100);Bacteroidetes(100);Flavobacteriia(100);Flavobacteriales(100);Cryomorphaceae(100);Owenweeksia(100);
Otu0444	11	FTP55_1101_7376_5602_(reversed)	Bacteria(100);Proteobacteria(100);Epsilonproteobacteria(100);Campylobacterales(100);Campylobacteraceae(100);Arcobacter(100);
Otu0445	9	FTP55_1101_8234_11233__(reversed)	Bacteria(100);Proteobacteria(100);Deltaproteobacteria(100);Oligoflexales(100);Oligoflexaceae(100);unclassified(100);

Supplementary Data 4

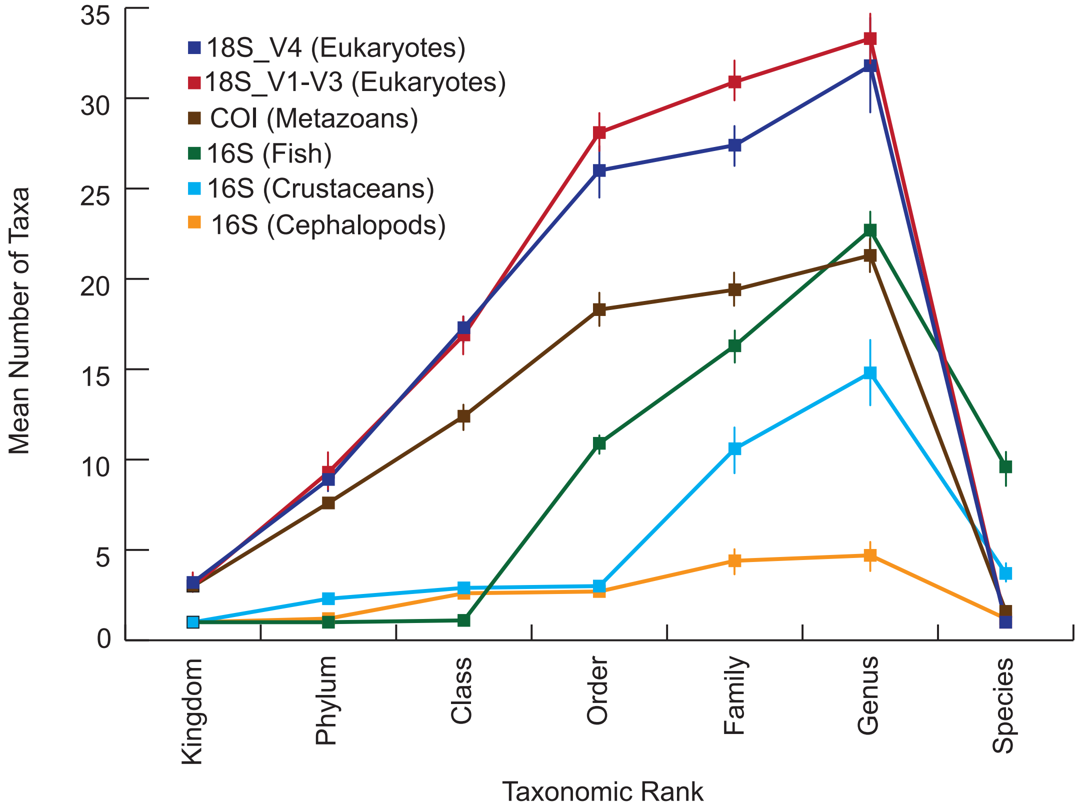

Line graph representing the mean number of eukaryotic taxa recorded at Coral Bay using eDNA. Coloured lines indicate the mean number of taxa identified for each taxonomic rank for six PCR assays that target eukaryotes. Sequences were normalised across each sample and assay to 2363 sequences.Vertical lines represent the standard error.

Supplementary Data 5

Rarefaction plots depicting the number of sequences recovered for each of the nine PCR assays that target eukaryotes and the relationship to the number of taxa (i.e. families or genera) recovered. Rarefaction lines are in red with 95% upper and lower confidence limits in grey.

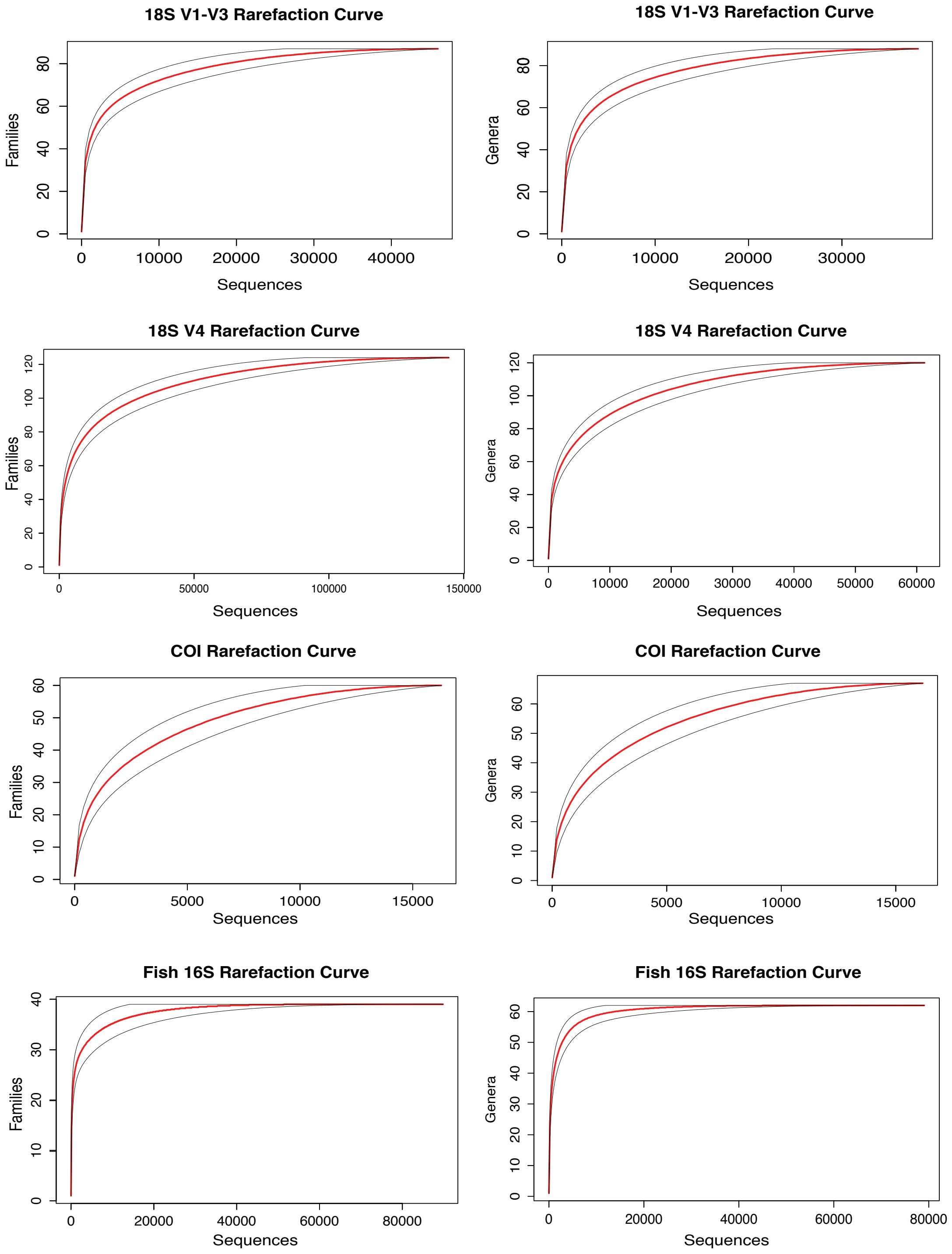

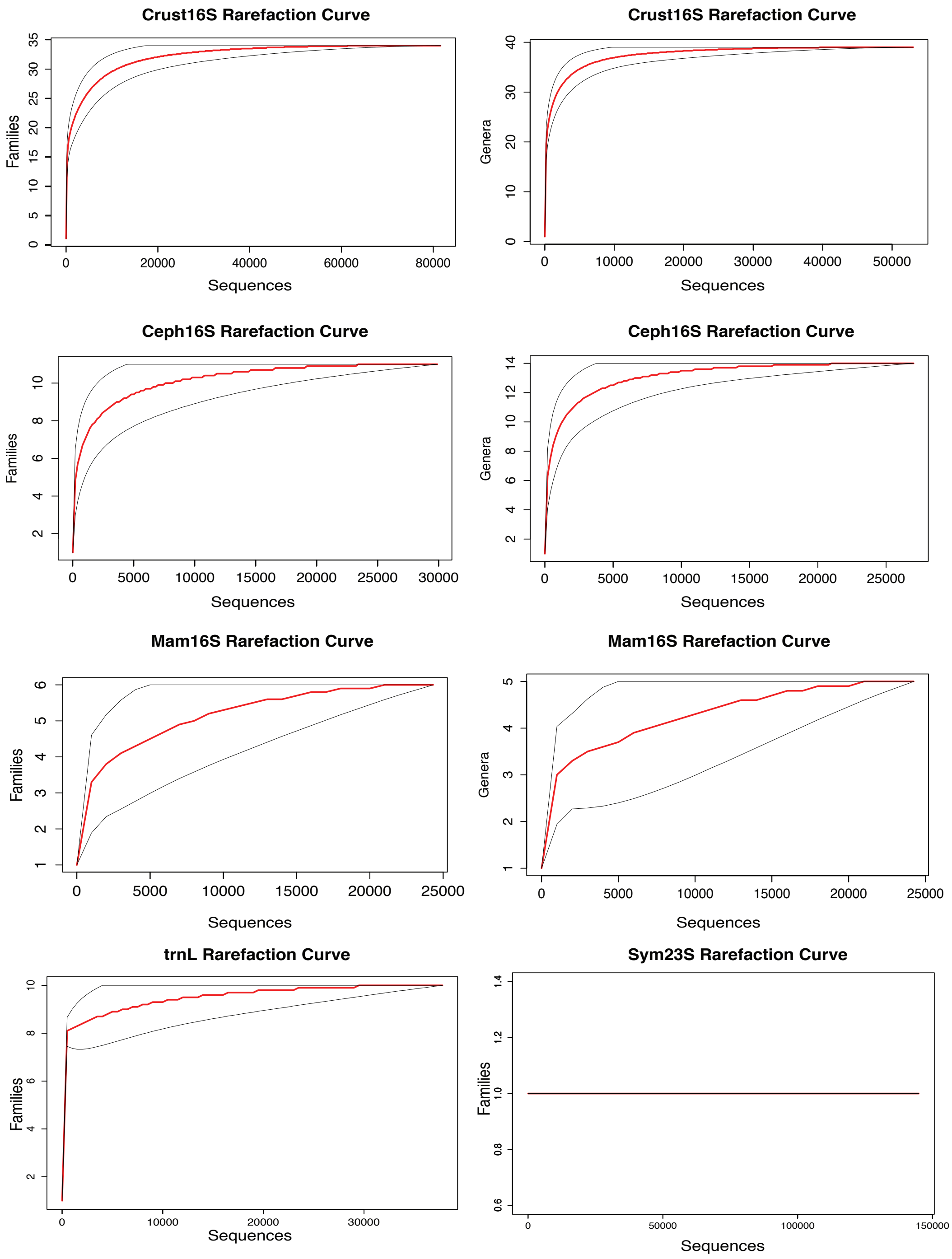
Crust16S Rarefaction Curve
Families
0 5 10 15 20 25 30 35
0 20000 40000 60000 80000
Sequences
Crust16S Rarefaction Curve
Genera
0 10 20 30 40
0 10000 20000 30000 40000 50000
Sequences
Ceph16S Rarefaction Curve
Families
2 4 6 8 10
0 5000 10000 15000 20000 25000 30000
Sequences
Ceph16S Rarefaction Curve
Genera
2 4 6 8 10 12 14
0 5000 10000 15000 20000 25000
Sequences
Mam16S Rarefaction Curve
Families
1 2 3 4 5 6
0 5000 10000 15000 20000 25000
Sequences
Mam16S Rarefaction Curve
Genera
1 2 3 4 5
0 5000 10000 15000 20000 25000
Sequences
trnL Rarefaction Curve
Families
2 4 6 8 10
0 10000 20000 30000
Sequences
Sym23S Rarefaction Curve
Families
0.6 0.8 1.0 1.2 1.4
0 50000 100000 150000
Sequences

Supplementary Data 6. Primer sets used in the amplification of eDNA sampled from Coral Bay seawater.

Primer Name	Oligonucleotide Sequence	PCR Annealing Temp.°C	Target taxa	Region	Amplicon Size (bp)	Primer Reference
18S_1F<br>18S_400R	5′ GCCAGTAGTCATATGCTTGTCT 3′<br>5′ GCCTGCTGCCTTCCTT 3′	52, 58, 64	Eukaryotes	nuclear<br>18S rDNA	336-423	Pochon et al. 2013
18S_V4F<br>18S_V4R	5′ GCAGTTAAAAAGCTCGTAG 3′<br>5′ TCCAAGAATTRCACCTCT 3′	50, 55, 60	Eukaryotes	nuclear<br>18S rDNA	240-420	This study
mlCOIintF<br>jgHCO2198	5′ GGWACWGGWTGAACWGTWTAYCCYCC 3′<br>5′ TAIACYTCIAAYCAYAARGAYATTGG 3′	46, 51, 56	Metazoans	mitochondria<br>COI	304-313	Leray et al. 2013
16SF/D<br>16S2R-degenerate	5′ GACCCTATGGAGCTTTAGAC 3′<br>5′ CGCTGTTATCCCTADRGTAACT 3′	54	Fish	mitochondria<br>16S rDNA	178-228	Berry et al. 2017<br>Deagle et al. 2007
16Smam1<br>16Smam2	5′ CGGTTGGGGTGACCTCGGA 3′<br>5′ GCTGTTATCCCTAGGGTAACT 3′	57	Mammals	mitochondria<br>16S rDNA	86-116	Taylor 1996
Crust16S_F(short)<br>Crust16S_R(short)	5′ GGGACGATAAGACCCTATA 3′<br>5′ ATTACGCTGTTATCCCTAAAG 3′	51	Crustaceans	mitochondria<br>16S rDNA	90-213	Berry et al. 2017
S_Cephalopoda_F<br>S_Cephalopoda_R	5′ GCTRGAATGAATGGTTTGAC 3′<br>5′ TCAWTAGGGTCTTCTCGTCC 3′	50	Cephalopods	mitochondria<br>16S rDNA	70-73	Peters et al. 2014
23SHYPERUP<br>23SHYPERDN	5′ TCAGTACAAATAATATGCTG 3′<br>5′ TTATCGCCCCAATTAAACAGT 3′	50	*Symbiodinium*	chloroplast<br>23S rDNA	122-163	Santos et al. 2003<br>Manning & Gates 2008
trnLc<br>trnLh	5′ CGAAATCGGTAGACGCTACG 3′<br>5′ CCATTGAGTCTCTGCACCTATC 3′	52	Plants	chloroplast<br>trnL	122-157	Taberlet et al. 1991<br>Taberlet et al. 2007
515F<br>R806	5′ GTGCCAGCMGCCGCGGTAA 3′<br>5′ GGACTACHVGGGTWTCTAAT 3′	50	Prokaryotes	16S rDNA	253-254	Turner et al. 1999<br>Caporaso et al. 2011

Berry, T. E. *et al*. DNA metabarcoding for diet analysis and biodiversity: a case study using the endangered Australian sea lion (*Neophoca cinerea*). *Ecol Evol* **7**, 5435-5453 (2017).

Caparaso, G.J. *et al.* Global patterns of 16S rRNA diversity at a depth of millions of sequences per sample. *PNAS* **108,** S4516-4522 (2011).

Deagle, B. E. *et al.* Studying seabird diet through genetic analysis of faeces: A case study on macaroni penguins (*Eudyptes chrysol*ophus). *PLoS One* **2**, e831 (2007).

Leray, M. *et al*. A new versatile primer set targeting a short fragment of the mitochondrial COI region for metabarcoding metazoan diversity: application for characterizing coral reef fish gut contents. *Front Zool* **10**,34 (2013).

Manning, M. M., Gates, R. D. Diversity in populations of free-living *Symbiodinium* from a Caribbean and Pacific reef. *Limnol Oceanograph* **53**, 1853-1861 (2008).

Peters, K. J., Ophelkeller, K., Herdina, Bott, N. J., Goldsworthy, S. D. PCR-based techniques to determine diet of the Australian sea lion (*Neophoca cinerea*): a comparison with morphological analysis. *Mar Ecol* **36**, 1428-1439 (2015).

Pochon, X., Bott, N. J., Smith, K. F., Wood, S. Evaluating detection limits of next-generation sequencing for the surveillance and monitoring of international marine pests. *PLoS One* **8**, e73935 (2013).

Santos, S. R., Gutierrez-Rodriguez, C., Coffroth, M. A. Phylogenetic identification of symbiotic dinoflagellates via length heteroplasmy in domain V of chloroplast large subunit (cp23S)-Ribosomal DNA sequences. *Mar Biotechnol* **5**, 130-140 (2003).

Taberlet, P., Gielly, L., Pautou, G., Bouvet, J. Universal primers for amplification of three regions of chloroplast DNA. *Plant Mol Biol* **17**, 1105-1109 (1991).

Taberlet, P., *et al.* Power and limitations of the chloroplast *trn*L (UAA) intron for plant DNA barcoding. *Nucleic Acids Res* **35**, e14 (2007).

Turner, S., Pryer, K. M., Miao, V. P. W., Palmer, J. D. Investigating deep phylogenetic relationships among cyanobacteria and plastids by small subunit rRNA sequence analysis. *J Eukary Microbiol* **46**, 327-338 (1999).
